# Supplementary material for: Neutralizing monoclonal antibodies elicited by mosaic RBD nanoparticles bind conserved sarbecovirus epitopes
Source: Immunity. 2022 Dec 13;55(12):2419–2435.e10. doi: 10.1016/j.immuni.2022.10.019 (PMC9606073; doi:10.1016/j.immuni.2022.10.019)
Supplement: Document S1. Figures S1–S7, Tables S1–S3, and supplemental references [file mmc1.pdf]

Immunity, Volume *55*

Supplemental information

Neutralizing monoclonal antibodies elicited by mosaic RBD nanoparticles bind conserved sarbecovirus epitopes

Chengcheng Fan, Alexander A. Cohen, Miso Park, Alfur Fu-Hsin Hung, Jennifer R. Keeffe, Priyanthi N.P. Gnanapragasam, Yu E. Lee, Han Gao, Leesa M. Kakutani, Ziyan Wu, Harry Kleanthous, Kathryn E. Malecek, John C. Williams, and Pamela J. Bjorkman

Supplemental figures

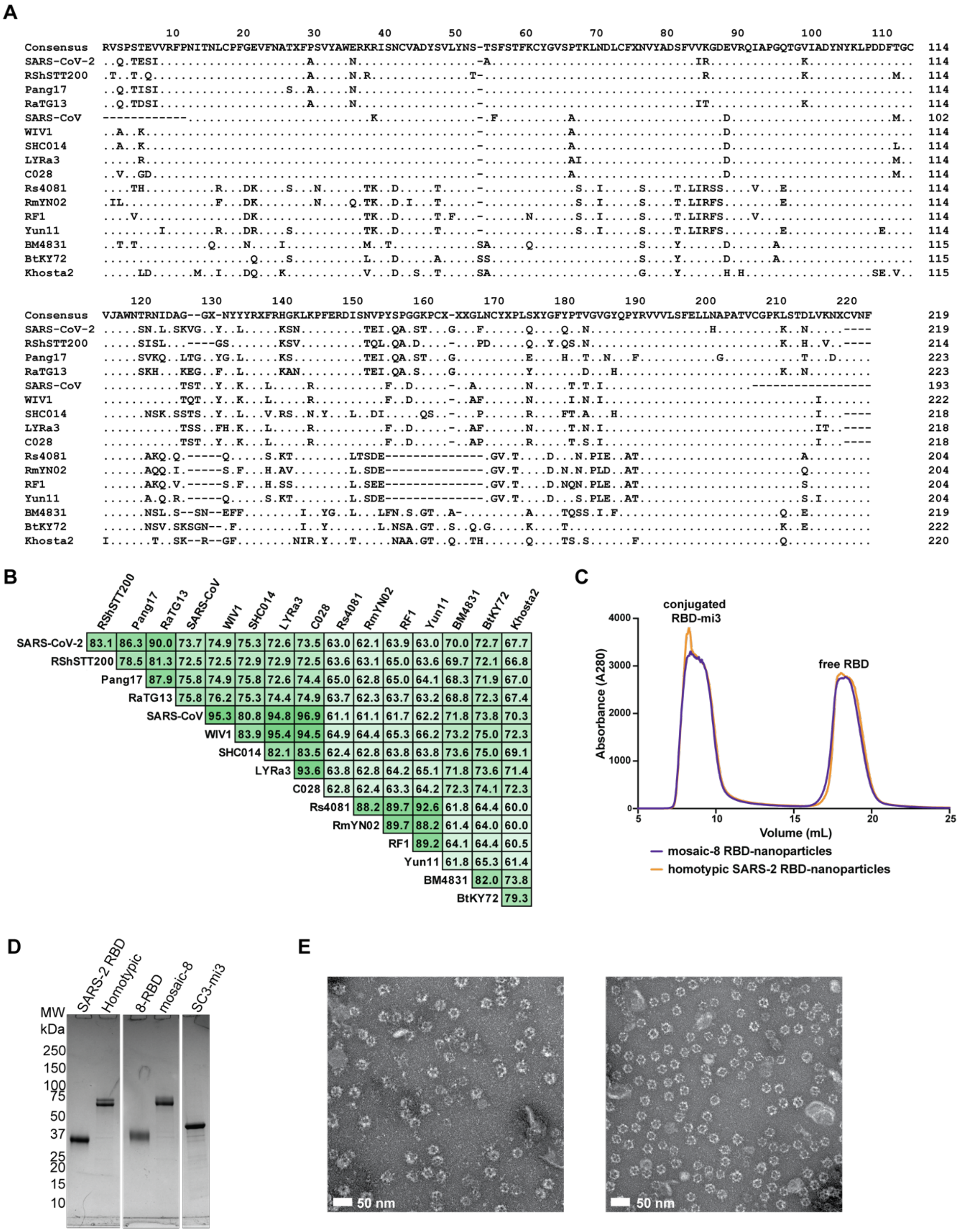

Figure S1. Sarbecovirus RBDs and construction of RBD-nanoparticles. Related to Figure 1.

(**A**) Sequence alignment of RBDs from 16 sarbecoviruses. (**B**) Pairwise sequence identities (%) relating the RBDs in panel A. (**C**) SEC profile showing separation of conjugated SpyCatcher003-mi3 nanoparticles from free RBDs. RBDs were added at a 2-fold molar excess over SpyCatcher003-mi3 subunits. (**D**) Reducing SDS-PAGE analysis of free RBD, purified conjugated RBD-nanoparticles, and unconjugated SpyCatcher-mi3 nanoparticles. (**E**) Negative stain EM of conjugated nanoparticles. Left: mosaic-8 RBD-nanoparticles. Right: homotypic SARS-CoV-2 RBD-nanoparticles.

A

```
                                                   CDRH1                       CDRH2
Consensus          QVQLQQPGAELVKPGASVKLSCKASGYTFTXYWMHWVKQRPGQGLEWIGXIYPXDGYTKYNE    62
M8a-3(IgHV1-69)    ............L.................N...........H......E.D.F.T.I.I.Q    62
M8a-6(IgHV1-69)    ........T...M..........T......H...........E......E.A.S.N.V...Q    62
M8a-28(IgHV1-55)   ...................M.......N.NH..IS..............D...LSHF.T...    62
M8a-31(IgHV14-3)   E...K.SV....R......V..T...FNIKNIY........E...D...R.D.AN.NSR.AP    62
M8a-34(IgHV1-55)   ...................M.........IT..IT..............D...GG.R.N...    62
HSW-1(IgHV1-64)    ...............T.M............S..................M.H.NS.S.....    62
HSW-2(IgHV1-82)    ......S.P..........I.......V.STS..S.......E.P....R...R..HSSSTG    62

                                                            CDRH3
Consensus          KFKXKATLTVDTSSSTAYMQLSSLTSEDSAVYYCARPDX---XXYXYFDYWGQGTTLTVSS    120
M8a-3(IgHV1-69)    ...G.S................................S--SGYPV...............    121
M8a-6(IgHV1-69)    ...G.S..S..R....................F.....N--SGYPV.........S.....    121
M8a-28(IgHV1-55)   ..TNR.........T......N....D....F....W.YFD---SRT..............    120
M8a-31(IgHV14-3)   ..QD...I.A....N...L.........T.I....DEGW-------G.AN.....LV...A    116
M8a-34(IgHV1-55)   ...S.................R..............Y.GNYVGY.YAM.......SV....    123
HSW-1(IgHV1-64)    N..S.......K........F.............V.SGSYYGTT.D...............    123
HSW-2(IgHV1-82)    ...D.....A.K..N...IH............F...DYG-----.Y...............    118

                                                CDRL1                    CDRL2
Consensus          DIVMTQSPASLSVSLGERVTISCRASQSV------GXFIAWYQQKPGQSPKLLIYXASTXXS    56
M8a-3(IgκV6-25)    .......HKFM.T.V.D..S.T.K...D.------.TY.................W...RHT    56
M8a-6(IgκV6-25)    .......QKFM.T...D..S...K...D.------.TTV................W...RHT    56
M8a-28(IgκV5-48)   ..LL..F..I....P....SF......TI------.TN.H....RING..R...KY..ESI.    56
M8a-31(IgκV8-19)   ........S..T.TA..K..M..KS...LLNSGNQKNYLT.....V..P......W...RDP    62
M8a-34(IgκV3-5)    ...L....V..A....Q.A.......E..DFYG--NS..Y........A......R..NLE.    60
HSW-1(IgκV3-5)     ...L.......A....Q.A.......E..NIYG--NS.MH........P.....FR..NLE.    60
HSW-2(IgκV12-44)   ..Q.........A.V..A...T..L.EN.------YS.L......Q.K..Q..V.R.K.LAE    56

                                                       CDRL3
Consensus          GVPDRFSGSGSGTDFTLTIXSVZAEDLATYYCQQHYSTPXTFGGGTKLEIK              107
M8a-3(IgκV6-25)    ......T......NY....S..Q.....L.H........Y...........              107
M8a-6(IgκV6-25)    ......T.T.....Y....S..A.....L.......N..Y..........E              107
M8a-28(IgκV5-48)   .I.S...........S.S.NN.ES..I.D.....IN.W.L...A....DL.              107
M8a-31(IgκV8-19)   ......T...F........S..Q.....V....ND..Y.L...A...V.L.              113
M8a-34(IgκV3-5)    .I.A.......R.......HP.E.D.V.......SIED.R...........              111
HSW-1(IgκV3-5)     .I.V.......R.......NP.E.D.V.....H.SNED.F...S.......              111
HSW-2(IgκV12-44)   ...S.........Q.S.K.N.LQP..FG.....H..G..P...........              107
```

B

	M8a-6 V_H	M8a-28 V_H	M8a-31 V_H	M8a-34 V_H	HSW-1 V_H	HSW-2 V_H
M8a-3 V_H	87.6	70.7	58.7	74.8	76.4	69.4
M8a-6 V_H		67.5	53.7	72.4	72.4	68.6
M8a-28 V_H			55.8	76.4	70.7	66.7
M8a-31 V_H				56.9	55.3	60.2
M8a-34 V_H					75.6	69.1
HSW-1 V_H						67.5

	M8a-6 V_L	M8a-28 V_L	M8a-31 V_L	M8a-34 V_L	HSW-1 V_L	HSW-2 V_L
M8a-3 V_L	89.7	54.2	62.8	57.7	54.1	60.7
M8a-6 V_L		53.3	61.1	58.6	55.9	57.0
M8a-28 V_L			50.4	55.0	56.8	52.3
M8a-31 V_L				55.8	54.9	52.2
M8a-34 V_L					88.3	56.8
HSW-1 V_L						55.9

C

	CDRH1	CDRH2	CDRH3	CDRL1	CDRL2	CDRL3
**M8a-3**	8	8	14	6	3	9
**M8a-6**	8	8	14	6	3	9
**M8a-28**	8	8	13	6	3	9
**M8a-31**	8	8	9	12	3	9
**M8a-34**	8	8	16	10	3	9
**HSW-1**	8	8	16	10	3	9
**HSW-2**	8	8	11	6	3	9

D

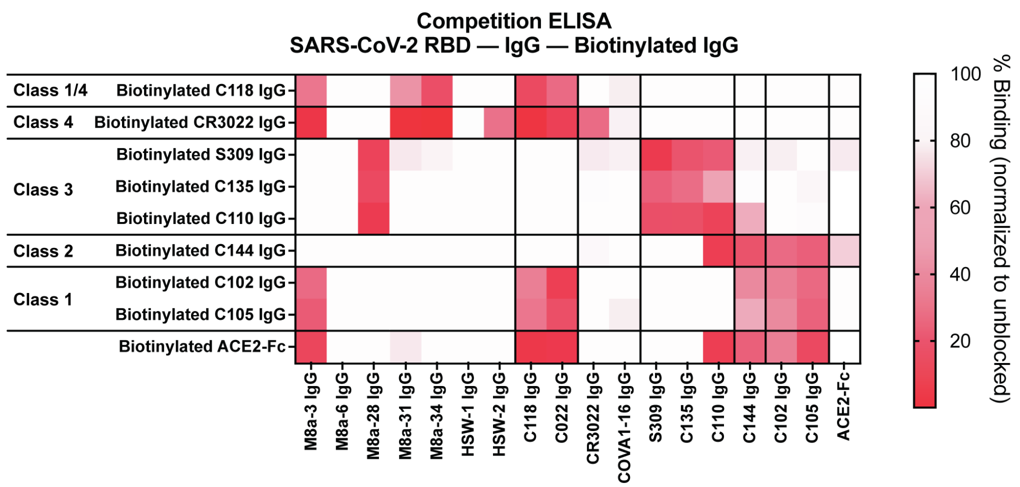

Figure S2. Sequence alignment of seven mAbs isolated from RBD nanoparticle-immunized mice that bound two or more sarbecovirus RBDs. Related to Figure 2.

(**A**) Sequence alignment of V_H and V_L domains. CDRs were assigned using IMGT definitions [S3]. VH and VL gene segment assignments for each mAb were shown in parentheses. (**B**) Pairwise

sequence identities (%) between V_H and V_L domains in (**A**). (**C**) Number of residues in CDRs. (**D**) Competition ELISA experiment in which a test set of biotinylated IgGs of known epitopes or human ACE2-Fc (y-axis) were assayed for binding to SARS-CoV-2 RBD in the presence of unlabeled M8a, HSW, control IgGs, or ACE2-Fc (x-axis). The heat map shows shades of red indicating the percent binding of the tested IgGs or ACE2-Fc in the presence of competitor. M8a-3, M8a-31, M8a-34, and HSW-2 IgGs competed with biotinylated class 1/4 and/or class 4 anti-RBD, with HSW-2 competing with the biotinylated class 4 antibody only. M8a-3 IgG competed with biotinylated class 1 antibodies and ACE2-Fc in addition to the biotinylated class 1/4 and class 4 antibodies. M8a-28 IgG competed with biotinylated class 3 antibodies. M8a-6 and HSW-1 IgGs showed no competition in this assay.

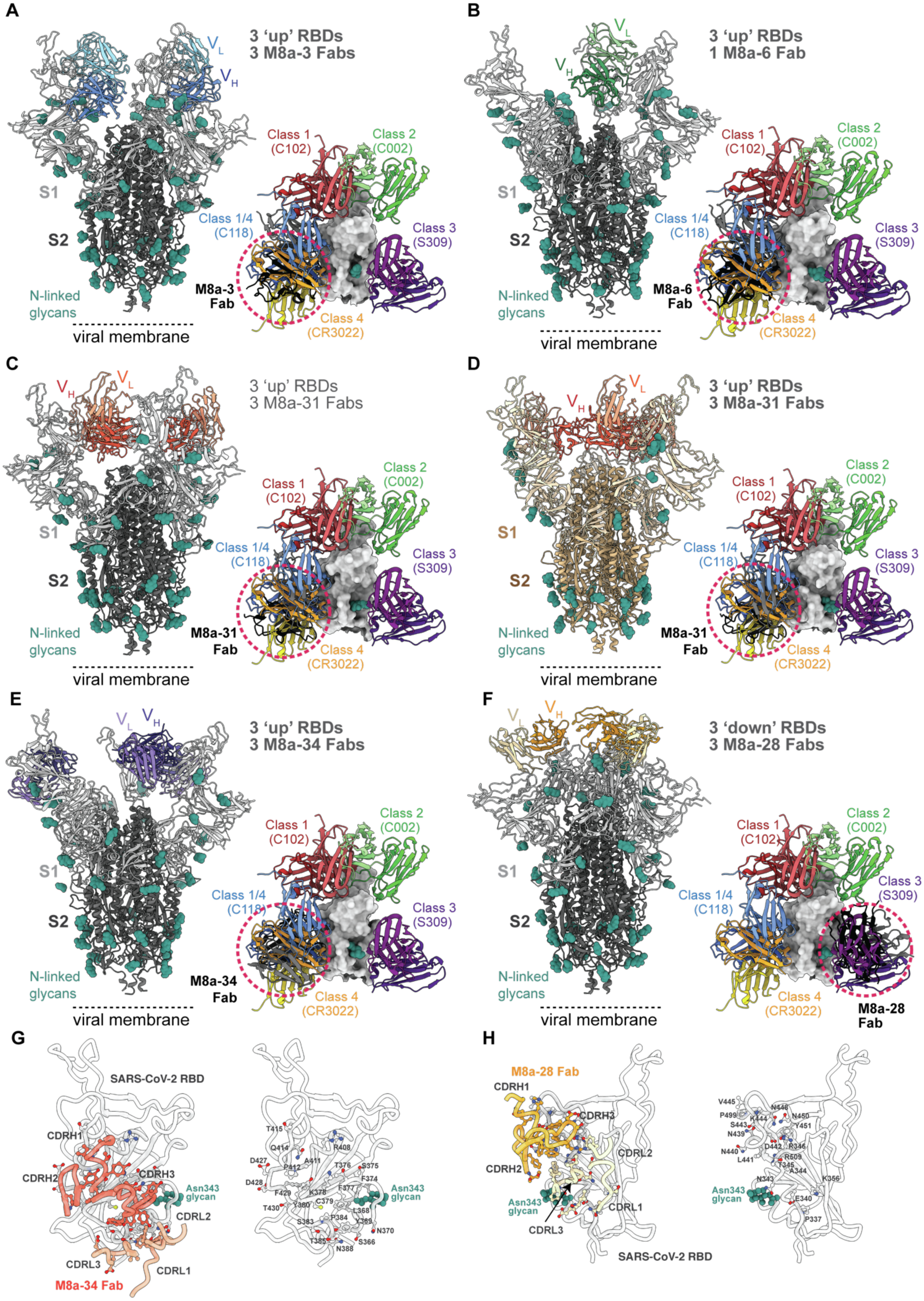
A
3 'up' RBDs
3 M8a-3 Fabs
V L
V H
S1
S2
N-linked glycans
viral membrane
Class 1 (C102)
Class 2 (C002)
Class 1/4 (C118)
Class 3 (S309)
M8a-3 Fab
Class 4 (CR3022)
B
3 'up' RBDs
1 M8a-6 Fab
V L
V H
S1
S2
N-linked glycans
viral membrane
Class 1 (C102)
Class 2 (C002)
Class 1/4 (C118)
Class 3 (S309)
M8a-6 Fab
Class 4 (CR3022)
C
3 'up' RBDs
3 M8a-31 Fabs
V H
V L
S1
S2
N-linked glycans
viral membrane
Class 1 (C102)
Class 2 (C002)
Class 1/4 (C118)
Class 3 (S309)
M8a-31 Fab
Class 4 (CR3022)
D
3 'up' RBDs
3 M8a-31 Fabs
V L
V H
S1
S2
N-linked glycans
viral membrane
Class 1 (C102)
Class 2 (C002)
Class 1/4 (C118)
Class 3 (S309)
M8a-31 Fab
Class 4 (CR3022)
E
3 'up' RBDs
3 M8a-34 Fabs
V L
V H
S1
S2
N-linked glycans
viral membrane
Class 1 (C102)
Class 2 (C002)
Class 1/4 (C118)
Class 3 (S309)
M8a-34 Fab
Class 4 (CR3022)
F
3 'down' RBDs
3 M8a-28 Fabs
V L
V H
S1
S2
N-linked glycans
viral membrane
Class 1 (C102)
Class 2 (C002)
Class 1/4 (C118)
Class 3 (S309)
Class 4 (CR3022)
M8a-28 Fab
G
SARS-CoV-2 RBD
CDRH1
CDRH2
CDRH3
Asn343 glycan
CDRL2
CDRL3
CDRL1
M8a-34 Fab
T415
Q414
R408
D427
A411
P412
T376
S375
F374
D428
F377
F429
K378
C379
Y380
T430
S383
P384
L368
Y369
N370
T385
S366
N388
Asn343 glycan
H
M8a-28 Fab
CDRH1
CDRH3
CDRL2
CDRH2
Asn343 glycan
CDRL1
CDRL3
SARS-CoV-2 RBD
V445
N448
P499
K444
N450
S443
Y451
N439
D442
R346
N440
L441
R509
T345
A344
N343
K356
Asn343 glycan
E340
P337

Figure S3. Structural analysis of mAbs isolated from mice immunized with mosaic-8 nanoparticles. Related to Figure 3.

Cartoon representations of single particle cryo-EM structures of Fab-spike trimer complexes are shown from the side (left) with a comparison of binding epitopes of the Fab with representative anti-RBD antibodies: class 1 (C102, PDB 7K8M), class 2 (C002, PDB 7K8T), class 3 (S309, PDB 7JX3), class 4 (CR3022, PDB 7LOP), and class 1/4 (C118, PDB 7RKV)) aligned on an RBD in surface representation (right). Only V_H-V_L domains are shown for each Fab. Fabs of interests (colored in black and circled with a red dotted line) and the anti-RBD antibodies used for classification are aligned on a surface representation of the RBD. N-linked glycans are shown as teal spheres. (**A**) WA1 spike complexed with M8a-3. (**B**) SARS-CoV-2 WA1 spike complexed with M8a-6. (**C**) WA1 spike complexed with M8a-31. (**D**) Omicron BA.1 spike complexed with M8a-31. (**E**) WA1 spike complexed with M8a-34. (**F**) WA1 spike complexed with M8a-28. Left: interactions between SARS-CoV-2 RBD and the CDRs of (**G**) M8a-28 Fab and (**H**) M8a-34 Fab. Right: SARS-CoV-2 RBD residues involved in interactions with (**G**) M8a-28 Fab and (**H**) M8a-34 Fab.

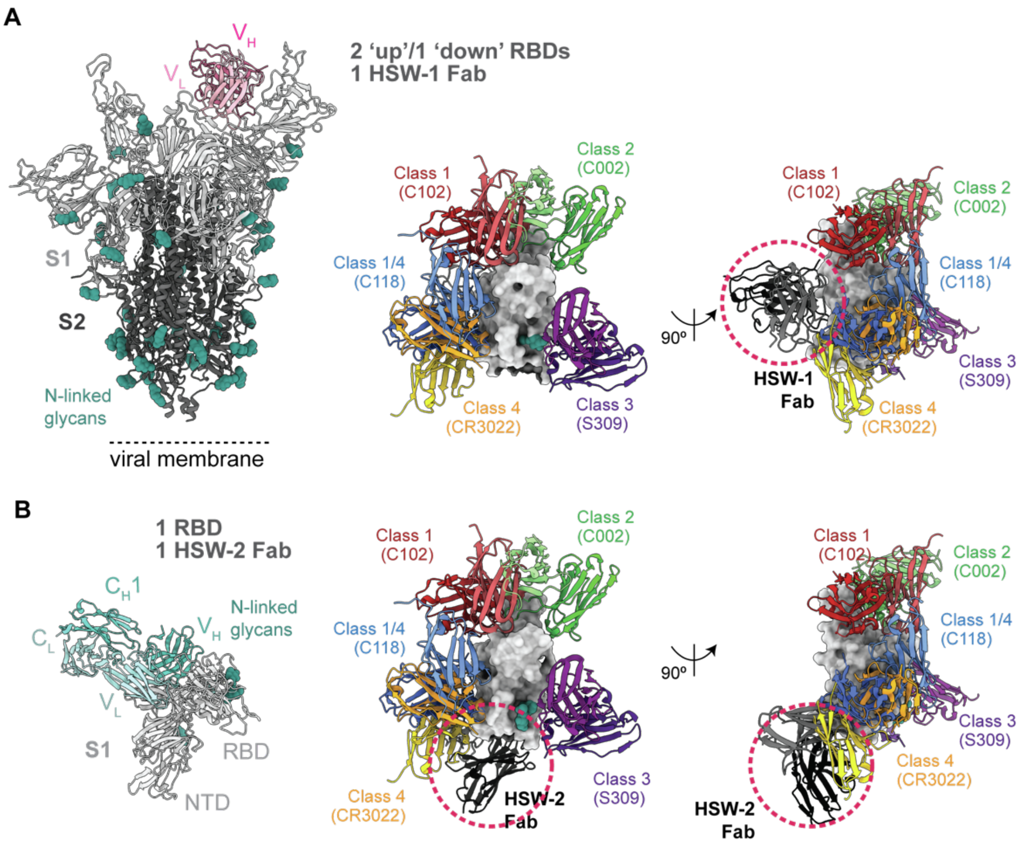

Figure S4. Structural analysis of mAbs isolated from mice immunized with homotypic nanoparticles. Related to Figure 4.

Cartoon representations of single particle cryo-EM structures of Fab-spike trimer complexes are shown from the side (left) with a comparison of binding epitopes of the Fab with representative anti-RBD antibodies: class 1 (C102, PDB 7K8M), class 2 (C002, PDB 7K8T), class 3 (S309, PDB 7JX3), class 4 (CR3022, PDB 7LOP) and class 1/4 (C118, PDB 7RKV)) aligned on an RBD in surface representation (middle and right). Only V_H-V_L domains are shown for each Fab. Fabs of interests (colored in black and circled with a red dotted line) and the anti-RBD antibodies used for classification are aligned on a surface representation of the RBD. N-linked glycans are shown as teal spheres. (**A**) SARS-CoV-2 WA1 spike complexed with HSW-1. (**B**) WA1 spike S1 domain complexed with HSW-2.

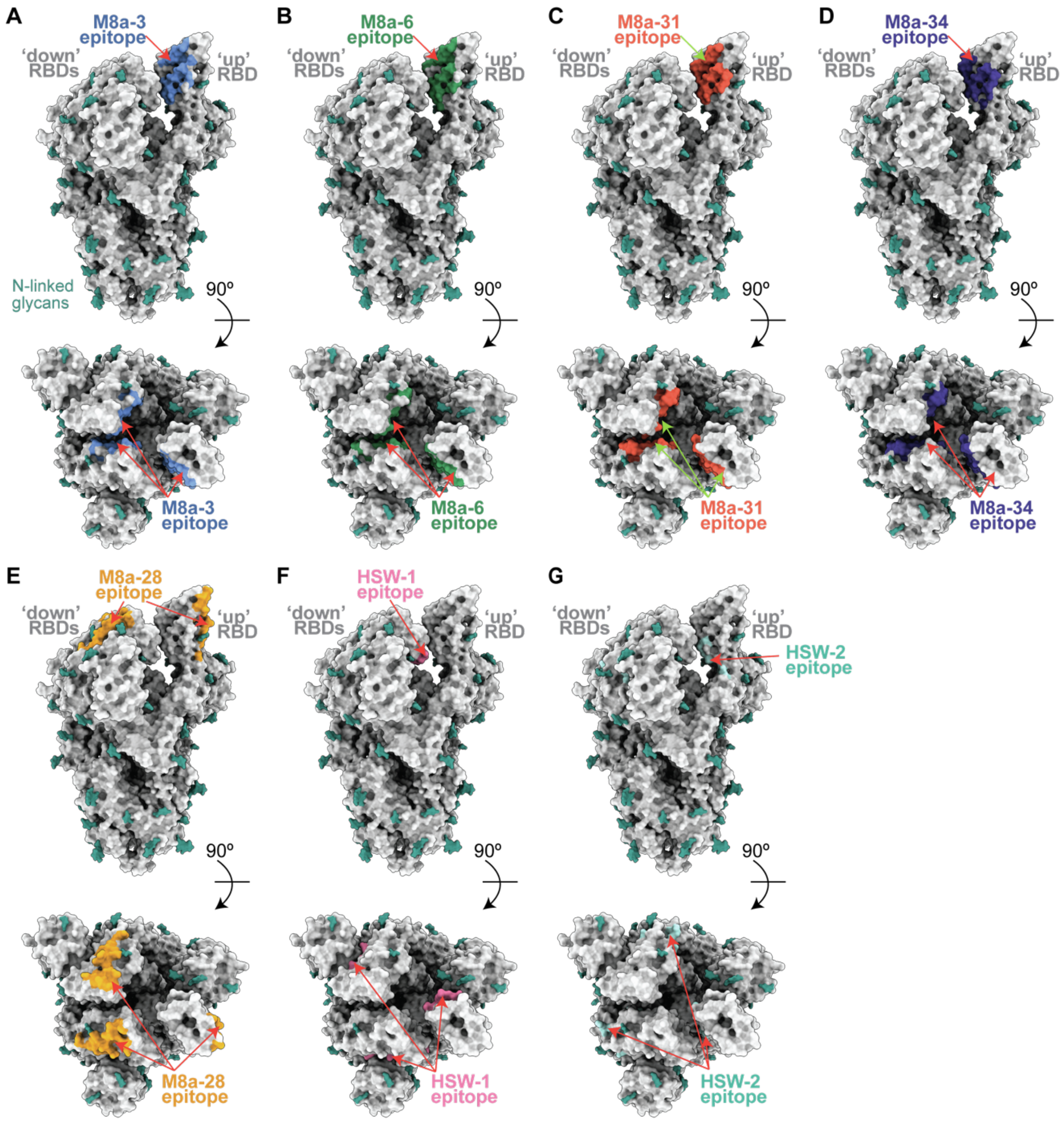

Figure S5. Epitopes of mAbs mapped on a unliganded SARS-CoV-2 spike trimer. Related to Figure 5 and 6.

Binding epitopes for all mAbs were identified by PDBePISA [S2] and then mapped on to a unliganded spike trimer with two 'down' and one 'up' RBDs (PDB 6VYB). The spike trimer is shown as a surface representation with N-linked glycans shown as teal spheres. The epitopes of (**A**) M8a-3, (**B**) M8a-6, (**C**) M8a-31 and (**D**) M8a-34 are blocked in the 'down' RBD conformation, but accessible in an 'up' RBD conformation. (**E**) The epitope of M8a-28 is accessible in both 'down' and 'up' RBD conformations. (**F**) The epitope of HSW-1 is blocked in the 'down' RBD

conformation, but accessible in an ‘up’ RBD conformation. (**G**) The epitope of HSW-2 is blocked in both ‘down’ and ‘up’ RBD conformations.

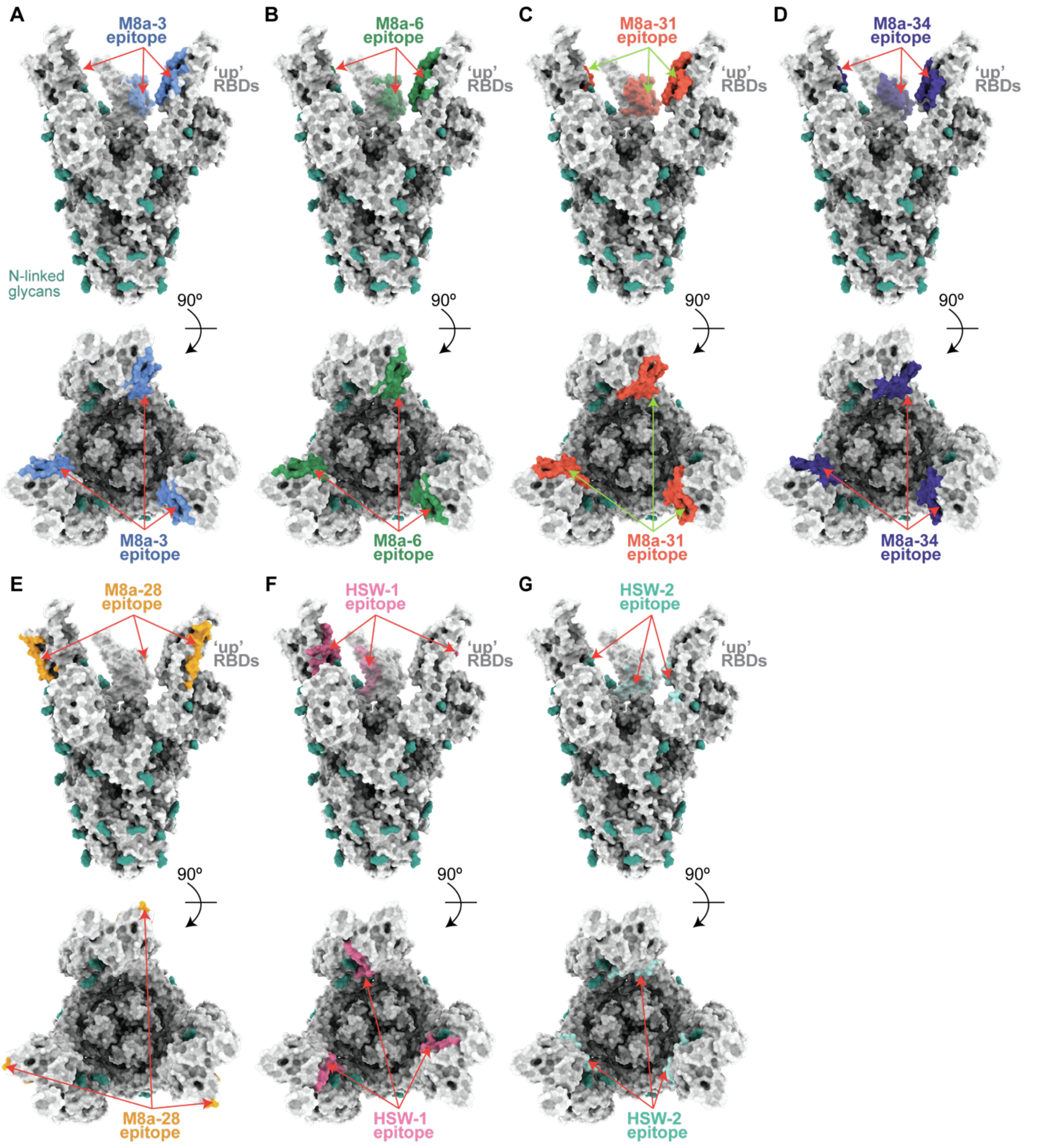

Figure S6. Epitopes of mAbs mapped on a SARS-CoV-2 spike trimer with all 'up' RBDs. Related to Figure 5 and 6.

Binding epitopes for all mAbs were identified by PDBePISA [S2] and then mapped on to a spike trimer with three 'up' RBDs (PDB 7RKV; Fabs not shown). The spike trimer is shown as a surface representation with N-linked glycans shown as teal spheres. The epitopes of (**A**) M8a-3, (**B**) M8a-6, (**C**) M8a-31, and (**D**) M8a-34 are all accessible in an 'up' RBD conformation. (**E**) The epitope

of M8a-28 is accessible in ‘up’ RBD conformation. (**F**) The epitope of HSW-1 is accessible in an ‘up’ RBD conformation. (**G**) The epitope of HSW-2 is sterically hindered in an ‘up’ RBD conformation.

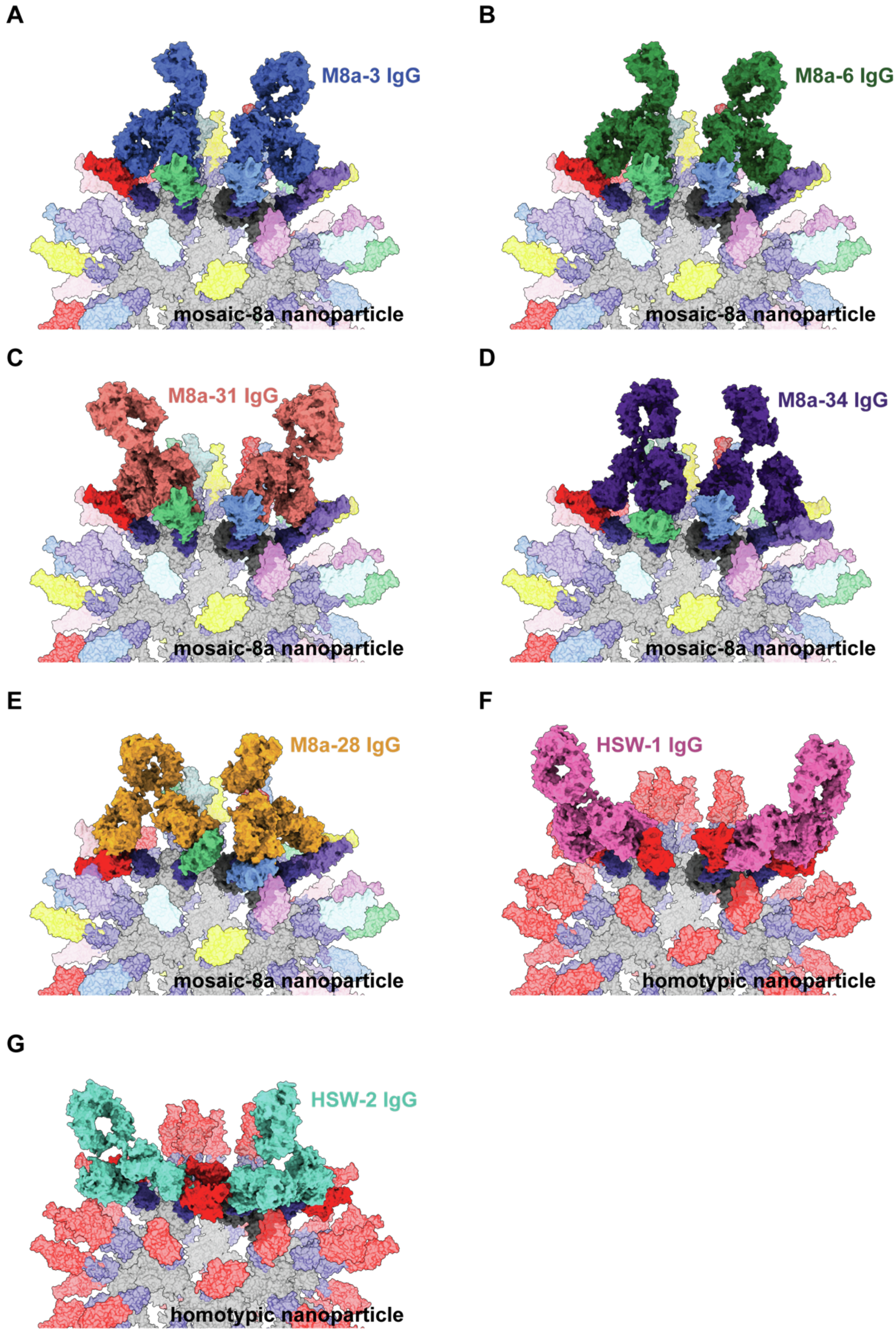

Figure S7. Models of M8a and HSW IgGs binding to adjacent RBDs on SpyCatcher-mi3 nanoparticles. Related to Figure 6.

Structural models of RBD-nanoparticles formed by SpyCatcher-mi3 and SpyTagged RBDs were made using coordinates of an RBD (PDB 7SC1) (represented in different colors for mosaic-8 nanoparticles, with two adjacent RBDs in red and blue, or in red for homotypic nanoparticles), mi3 (PDB 7B3Y) (gray), and SpyCatcher (PDB 4MLI) (dark blue). IgGs (various colors) were modeled using the coordinates of each mAb Fab based on an intact IgG crystal structure (PDB 1HZH), and the RBD binding epitope of each Fab in a modeled IgGs was determined based on the Fab-spike structures reported in this study. The two Fabs of each IgGs were positioned with distances between the C-termini of the Fab C_H1 domains to be less than 65 Å, as described previously [S1]. Both the IgG Fc hinge and the linker region between a SpyTagged RBD and SpyCatcher were assumed to be flexible and adjusted accordingly. Models are shown for (**A**) M8a-3 IgG interacting with adjacent RBDs on a mosaic-8 RBD-nanoparticle. (**B**) M8a-6 IgG interacting with adjacent RBDs on a mosaic-8 RBD-nanoparticle. (**C**) M8a-31 IgG interacting with adjacent RBDs on a mosaic-8 RBD-nanoparticle. (**D**) M8a-34 IgG interacting with adjacent RBDs on a mosaic-8 RBD-nanoparticle. (**E**) M8a-28 IgG interacting with adjacent RBDs on a mosaic-8 RBD-nanoparticle. (**F**) HSW-1 IgG interacting with adjacent RBDs on a homotypic RBD-nanoparticle. (**G**) HSW-2 IgG interacting with adjacent RBDs on a homotypic RBD-nanoparticle.

Supplemental Tables

mAbs	V_H (sequences)	V_L (sequences)	Lineages
**M8a-3**	QVQLQQPGAELVLPGASVKLSCKASGYTFTNYWMHWVKQRPG<br>HGLEWIGEIDPFDTYIKINQKFKGKSTLTVDTSSSTAYMQLS<br>SLTSEDSAVYYCARPDSSGYPVYFDYWGQGTTLTVSS	DIVMTQSHKFMSTSVGDRVSITCKASQDVGTYIAWYQQKPGQ<br>SPKLLIYWASTRHTGVPDRFTGSGSGTNYTLTISSVQAEDLA<br>LYHCQQHYSTPYTFGGGTKLEIK	IgHV1-69<br>IgκV6-25
**M8a-6**	QVQLQQPGTELVMPGASVKLSCKTSGYTFTHYWMHWVKQRPG<br>EGLEWIGEIAPSDNYVKYNQKFKGKSTLSVDRSSSTAYMQLS<br>SLTSEDSAVYFCARPDNSGYPVYFDYWGQGTSLTVSS	DIVMTQSQKFMSTSLGDRVSISCKASQDVGTTVAWYQQKPGQ<br>SPKLLIYWASTRHTGVPDRFTGTGSGTDYTLTISSVAAEDLA<br>LYYCQQHYNTPYTFGGGTKLEIE	IgHV1-69<br>IgκV6-25
**M8a-7**	QAYLQQSGAEMVRPGASVKMSCKASGYTFNNYNMHWVKQTPS<br>QGLEWIGGFYPGNDDTAYSQKFKGKATLTVDKSSSTAFMHLS<br>SLTSEDSAVYFCARSLGRYYAMDYWGQGTSVTVSS	DIVLTQSPASLAVSLGQRATISCRASESVDDFGISYMNWFQQ<br>KPGQTPKLLIYGASNQGSGVPARFSGSGSGTDFSLNIHPMEE<br>DDPAMYFCQQSKEVPYTFGGGTKLEIK	IgHV1-12<br>IgκV3-2
**M8a-9**	QVQLQQPGAELVRPGSSVKLSCKASGYTFTSYWIHWVRQRPI<br>EGPEWIGMIDPSDSGNHFNQNFKDKATWTVDKSSNTAYMQLS<br>SLTTEDSAVYYCARGSGSTYRGYFDYWGHGTTLTVSS	DIQMTQSSSYLSVSLGGRVTITCKASDHINNWLAWYQQKPGN<br>TPRLLISGATNLETGVPSRFSGSGSGKDYTLSITSLQTEDVA<br>TYYCQQYWSSPLTFGAGTKLELK	IgHV1-52<br>IgκV13-85
**M8a-11**	EVQLQQSGPELVKPGASVKIPCKASGYTFTDYNMDWVKQSHE<br>KSLEWIGEIDPNNGDTIYNQKFKGKASLTVDKSSSTAYMELR<br>SLTSEDTAVYYCAKRGYYGSSLWWYFDVWGTGTTVTVSS	DIQMTQSSSSFSVSLGDRVTITCKASEDIYIRLAWYQQRPGN<br>APRLLISNAISLETGVPSRFSGSGSGKDYTLSITSLQTEDVA<br>TYYCQQYWSTPWTFGGGTKLEIK	IgHV1-18<br>IgκV13-84
**M8a-15**	QVQLQQSGPELARPGASVKLSCRASGYTVTSFGLSWMKQRTG<br>QGLEWIGEIYPTSKNTYYNDKFRTKATLTADKSSSTAYMELR<br>SLTSEDSAVYFCVLYDYFDYWGQGTTLTVSS	QIVLTQSPAIMSASPGEKVTISCSASSSVSYMYWYQQKTGSS<br>PKPWIYRTSNLASGVPVRFSGSGSGTSYSLTISSMEAEDAAT<br>YYCQQYQSYPRTFGGGTKLEIK	IgHV1-81<br>IgκV4-61
**M8a-25**	EVQLQQSVAELVRPGASVKLSCTASGFNIKNTYMHWVKQRPE<br>QGLEWIGRIDPSIDHTRYAPKFQGKAVITAFTSSNTAYLQLS<br>SLTSEDTAIYYCAREGGGNYPYYYAIDYWGQGTSVTVSS	DIQMTQSSSSFSVSLGDRVTITCKASEDIYIRLAWYQQRPGN<br>APRLLISNAISLETGVPSRFSGSGFGKDHTLSITSLQTEDVA<br>TYYCQQYWSTPWTFGGGTKLEIK	IgHV14-3<br>IgκV13-84
**M8a-28**	QVQLQQPGAELVKPGASVKMSCKASGYNFNHYWISWVKQRPG<br>QGLEWIGDIYPLSHFTTYNEKFTNRATLTVDTSSTTAYMQLN<br>SLTSDDSAVFYCARWDYFDSRTFDYWGQGTTLTVSS	DILLTQFPAILSVSPGERVSFSCRASQTIGTNIHWYQQRING<br>SPRLLIKYASESISGIPSRFSGSGSGTDFSLSINNVESEDIA<br>DYYCQQINSWPLTFGAGTKLDLK	IgHV1-55<br>IgκV5-48
**M8a-29**	EVQLQQSVAELVRPGASVKLSCTASGFNIKNTYMHWVKQRPE<br>QGLEWIGRIDPSIDHTRYAPKFQGKAVITAFTSSNTAYLQLS<br>SLTSEDTAIYYCAREGGGNYPYYYAIDYWGQGTSVTVSS	DIVMTQAAFSNPVTLGTSASISCRSTKSLLHSNGITYLYWYL<br>QKPGQSPQLLIYQMSNLASGVPDRFSSSGSGTDFTLRISRVE<br>AEDVGVYYCAQNLELPYTFGGGTKLEIK	IgHV14-3<br>IgκV2-109
**M8a-30**	QVHLQQSGPELVKPGASVKISCKASGYGFSSSWMNWVKQRPG<br>KGLEWIGRIYPGDGDTNYNDKFQGKATLTADRSSSTAYMHLT<br>SLTSADSAVYFCARSLLYSFDYWGQGTTLTVSS	DVVVTQTPLSLPVSFGDQVSISCRSSQSLAGSYGHTYLSWYL<br>HKSGQSPQLLIYGISNRFSGVPDRFSGSGSGTDFTLKISTIK<br>PEDLGMYYCLQGTHQPLTFGAGTKLELK	IgHV1-82<br>IgκV1-88
**M8a-31**	EVQLKQSVAELVRPGASVKVSCTASGFNIKNIYMHWVKQRPE<br>QGLDWIGRIDPANGNSRYAPKFQDKATITADTSSNTAYLQLS<br>SLTSEDTAIYYCADEGWGFANWGQGTLVTVSA	DIVMTQSPSSLTVTAGEKVTMSCKSSQSLLNSGNQKNYLTWY<br>QQKVGQPPKLLIYWASTRDPGVPDRFTGSGFGTDFTLTISSV<br>QAEDLAVYYCQNDYSYPLTFGAGTKVELK	IgHV14-3<br>IgκV8-19
**M8a-34**	QVQLQQPGAELVKPGASVKMSCKASGYTFITYWITWVKQRPG<br>QGLEWIGDIYPGGGRTNYNEKFKSKATLTVDTSSSTAYMQLR<br>SLTSEDSAVYYCARYDGNYVGYYYAMDYWGQGTSVTVSS	DIVLTQSPVSLAVSLGQRATISCRASESVDFYGNSFIYWYQQ<br>KPGQAPKLLIYRASNLESGIPARFSGSGSRTDFTLTIHPVEA<br>DDVATYYCQQSIEDPRTFGGGTKLEIK	IgHV1-55<br>IgκV3-5
**M8a-36**	QVQLQQTGAELVKPGASVKMSCKASGYTFTSYWIIWVKKRPG<br>QGLEWIGDIYPGSGSTNYNEKFKSKATLTVDTSSSTAYMQLS<br>SLTSEDSAVYYCTRGGSRFAMDYWGQGTSVTVSS	DIVMTQAAPSVPVTPGESVSISCRSSKSLLHSNGNTYLYWFL<br>QRPGQSPQLLIYRMSNLASGVPDRFSGSGSGTAFTLRISRVE<br>AEDVGVYYCMQHLEYPYTFGGGTKLEIK	IgHV1-55<br>IgκV2-137
**HSW-1**	QVQLQQPGAELVKPGTSMKLSCKASGYTFTSYWMHWVKQRPG<br>QGLEWIGMIHPNSGSTKYNENFKSKATLTVDKSSSTAYMQFS<br>SLTSEDSAVYYCVRSGSYYGTTYDYFDYWGQGTTLTVSS	DIVLTQSPASLAVSLGQRATISCRASESVNIYGNSFMHWYQQ<br>KPGQPPKLLIFRASNLESGIPVRFSGSGSRTDFTLTINPVEA<br>DDVATYYCHQSNEDPFTFGSGTKLEIK	IgHV1-64<br>IgκV3-5
**HSW-2**	QVQLQQSGPELVKPGASVKISCKASGYVFSTSWMSWVKQRPG<br>EGPEWIGRIYPRDGHSSSTGKFKDKATLTADKSSNTAYIHLS<br>SLTSEDSAVYFCARDYGYYYFDYWGQGTTLTVSS	DIQMTQSPASLSASVGEAVTITCRLSENVYSFLAWYQQKQGK<br>SPQLLVYRAKTLAEGVPSRFSGSGSGTQFSLKINSLQPEDFG<br>TYYCQHHYGTPPTFGGGTKLEIK	IgHV1-82<br>IgκV12-44

Table S1. Sequences and V gene segment lineages for 16 mAbs identified as binding at least one RBD during screening. Related to Figure 2. M8a-9 and M8a-36 did not exhibit binding to purified RBDs by ELISA (data not shown). M8a-11 and M8a-26 are identical sequences.

Structures	WA1 spike in complex with							Omicron BA.1 spike in complex with M8a-31 Fab
	M8a-3 Fab	M8a-6 Fab	M8a-28 Fab	M8a-31 Fab	M8a-34 Fab	HSW-1 Fab	HSW-2 Fab	
**Data Collection and processing**								
**Microscope**	Titan Krios	Titan Krios	Titan Krios	Titan Krios	Titan Krios	Titan Krios	Talos Arctica	Titan Krios
**Camera**	Gatan K3	Gatan K3	Gatan K3	Gatan K3	Gatan K3	Gatan K3	Gatan K3	Gatan K3
**Magnification**	105,000	105,000	105,000	105,000	105,000	105,000	45,000	105,000
**Voltage (keV)**	300	300	300	300	300	300	200	300
**Exposure (e/Å^2)**	60	60	60	60	60	60	60	60
**Pixel size (Å)**	0.832	0.832	0.832	0.832	0.832	0.832	0.869	0.832
**Defocus Range (μm)**	-1 to -3	-1 to -3	-1 to -3	-1 to -3	-1 to -3	-1 to -3	-1 to -3	-1 to -3
**Initial Particle Image (no.)**	1,026,209	842,602	964,614	670,746	1,186,807	1,713,001	449,736	1,689,653
**Final Particle Image (no.)**	272,779	159,604	332,106	295,711	421,879	336,288	43,362	142,452
**Symmetry Imposed**	C1	C1	C3	C3	C1	C1	C1	C3
**Map Resolution (Å)**	3.1	3.2	2.8	2.9	3.5	3.1	4.1	3.1
**FSC Threshold**	0.143	0.143	0.143	0.143	0.143	0.143	0.143	0.143
**Map Resolution Range (Å)**	3.0 - 3.5	3.1 - 3.5	2.7 to 3.1	2.9 - 3.2	3.5 - 4.3	3.0 to 3.4	3.9 - 4.3	2.9 - 3.3
**Refinement**								
**Initial Model Used**	PDB 7SC1	PDB 7SC1	PDB 7SC1	PDB 7SC1	PDB 7SC1	PDB 7SC1	PDB 7SC1	PDB 7SC1
**Model Resolution (Å)**	3.1	3.2	2.8	2.9	3.5	3.1	4.1	3.1
**FSC Threshold**	0.143	0.143	0.143	0.143	0.143	0.143	0.143	0.143
**Model composition**								
**non-hydrogen atoms**	30,762	27,051	30,942	30,552	30,669	26,992	8,392	30,075
**protein residues**	3,867	3,397	3,906	3,861	3,873	3,409	1,077	3,801
**ligands**	51	48	39	45	42	38	4	33
**Average B-factors (Å^2)**								
**protein**	135.2	157.5	110.4	130.4	205.7	158.8	197.9	92.4
**ligands**	116.3	139.9	96.8	111.6	167.3	143.6	213.5	82.5
**R.m.s. deviations**								
**Bond length (Å)**	0.005	0.004	0.003	0.004	0.003	0.004	0.003	0.004
**Bond angles (º)**	0.562	0.582	0.536	0.572	0.586	0.593	0.589	0.594
**Validation**								
**MolProbity score**	1.85	1.63	1.74	1.72	1.75	1.83	1.9	1.77
**Clashscore**	10.3	9.4	9.5	9.1	11.1	12.3	14.6	10.8
**Rotamer outliers**	0.06	0.07	0.03	0	0.03	0	0	0.06
**Ramachandran plot**								
**Ramachandran favored (%)**	95.4	97.3	96.4	96.5	96.9	96.5	96.4	96.6
**Ramachandran allowed (%)**	4.6	2.7	3..6	3.5	3.1	3.5	3.6	3.4
**Ramachandran outliers (%)**	0	0	0	0	0	0	0	0
**PDB ID**	7UZ4	7UZ5	7UZ6	7UZ7	7UZ9	7UZA	7UZB	7UZ8

Table S2. Single-particle cryo-EM data collection, processing, and refinement. Related to Figure 3 and 4.

Structures	SARS-CoV-2 RBD + M8a-34 Fab	SARS-CoV-2 RBD + HSW-2 Fab
Wavelength (Å)	0.97946	0.97946
Resolution range (Å)	39.23 - 2.2 (2.279 - 2.2)	39.08 - 3.0 (3.108 - 3.0)
Space group	$P2_1$	$P4_32_12$
Unit cell (Å, °)	54.5 165.2 93.7 90 106.6 90	123.6 123.6 145.6 90 90 90
Total reflections (no.)	553,434 (52,800)	592,837 (59,309)
Unique reflections (no.)	79,107 (7,859)	23,174 (2,178)
Multiplicity	7.0 (6.7)	25.6 (26.2)
Completeness (%)	98.5 (97.9)	99.5 (96.1)
Mean I/sigma(I)	12.2 (1.24)	19.6 (2.6)
Wilson B-factor ($Å^2$)	41.4	81.1
R-merge	0.105 (1.378)	0.147 (1.856)
R-meas	0.113 (1.494)	0.150 (1.893)
R-pim	0.0425 (0.568)	0.030 (0.366)
CC1/2	0.998 (0.642)	0.999 (0.842)
CC*	1 (0.884)	1 (0.956)
Reflections used in refinement (no.)	79,082 (7,845)	23,082 (2,178)
Reflections used for R-free (no.)	1,997 (195)	1,155 (109)
R-work	0.171 (0.270)	0.230 (0.360)
R-free	0.243 (0.329)	0.271 (0.458)
CC (work)	0.971 (0.821)	0.899 (0.834)
CC (free)	0.962 (0.771)	0.604 (0.753)
Number of non-hydrogen atoms	10,482	4,912
macromolecules	9,884	4,884
ligands	111	28
solvent	487	0
Protein residues (no.)	1,278	633
RMS (bonds) (Å)	0.008	0.009
RMS (angles) (°)	0.98	1.15
Ramachandran favored (%)	97.5	96.3
Ramachandran allowed (%)	2.5	3.7
Ramachandran outliers (%)	0	0
Rotamer outliers (%)	0.9	0
Clashscore	2.6	6.1
Average B-factor ($Å^2$)	47	73.0
macromolecules	46.7	72.7
ligands	85.0	118.3
solvent	44.3	-
PDB ID	7UZC	7UZD

Table S3. X-ray crystallography data collection, processing, and refinement. Related to Figure 3 and 4.

References

Barnes, C.O., Jette, C.A., Abernathy, M.E., Dam, K.A., Esswein, S.R., Gristick, H.B., Malyutin, A.G., Sharaf, N.G., Huey-Tubman, K.E., Lee, Y.E., *et al*. (2020). SARS-CoV-2 neutralizing antibody structures inform therapeutic strategies. Nature *588*, 682-687.

Krissinel, E., and Henrick, K. (2007). Inference of macromolecular assemblies from crystalline state. J Mol Biol *372*, 774-797.

Lefranc, M.P., Giudicelli, V., Duroux, P., Jabado-Michaloud, J., Folch, G., Aouinti, S., Carillon, E., Duvergey, H., Houles, A., Paysan-Lafosse, T., *et al*. (2015). IMGT(R), the international ImMunoGeneTics information system(R) 25 years on. Nucleic Acids Res *43*, D413-422.
