# Supplementary material for: Neutralizing monoclonal antibodies elicited by mosaic RBD nanoparticles bind conserved sarbecovirus epitopes
Source: Immunity. 2022 Dec 13;55(12):2419–2435.e10. doi: 10.1016/j.immuni.2022.10.019 (PMC9606073; doi:10.1016/j.immuni.2022.10.019)
Supplement: Data S1. Additional supplemental figures and legends [file mmc2.pdf]

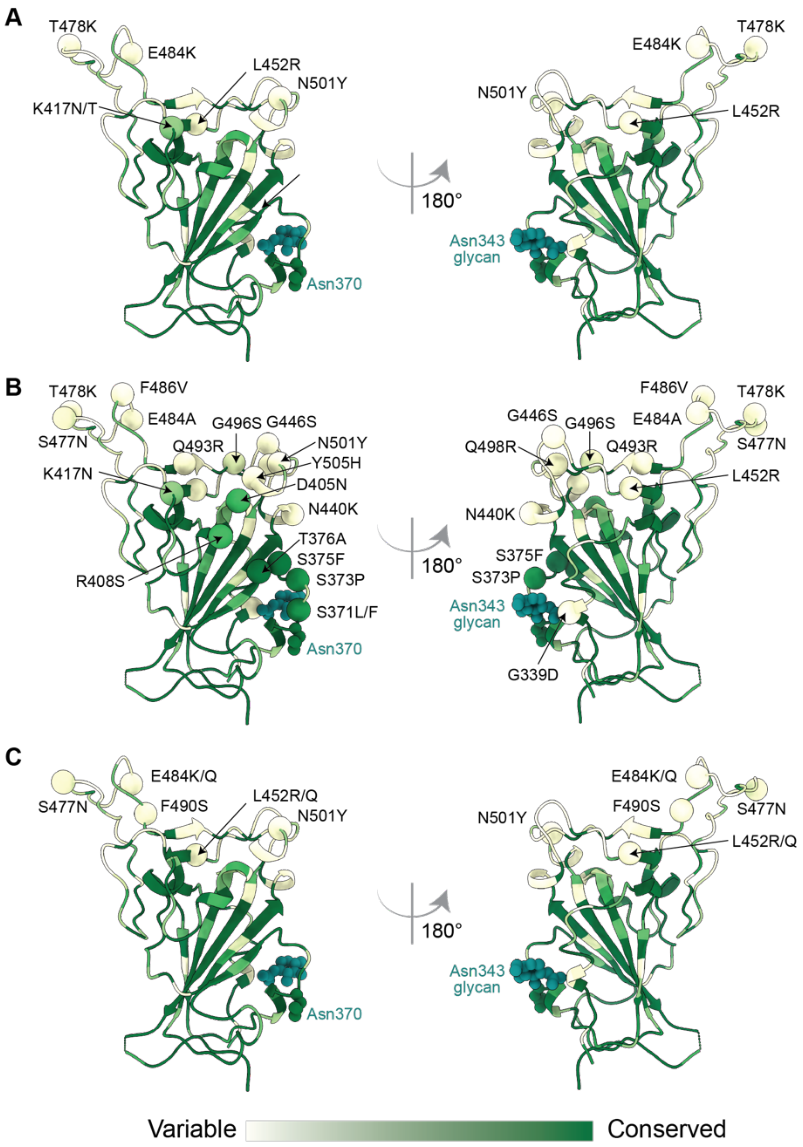

RBD VOC and VOI substitutions. Related to Figure 1. Locations of RBD substitutions in VOCs and VOIs (https://viralzone.expasy.org/9556) are shown as spheres colored according to a variability gradient (bottom) on the WA1 RBD structure (PDB 7BZ5). The N-linked glycan at position 343 of SARS-CoV-2 RBD is shown as teal spheres, and a potential N-linked glycosylation site at position 370 (SARS-CoV-2 numbering) that is found in sarbecovirus RBDs but not in the SARS-CoV-2 RBD is also shown in spheres. (**A**) SARS-CoV-2 Alpha, Beta, Gamma and Delta variants. (**B**) SARS-CoV-2 Omicron BA.1, BA.2, BA.2.12.1, and BA.4/BA.5 variants. (**C**) SARS-CoV-2 VOIs.

A

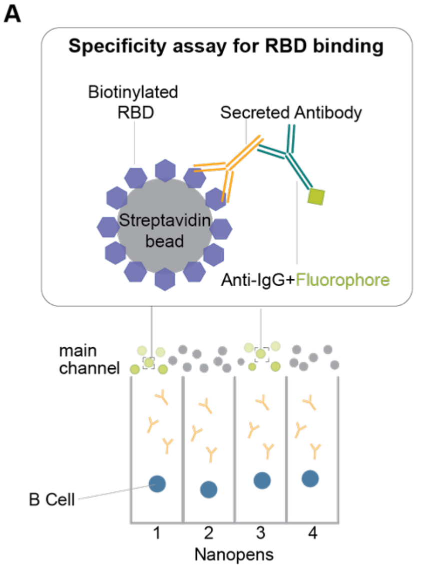

Loading outcome	Homotypic SARS-CoV-2 RBD-mi3 immunized mice	percent of chip	Mosaic-8 RBD-mi3 immunized mice	percent of chip
Pens with single cell	7699	70.0	7747	70.4
Pens with >1 cell	1431	13.0	1948	17.7
Total Pens with cells	9130	83.0	9695	88.1

B

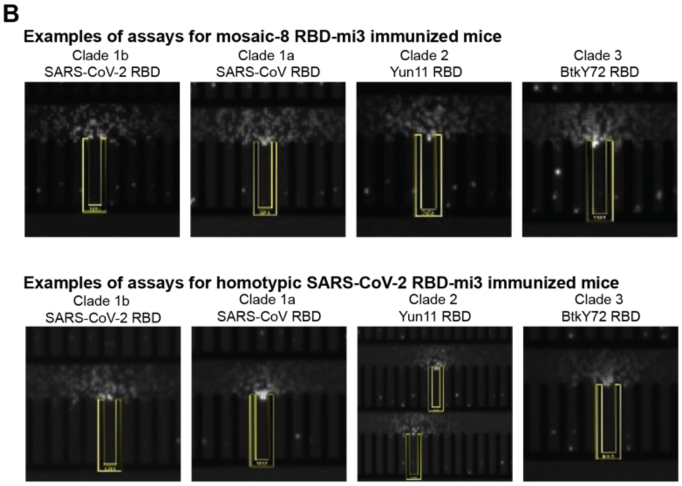

C

Assay outcome		Immunization	
		Mosaic-8 RBD-mi3 immunized mice	Homotypic SARS-CoV-2 RBD-mi3 immunized mice
Number of positive Nanopens for 1 RBD	SARS-CoV-2 RBD	18	130
	SARS-CoV RBD	20	47
	Yun11 RBD	24	39
	BtKY72 RBD	9	36
Number of positive Nanopens for 2 RBDs	SARS-CoV-2 and SARS-CoV RBDs	9	11
	SARS-CoV-2 and Yun11 RBDs	9	12
	SARS-CoV-2 and BtKY72 RBDs	6	9
	SARS-CoV and Yun11 RBDs	7	7
	SARS-CoV and BtKY72 RBDs	7	14
	Yun11 and BtKY72 RBDs	5	17
Number of positive Nanopens for 3 RBDs	All but SARS-CoV RBD	0	3
	All but Yun11 RBD	2	3
	All but BtKY72 RBD	1	1
	All but SARS-CoV-2 RBD	0	2
Number of positive Nanopens for 4 RBDs	SARS-CoV-2, SARS-CoV, Yun11 and BtKY72 RBDs	5	3

Beacon setup. Related to Figure 2 and STAR methods. Individual plasma cells were isolated, cultured, and assayed for RBD binding activities using the Beacon optofluidic system. (**A**) Antigen-specific binding activity was assayed using biotinylated RBDs immobilized on streptavidin-coated beads and loaded into the main channel of the Beacon microfluidic chip, which contained single plasma B cells in individual culture compartments (Nanopens). The specificity of secreted antibodies for the antigen presented in the channel was detected via the local concentration of a fluorescently-labeled secondary above a Nanopen secreting an antigen-specific antibody. (**B**) Examples of detection of secreted antibody binding to different RBDs. The Nanopen(s) from which the secreted antibody signal was emanating over the assay time course is indicated in yellow. (**C**) Summary of cross-reactive antigen-specificity assay results.

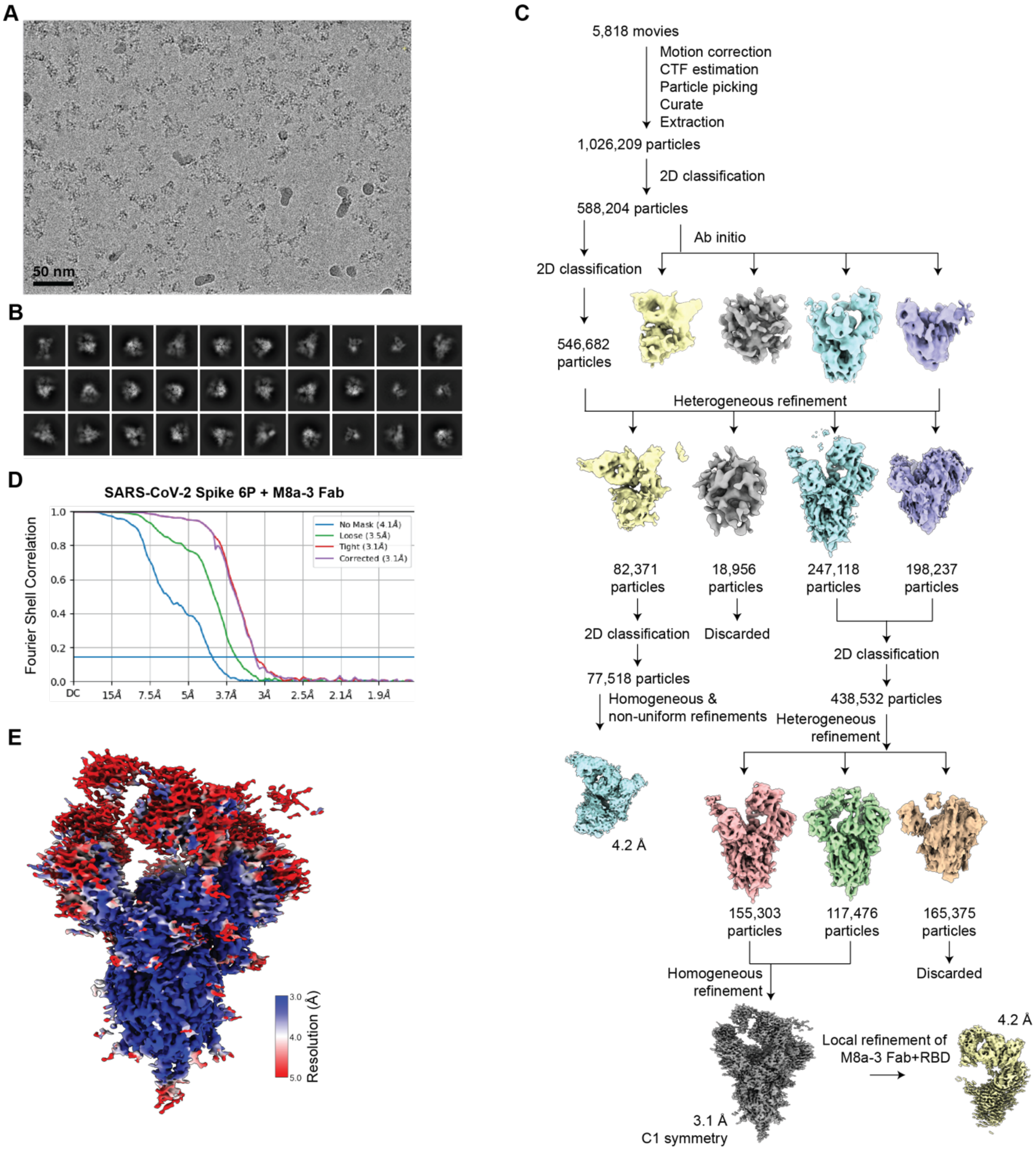

Cryo-EM data processing and validation for of M8a-3 Fab in complex with SARS-CoV-2 WA1 spike. Related to Figure 3. (**A**) Representative micrograph. The scale bar represents 50 nm. (**B**) Representative 2D classes. (**C**) Workflow of single-particle data processing. (**D**) Fourier shell correlation (FSC) plot of the final reconstruction. (**E**) Final reconstruction of M8a-3 Fab in complex with SARS-CoV-2 spike, colored by local resolution.

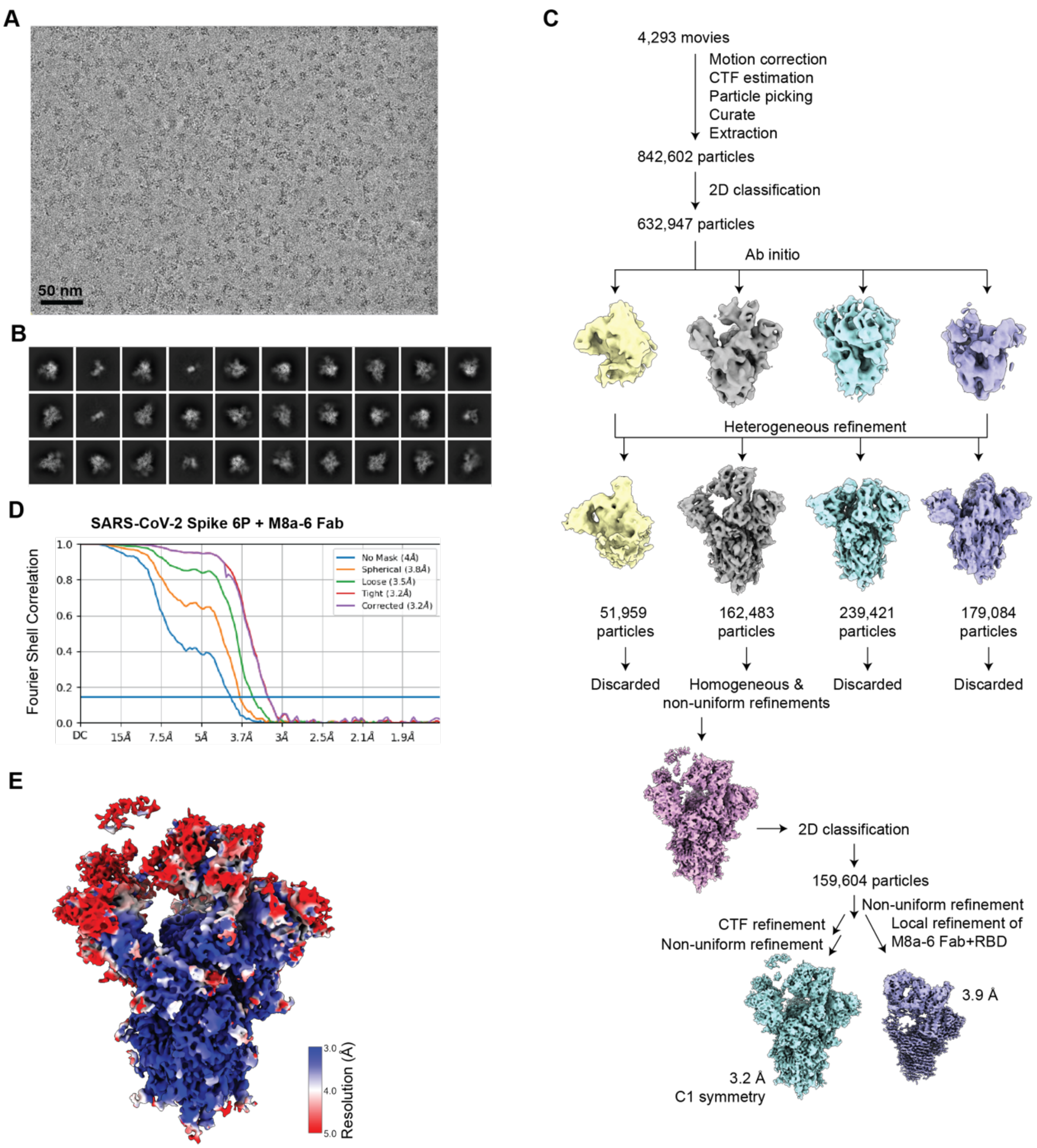

Cryo-EM data processing and validation for M8a-6 Fab in complex with SARS-CoV-2 WA1 spike. **Related to Figure 3.** (**A**) Representative micrograph. The scale bar represents 50 nm. (**B**) Representative 2D classes. (**C**) Workflow of single-particle data processing. (**D**) FSC plot of the final reconstruction. (**E**) Final reconstruction of M8a-6 Fab in complex with SARS-CoV-2 spike, colored by local resolution.

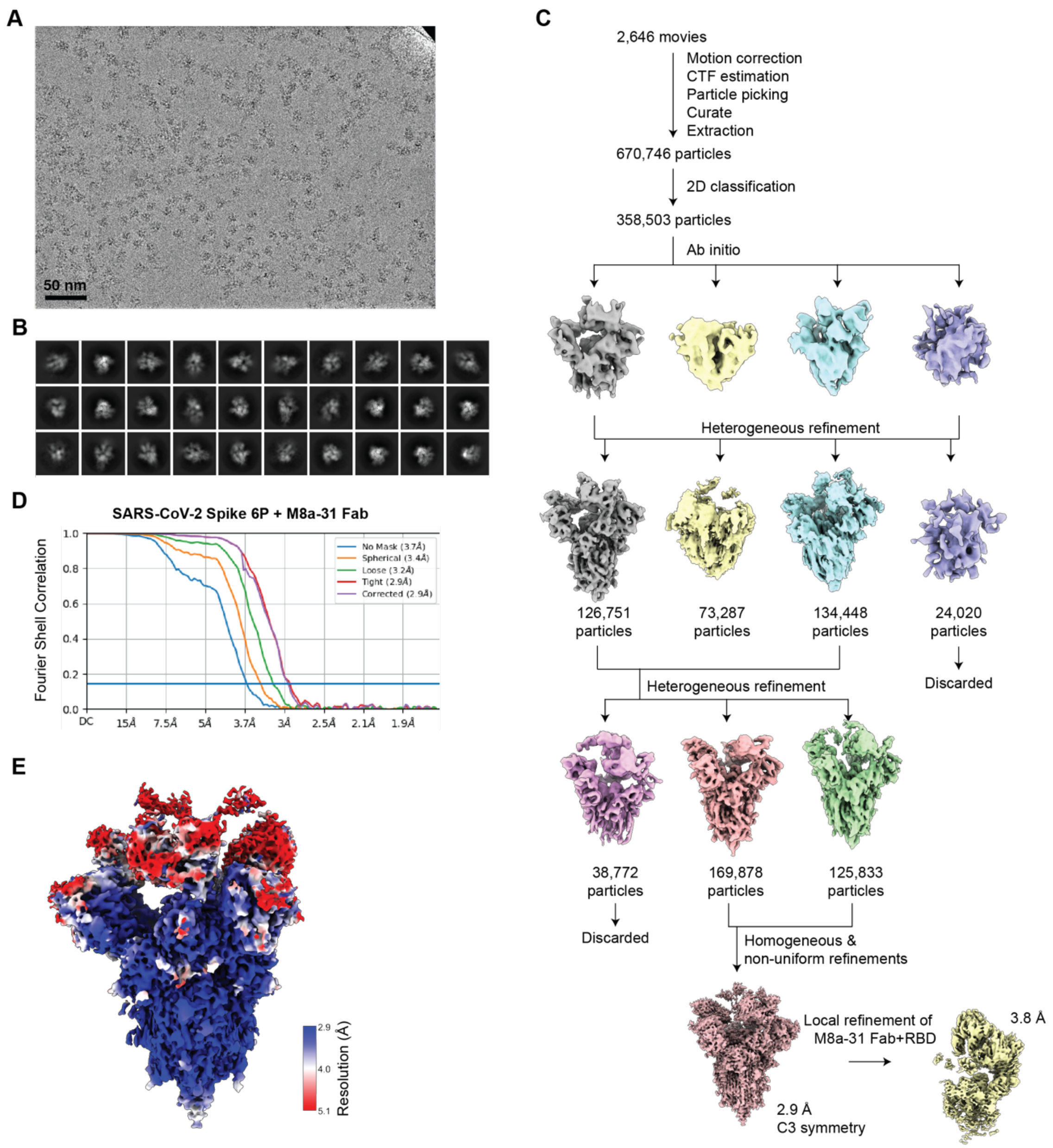

Cryo-EM data processing and validation for of M8a-31 Fab in complex with SARS-CoV-2 WA1 spike. Related to Figure 3. (**A**) Representative micrograph. The scale bar represents 50 nm. (**B**) Representative 2D classes. (**C**) Workflow of single-particle data processing. (**D**) FSC plot of the final reconstruction. (**E**) Final reconstruction of M8a-31 Fab in complex with SARS-CoV-2 spike, colored by local resolution.

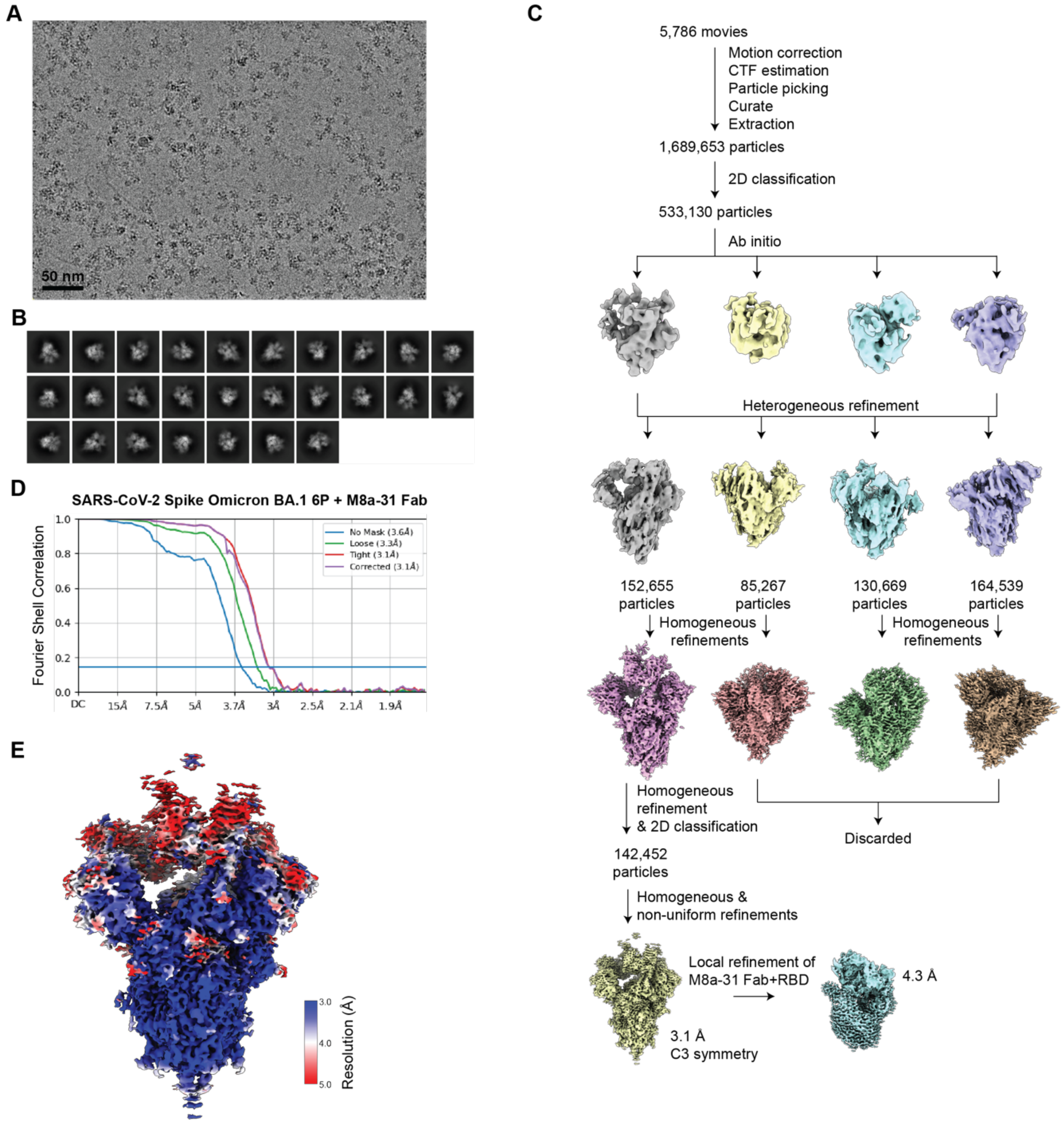

Cryo-EM data processing and validation for of M8a-31 Fab in complex with SARS-CoV-2 Omicron BA.1 spike. Related to Figure 3. (**A**) Representative micrograph. The scale bar represents 50 nm. (**B**) Representative 2D classes. (**C**) Workflow of single-particle data processing. (**D**) FSC plot of the final reconstruction. (**E**) Final reconstruction of M8a-3 Fab in complex with SARS-CoV-2 spike Omicron BA.1, colored by local resolution.

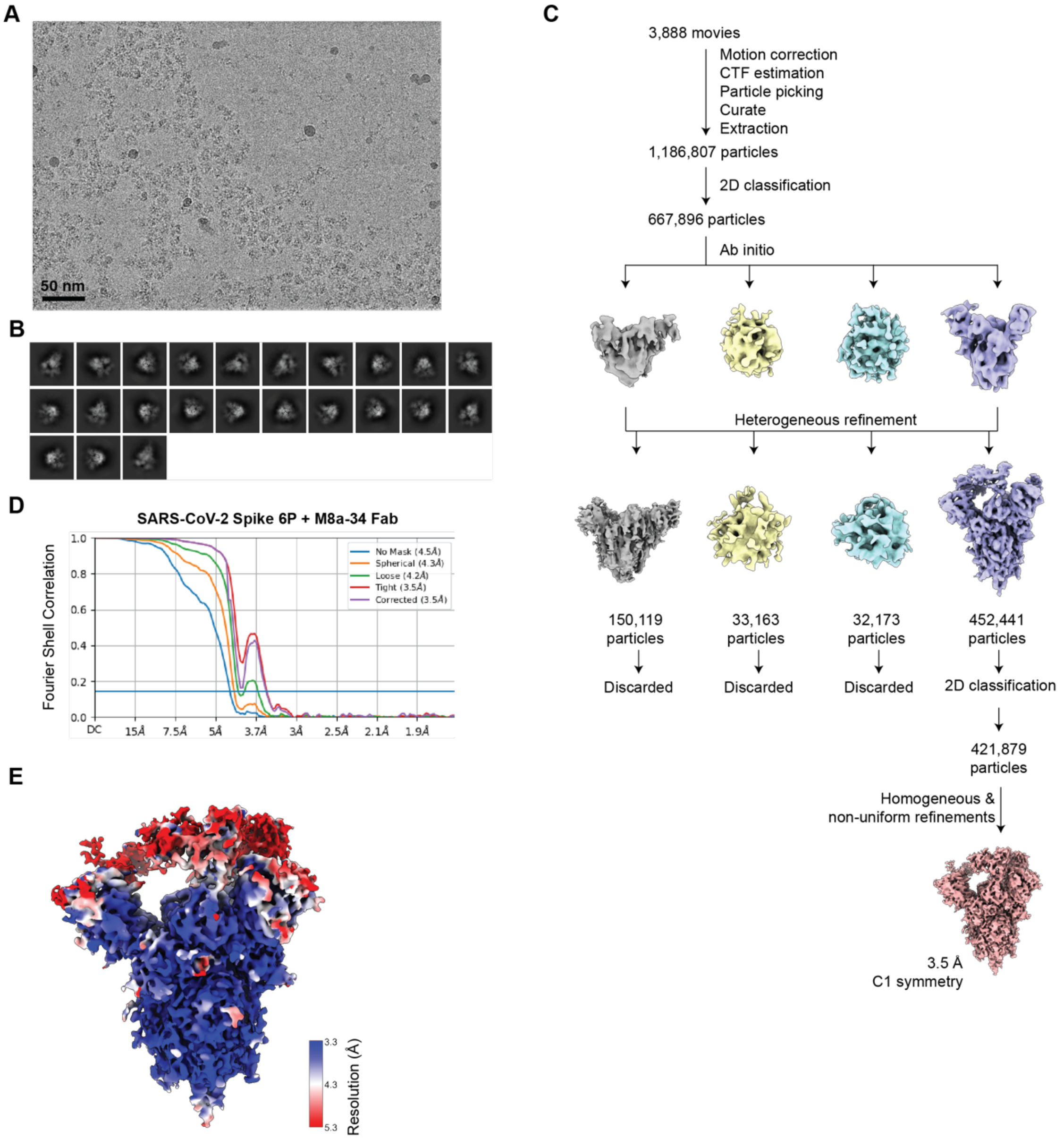

Cryo-EM data processing and validation for of M8a-34 Fab in complex with SARS-CoV-2 WA1 spike. Related to Figure 3. (**A**) Representative micrograph. The scale bar represents 50 nm. (**B**) Representative 2D classes. (**C**) Workflow of single-particle data processing. (**D**) FSC plot of the final reconstruction. (**E**) Final reconstruction of M8a-34 Fab in complex with SARS-CoV-2 spike, colored by local resolution.

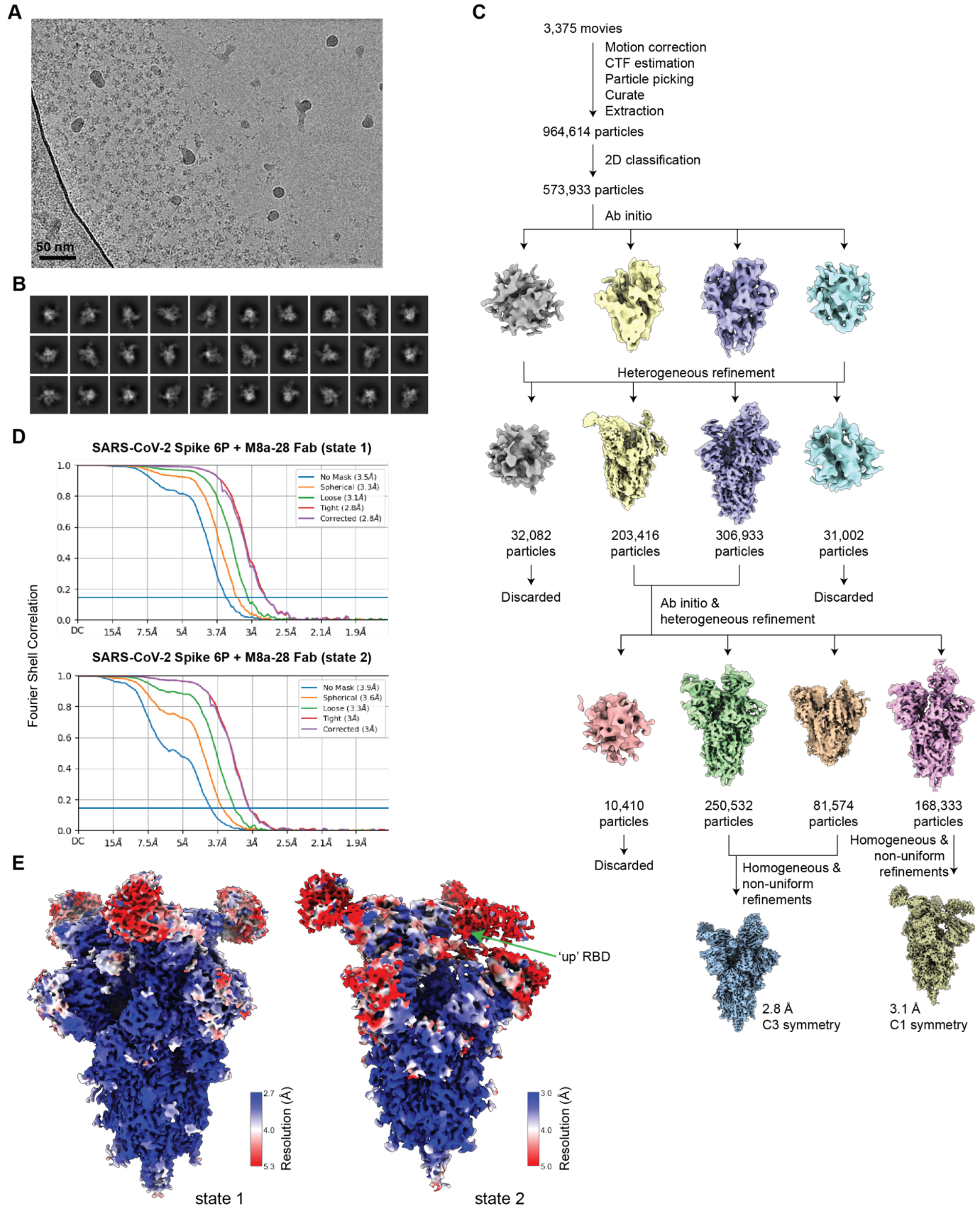

Cryo-EM data processing and validation for M8a-28 Fab in complex with SARS-CoV-2 WA1 spike. **Related to Figure 3.** (**A**) Representative micrograph. The scale bar represents 50 nm. (**B**) Representative 2D classes. (**C**) Workflow of single-particle data processing. (**D**) FSC plot of the final reconstruction. (**E**) Final reconstructions of both states of M8a-28 Fab in complex with SARS-CoV-2 spike, colored by local resolution. An 'up' RBD in state 2 is labeled with an arrow.

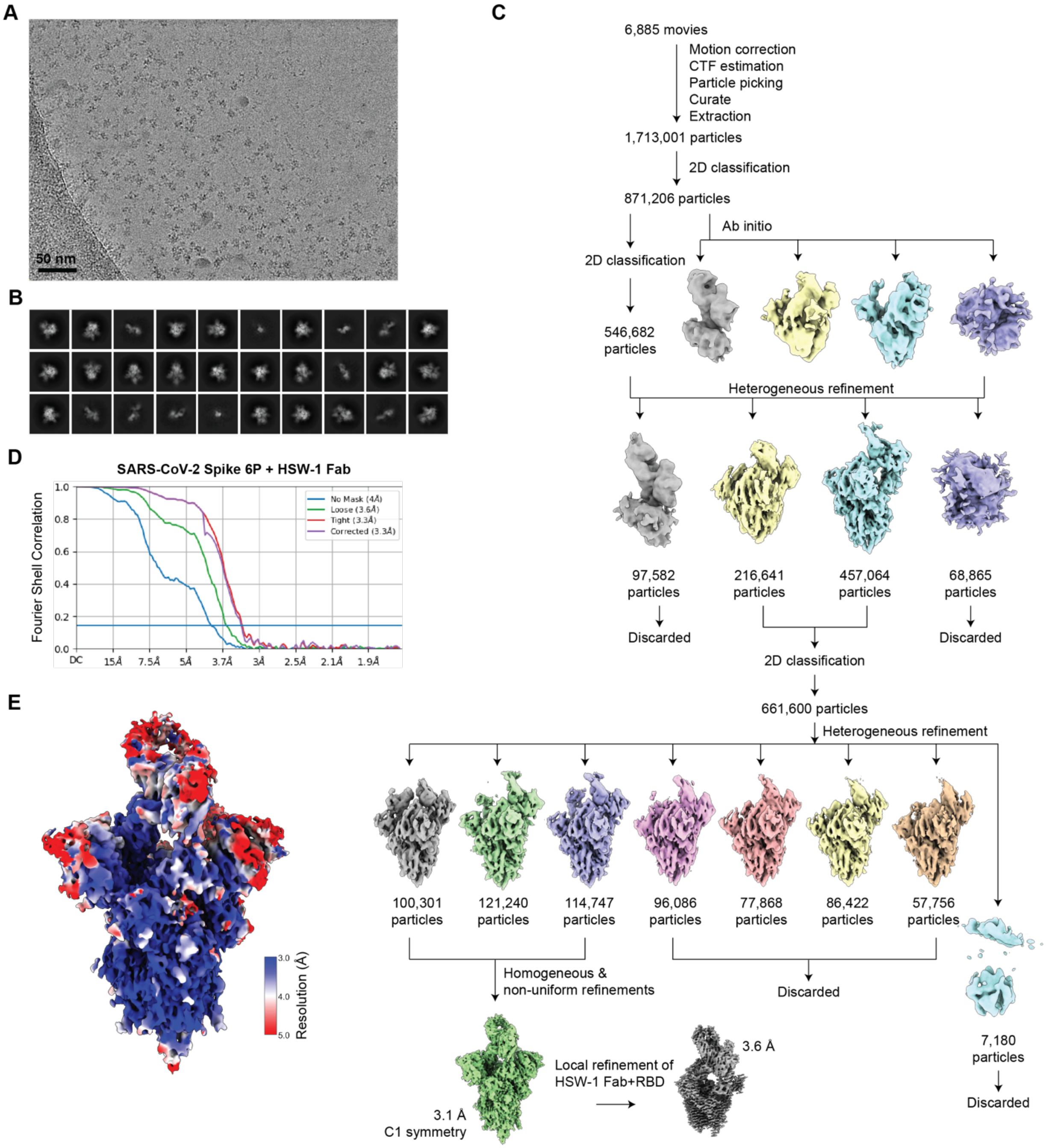

Cryo-EM data processing and validation for of HSW-1 Fab in complex with SARS-CoV-2 WA1 spike. **Related to Figure 4.** (**A**) Representative micrograph. The scale bar represents 50 nm. (**B**) Representative 2D classes. (**C**) Workflow of single-particle data processing. (**D**) FSC plot of the final reconstruction. (**E**) Final reconstruction of HSW-1 Fab in complex with SARS-CoV-2 spike, color by local resolution.

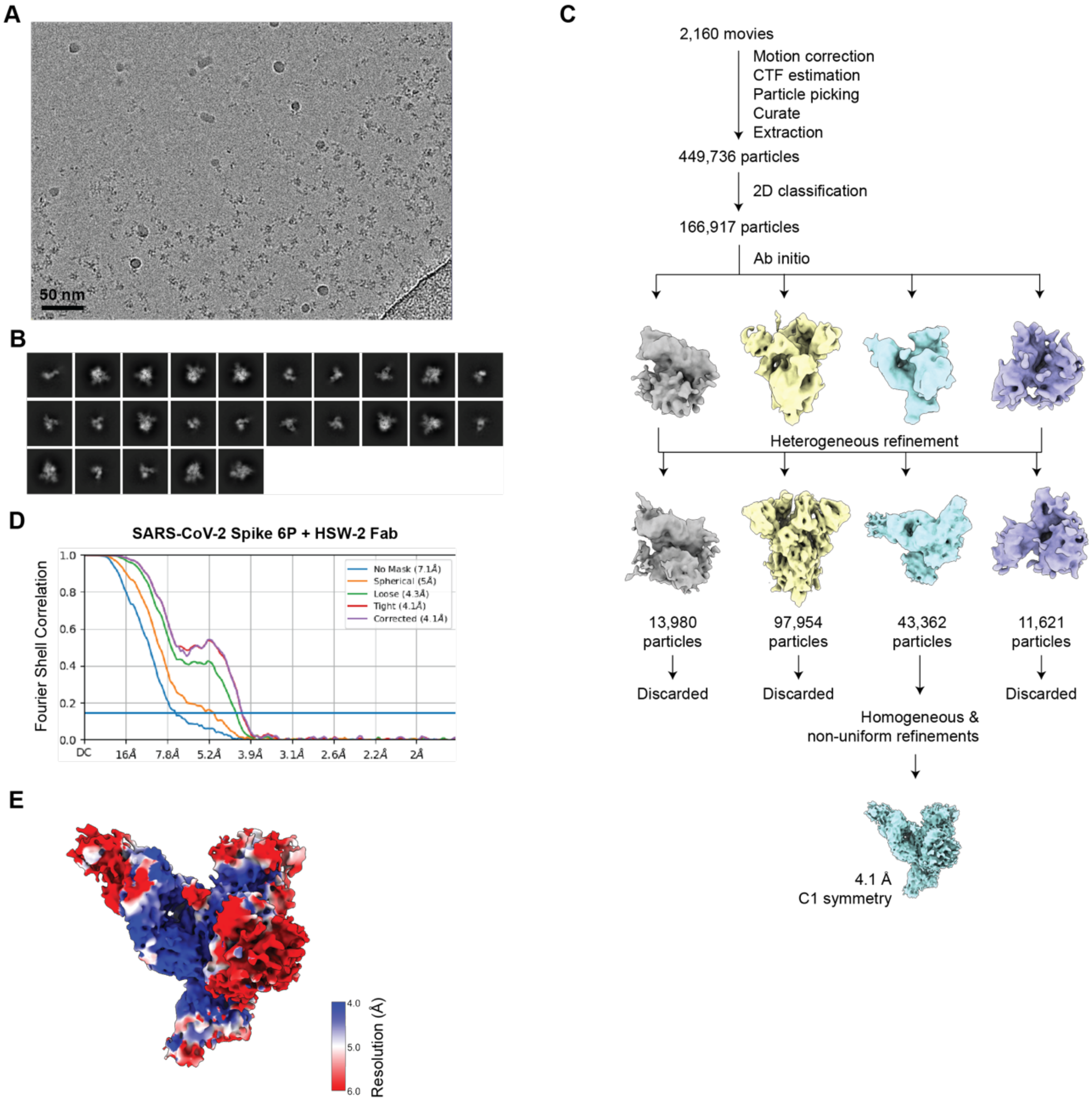

Cryo-EM data processing and validation for of HSW-2 Fab in complex with SARS-CoV-2 WA1 spike. Related to Figure 4. (**A**) Representative micrograph. The scale bar represents 50 nm. (**B**) Representative 2D classes. (**C**) Workflow of single-particle data processing. (**D**) FSC plot of the final reconstruction. (**E**) Final reconstruction of HSW-2 Fab in complex with SARS-CoV-2 spike S1 domain, color by local resolution.

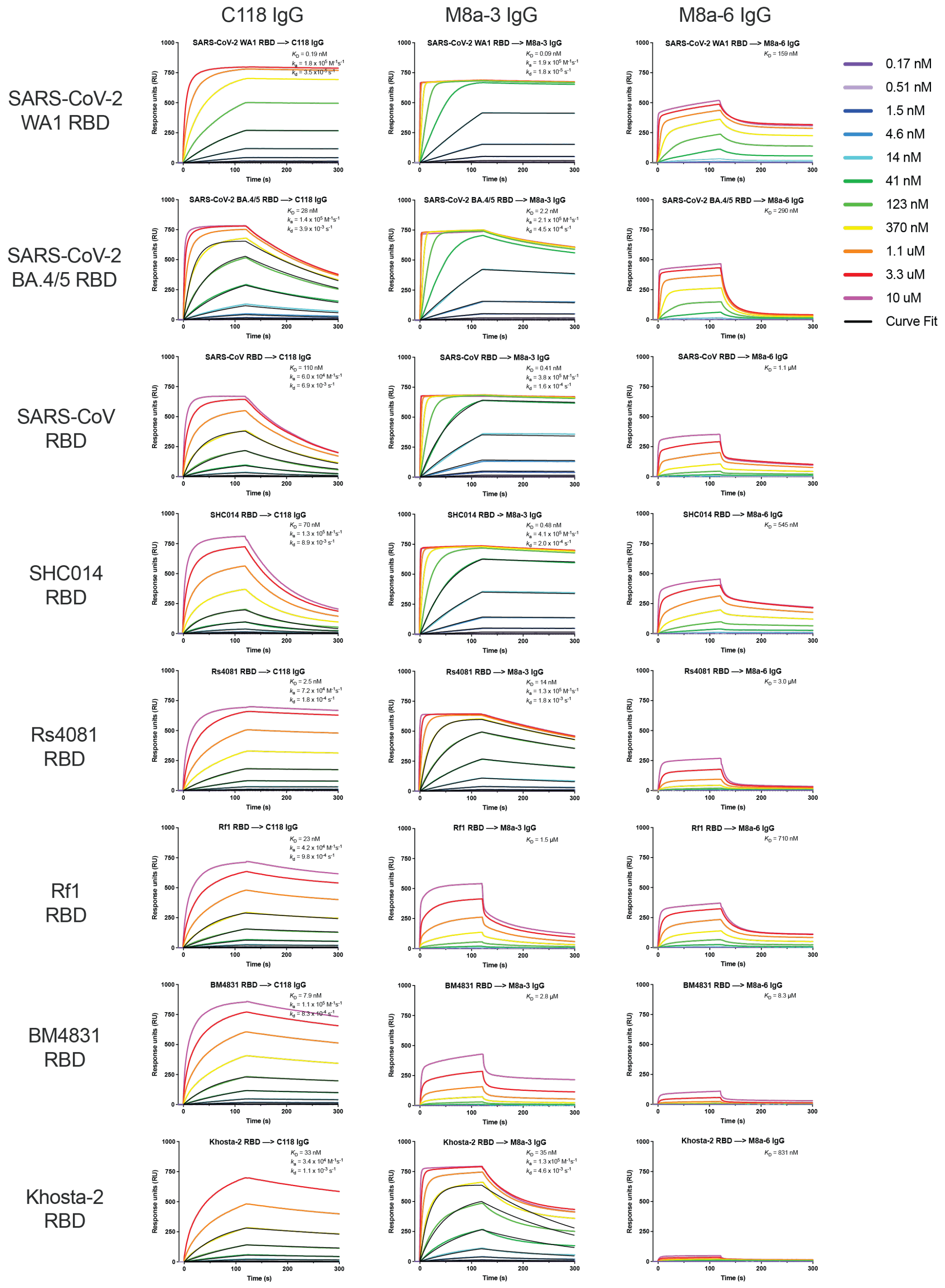

SPR analyses of RBD binding to C118, M8a-3, and M8a-6. Related to Figure 2. IgGs were immobilized on biosensor chips and purified RBDs were injected at the indicated concentrations. Individual binding curves (colored lines) were globally fit to a 1:1 binding model (black) when

possible (most curves for C118 and M8a-3). When indicated in the upper right of the C118 and M8a-3 images, kinetic constants (k_a and k_d) were derived from fitting to a 1:1 binding model, and equilibrium constants (K_D values) were calculated as k_d / k_a. For binding curves that reached or nearly reached equilibrium, K_D values were derived from the midpoints of plots of RBD concentration versus maximum RU that were fit to a 1:1 binding model; these are indicated in the upper right of the M8a-3 and M8a-6 images that are listed without kinetic constants.
