# Supplementary material for: Neutralizing monoclonal antibodies elicited by mosaic RBD nanoparticles bind conserved sarbecovirus epitopes
Source: Immunity. 2022 Dec 13;55(12):2419–2435.e10. doi: 10.1016/j.immuni.2022.10.019 (PMC9606073; doi:10.1016/j.immuni.2022.10.019)
Supplement: Document S2. Article plus supplemental information [file mmc3.pdf]

Article

Immunity

Neutralizing monoclonal antibodies elicited by mosaic RBD nanoparticles bind conserved sarbecovirus epitopes

Graphical abstract

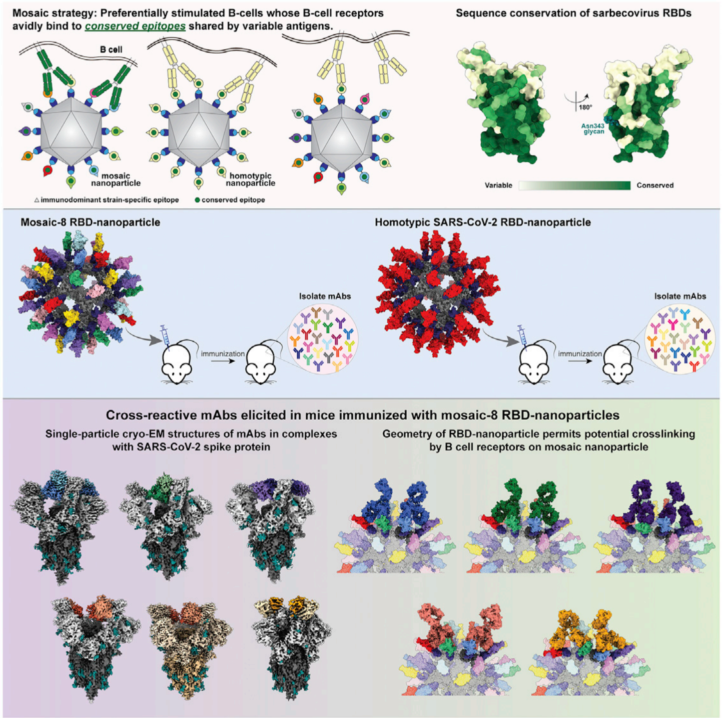

Highlights

- mAbs elicited in mosaic nanoparticle-immunized mice exhibit cross-reactive recognition
- Fab-spike cryo-EM structures show targeting of conserved class 1/4 and 3 RBD epitopes
- Fab-spike structures show increased trimer openness and potential intra-spike binding

Authors

Chengcheng Fan, Alexander A. Cohen, Miso Park, ..., Kathryn E. Malecek, John C. Williams, Pamela J. Bjorkman

Correspondence

bjorkman@caltech.edu

In brief

Sarbecovirus spike receptor-binding domains (RBDs) include conserved and variable epitopes, suggesting that antibodies against conserved regions would protect against future sarbecovirus spillovers and SARS-CoV-2 variants. Fan et al. structurally and functionally characterized monoclonal antibodies elicited by a mosaic-8 RBD-nanoparticle vaccine candidate, demonstrating cross-reactive binding, neutralization, and targeting of desired epitopes.

Fan et al., 2022, Immunity 55, 2419–2435
December 13, 2022 © 2022 The Author(s). Published by Elsevier Inc.
https://doi.org/10.1016/j.immuni.2022.10.019

CellPress

Immunity

CellPress
OPEN ACCESS

Article

Neutralizing monoclonal antibodies elicited by mosaic RBD nanoparticles bind conserved sarbecovirus epitopes

Chengcheng Fan,[1] Alexander A. Cohen,[1] Miso Park,[2] Alfur Fu-Hsin Hung,[2,4] Jennifer R. Keeffe,[1] Priyanthi N.P. Gnanapragasam,[1] Yu E. Lee,[1,5] Han Gao,[1] Leesa M. Kakutani,[1] Ziyan Wu,[1] Harry Kleanthous,[3] Kathryn E. Malecek,[1] John C. Williams,[2] and Pamela J. Bjorkman[1,6,*]

[1]Division of Biology and Biological Engineering, California Institute of Technology, Pasadena, CA 91125, USA
[2]Department of Molecular Medicine, City of Hope, Duarte, CA 91010, USA
[3]Bill and Melinda Gates Foundation, Seattle, WA 98109, USA
[4]Present address: Rakuten Medical Inc., San Diego, CA 92121, USA
[5]Present address: Department of Biology, Stanford University, Stanford, CA 94305, USA
[6]Lead contact
*Correspondence: bjorkman@caltech.edu
https://doi.org/10.1016/j.immuni.2022.10.019

SUMMARY

Increased immune evasion by SARS-CoV-2 variants of concern highlights the need for new therapeutic neutralizing antibodies. Immunization with nanoparticles co-displaying spike receptor-binding domains (RBDs) from eight sarbecoviruses (mosaic-8 RBD-nanoparticles) efficiently elicits cross-reactive polyclonal antibodies against conserved sarbecovirus RBD epitopes. Here, we identified monoclonal antibodies (mAbs) capable of cross-reactive binding and neutralization of animal sarbecoviruses and SARS-CoV-2 variants by screening single mouse B cells secreting IgGs that bind two or more sarbecovirus RBDs. Single-particle cryo-EM structures of antibody-spike complexes, including a Fab-Omicron complex, mapped neutralizing mAbs to conserved class 1/4 RBD epitopes. Structural analyses revealed neutralization mechanisms, potentials for intra-spike trimer cross-linking by IgGs, and induced changes in trimer upon Fab binding. In addition, we identified a mAb-resembling Bebtelovimab, an EUA-approved human class 3 anti-RBD mAb. These results support using mosaic RBD-nanoparticle vaccination to generate and identify therapeutic pan-sarbecovirus and pan-variant mAbs.

INTRODUCTION

Spillover of animal SARS-like betacoronaviruses (sarbecoviruses) resulted in two human health emergencies in the past 20 years: the SARS-CoV epidemic in the early 2000s and the current COVID-19 pandemic caused by SARS-CoV-2. Large coronavirus reservoirs in bats are predictive of future cross-species transmission,[1–3] necessitating a vaccine that could protect against emerging coronaviruses. In addition, SARS-CoV-2 variants of concern (VOCs) have been discovered throughout the current pandemic, designated as such due to increased transmissibility and/or resistance to neutralizing antibodies.[4–7] In the case of Omicron VOCs, a large number of substitutions in the SARS-CoV-2 spike protein receptor-binding domain (RBD), and detectable cross-variant neutralization,[8] results in reduced efficacies of vaccines and therapeutic monoclonal antibodies (mAbs).[5,9]

Comparison of the variability of RBDs across sarbecoviruses and within SARS-CoV-2 variants suggest that vaccines and mAbs targeting the more conserved neutralizing antibody epitopes (class 4 and class 1/4; nomenclature from Barnes et al.[10] and Jette et al.[11] could protect against future zoonotic spillovers and SARS-CoV-2 VOCs. By contrast, antibodies targeting the less conserved class 1 and class 2 RBD epitopes that directly overlap with the binding footprint for human angiotensin-converting enzyme 2 (ACE2) receptor, the SARS-CoV-2 host receptor, recognize a portion of the RBD that exhibits sequence variability between sarbecoviruses,[10] which is also where VOC and variant of interest (VOI) substitutions accumulate. Class 3 RBD epitopes are more conserved than class 1 and class 2 epitopes but exhibit some variation across sarbecoviruses, suggesting the potential for continued variability among SARS-CoV-2 VOCs.[10]

Here, we investigated the RBD epitopes of mAbs isolated from mosaic RBD- and homotypic RBD-immunized mice to characterize the antibody response to RBD nanoparticles. Binding and neutralization results, together with cryoelectron microscopy (cryo-EM) structures of antibody Fab-spike trimer complexes, suggested that the mosaic RBD-nanoparticle vaccine approach works as designed to target conserved epitopes and

This is an open access article under the CC BY license (http://creativecommons.org/licenses/by/4.0/).

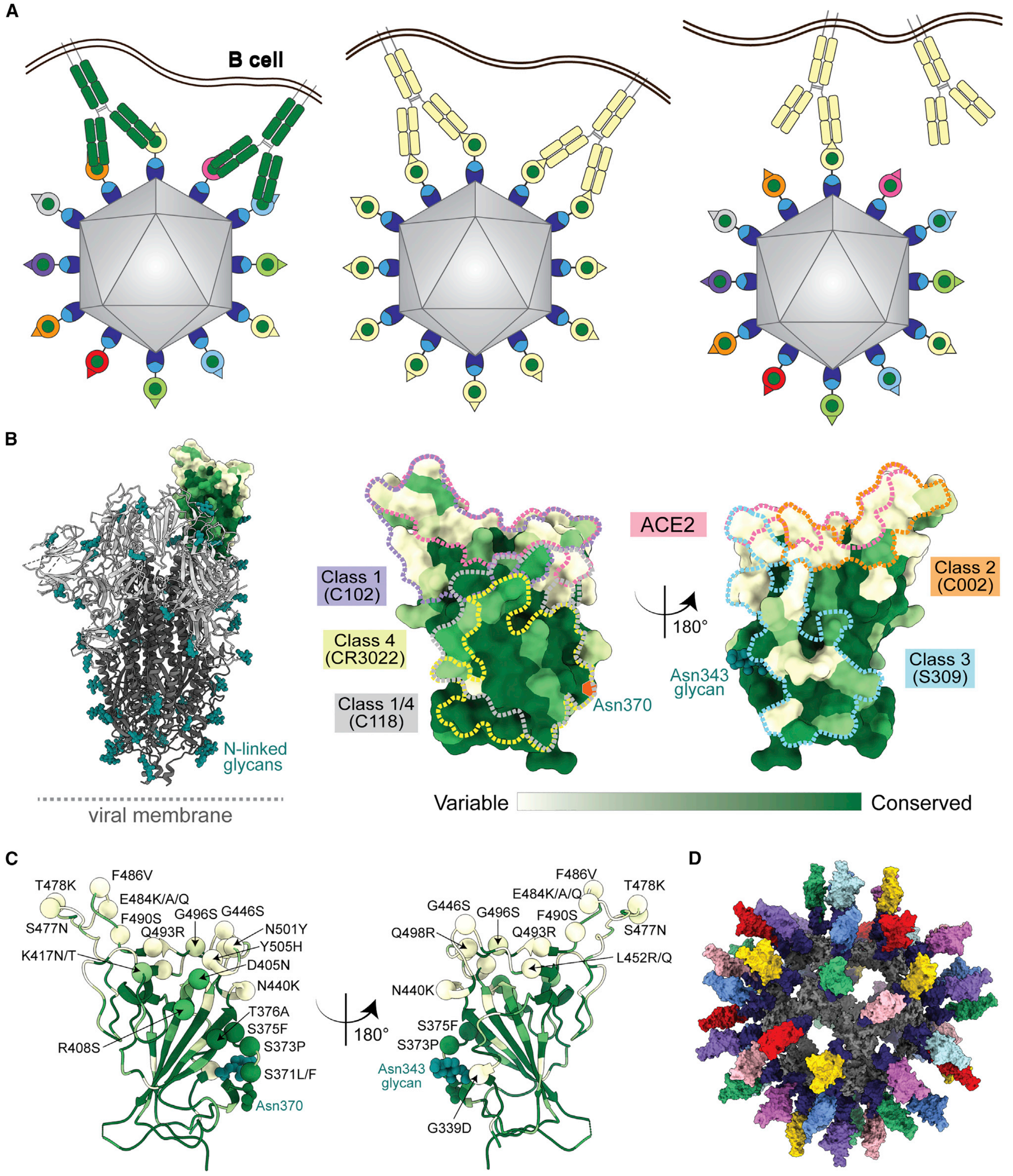

Figure 1. Utilizing antibody avidity effects suggests a strategy to target antibodies to conserved regions of sarbecovirus RBDs

(A) Hypothesis for preferential stimulation of B cells with cross-reactive BCRs by mosaic (left) versus homotypic (right) RBD nanoparticles. Left: green cross-reactive BCRs can crosslink between a conserved epitope (green circles) on adjacent RBDs in a mosaic RBD nanoparticle to enhance binding to a more occluded, but conserved, epitope through avidity effects. Middle: yellow BCRs recognizing an accessible strain-specific epitope (yellow triangle) can crosslink between adjacent RBDs on a homotypic nanoparticle to enhance binding through avidity effects. Right: yellow BCRs against a strain-specific orange epitope cannot crosslink between adjacent RBDs on a mosaic RBD nanoparticle that presents different versions of the epitope (colored triangles).

(legend continued on next page)

could be used both for more broadly protective vaccines and as a method to produce therapeutic neutralizing mAbs that would not be affected by Omicron or future SARS-CoV-2 VOC substitutions.

RESULTS

The majority of mosaic-8-elicited mouse mAbs identified as binding two or more RBDs are cross neutralizing

The hypothesis behind enhanced elicitation of cross-reactive antibodies by mosaic RBD-nanoparticles is that B cell receptors (BCRs) recognizing conserved RBD epitopes are stimulated to produce cross-reactive Abs through bivalent binding of BCRs to adjacent RBDs, which would rarely occur when RBDs are arranged randomly on a nanoparticle (Figure 1A).[12,13] By contrast, homotypic RBD-nanoparticles are predicted to stimulate BCRs against immunodominant strain-specific epitopes presented on all RBDs (Figure 1A). The more conserved class 4 and class 1/4 epitopes (Figure 1B) targeted by polyclonal antibodies in mosaic-8 RBD-nanoparticle antisera are unlikely to vary in SARS-CoV-2 VOCs (Figure 1C; Data S1) because they contact other portions of the spike trimer, unlike class 1 and 2 RBD epitope regions targeted by homotypic SARS-CoV-2 RBD-nanoparticle antisera that are not involved in contacts with non-RBD portions of spike (Figure 1B).[12]

We produced and characterized nanoparticles presenting randomly arranged RBDs from SARS-CoV-2 WA1 and seven animal sarbecoviruses (Pang17, RaTG13, WIV1, SHC014, Rs4081, RmYN02, and Rf1) (mosaic-8 RBD-mi3) and nanoparticles presenting only SARS-CoV-2 WA1 RBDs (homotypic SARS-CoV-2 RBD-mi3)[15] (Figures 1D and S1). Mice were primed and boosted with either mosaic-8 or homotypic SARS-CoV-2 RBD-nanoparticles in AddaVax adjuvant. We used a Berkeley Lights Beacon Optofluidic system to screen a subset of B cells for binding to one or more labeled RBDs (Data S1). B cells secreting IgGs binding at least one RBD were exported, and the variable domains of heavy- and light-chain genes were sequenced and subcloned into expression vectors containing genes encoding human IgG C_H1-C_H2-C_H3 domains, human C_H1, or human C_L domains. From 39 exported cells, we isolated genes for 15 RBD-binding mAbs (Table S1) that were expressed as IgGs and Fabs. The 15 unique IgG sequences included 13 derived from mosaic-8 immunized mice and identified as binding to $\geq$2 (six mAbs) or to one (seven mAbs) labeled RBDs and two derived from homotypic RBD-nanoparticle immunized mice and identified as binding to $\geq$2 RBDs (Figure 2A; Table S1). Two mAbs from mosaic-8 immunized mice were excluded from analyses after showing no detectable binding to purified RBDs (Table S1).

We first evaluated binding of the 13 purified IgGs to RBDs from SARS-CoV-2 variants and other sarbecoviruses using enzyme-linked immunosorbent assays (ELISAs). RBDs were included from sarbecoviruses clades 1a, 1b, 2, and 3 clades (as defined in Starr et al.[16]) (Figure 2A). We compared the mAb binding profiles with four human anti-RBD IgGs with known epitopes: C118, a cross-reactive class 1/4 mAb from a COVID-19 donor;[11,17] S309 (Sotrovimab), a cross-reactive class 3 mAb from a SARS-CoV-infected donor;[18] and mAbs from COVID-19 donors that bind to more variable RBD epitopes overlapping with the ACE2 receptor-binding footprint:[17] C102 (class 1) and C144 (class 2) (Figure 2A). Of the seven murine mAbs identified as secreting IgGs that bound to >1 RBD (Figure 2A), five were isolated from mosaic-8 RBD-nanoparticle-immunized mice (M8a prefixes) and two were from homotypic RBD-nanoparticle-immunized mice (HSW prefixes). These seven mAbs showed binding to SARS-CoV-2 spike trimer and SARS-CoV-2 RBDs that were not represented on the nanoparticle (Beta, Delta, and Omicrons BA.1, BA.2, BA.2.12.1, and BA.4/BA.5), the WA1 variant included in the mosaic-8 RBD nanoparticles and cross-reactive binding to animal sarbecovirus RBDs (Figure 2A). The half-maximal effective concentrations (EC_{50} values) for binding of these mAbs to most of the RBDs ranged from 1 to 10,000 ng/mL (Figure 2A). By comparison, six mAbs that bound only one RBD during screening recognized a smaller subset of RBDs, and none bound to SARS-CoV-2 spike (Figure 2A).

The five M8a IgGs and two HSW IgGs that showed cross-reactive RBD binding during screening and by ELISA shared amino acid sequence identities of ~50%–90% in their V_H and V_L domains (Figures S2A and S2B). They also had varied lengths for their complementarity-determining regions 3 (CDR3s), which are often critical in antigen recognition:[19] the mAb CDR3s ranged from 9 to 16 residues for the heavy-chain CDR3 (CDRH3) and all were 9 residues for the light-chain CDR3 (CDRL3) (Figure S2C), compared with 11 (IgH) and 9 (Igκ) for average C57Bl/6 mouse antibody CDR3s.[20] The CDRH1, CDRH2, and CDRL2 regions were the same lengths across the seven mAbs, whereas the CDRL1 ranged from 6 to 12 residues (Figure S2). M8a-34 and HSW-1 both had long CDRH3s (16 residues), and M8a-31 had the shortest CDRH3 (9 residues). By contrast, M8a-31 had the longest CDRL1 (12 residues) compared with M8a-3, M8a-6, M8a-28, and HSW-2, which all included six-residue CDRL1s (Figure S2C). M8a-3 and M8a-6, related by high sequence identities (87.6% for V_H and 89.7% for V_L) (Figure S2B) and the shared V gene segments (IgH V1-69 and Igκ V6-25) (Figure S2A; Table S1), both contained 14-residue CDRH3s and six-residue CDRL1s (Figure S2C). However, M8a-3 showed a broader RBD binding profile by ELISA, such that it bound all RBDs evaluated except for the clade 2 Rf1 and clade 3 BM4831 RBDs,

(B) Left: structure of SARS-CoV-2 spike (PDB: 6VYB) with one RBD in an "up" position. Right: sequence conservation of 16 sarbecovirus RBDs (Figure S1) calculated by the ConSurf Database[14] plotted on a surface representation of the RBD structure (PDB: 7BZ5). Class 1, 2, 3, 4, and 1/4 epitopes are outlined in different colored dots using information from structures of the representative monoclonal antibodies bound to RBD or spike trimer (C102: PDB: 7K8M; C002: PDB: 7K8T, S309: PDB: 7JX3; CR3022: PDB: 7LOP; and C118: PDB: 7RKV).
(C) RBD mutations of 15 SARS-CoV-2 VOCs and VOIs (https://viralzone.expasy.org/9556) plotted onto the RBD structure (PDB: 7BZ5) as spheres that are colored according to the variability gradient in (A). The N-linked glycan at position 343 of SARS-CoV-2 RBD is shown as teal spheres, and a potential N-linked glycosylation site at position 370 (SARS-CoV-2 numbering) found in some sarbecovirus RBDs but not SARS-CoV-2 RBD is indicated by an orange hexagon.
(D) Structural model of mosaic-8 nanoparticle formed by SpyCatcher-mi3 and eight SpyTagged RBDs made using coordinates of an RBD (PDB: 7SC1), mi3 (PDB: 7B3Y), and SpyCatcher (PDB: 4MLI).
See also Figure S1 and Data S1.

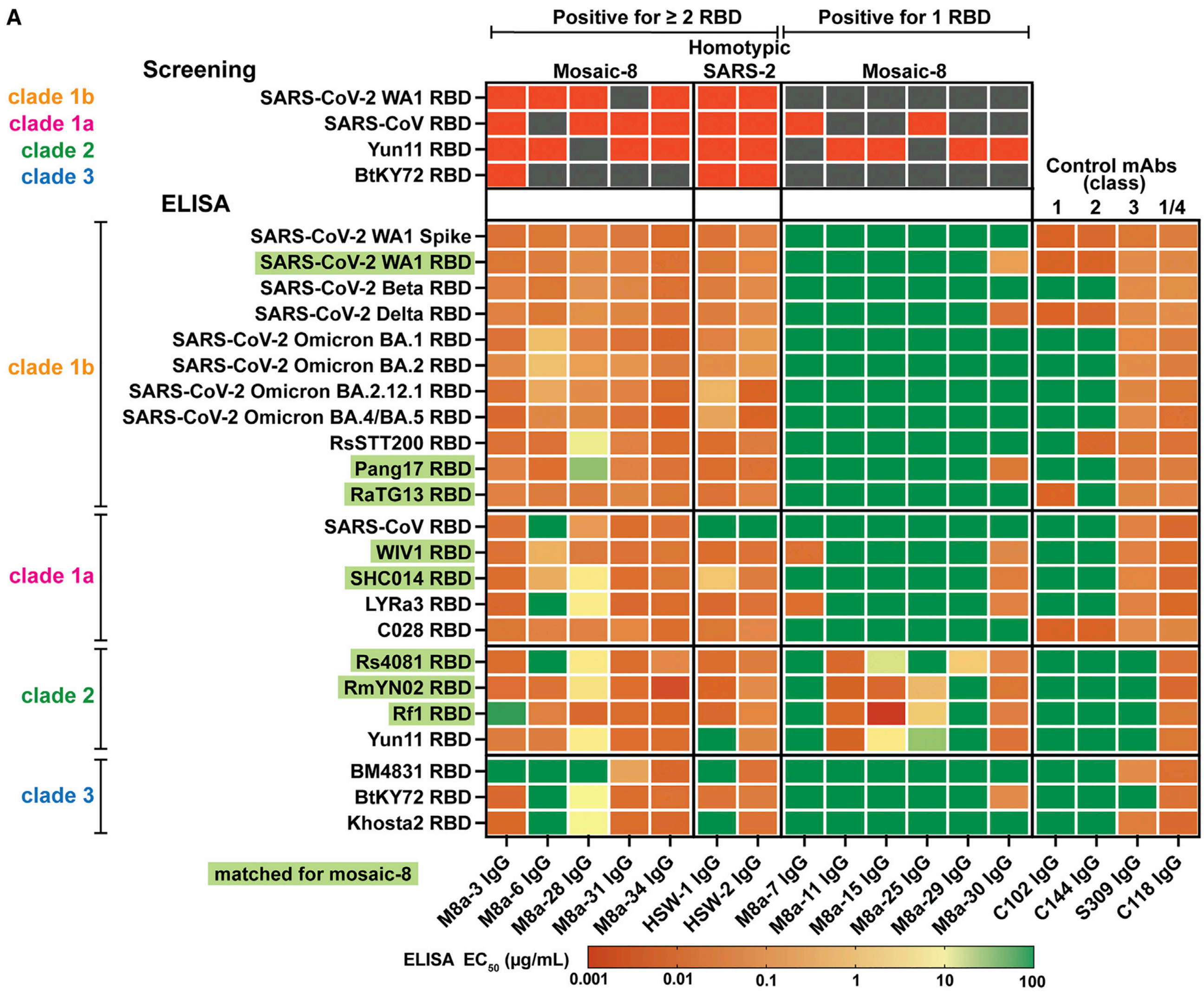

B

IC50 (μg/mL)	Mosaic-8					Homotypic	
	M8a-3	M8a-6	M8a-28	M8a-31	M8a-34	HSW-1	HSW-2
SARS-CoV-2 WA1 D614G	0.18	>100	0.001	5.5	1.5	28	34
SARS-CoV-2 Beta	0.25	>100	0.057	6.3	1.9	31	>50
SARS-CoV-2 Delta	0.13	33	0.051	5.7	0.71	11	37
SARS-CoV-2 Omicron BA.1	1.3	>100	0.011	18	>100	17	>100
SARS-CoV-2 Omicron BA.2	3.8	>100	0.020	6.0	21	17	>100
SARS-CoV-2 Omicron BA.2.12.1	2.4	>100	0.021	3.2	12	12	>100
SARS-CoV-2 Omicron BA.4/5	1.7	>100	0.026	2.2	9.6	7.6	>100
SARS-CoV	0.029	29	7.4	0.99	0.052	>50	>50
WIV1	0.014	21	13	0.79	0.58	8.0	>50
SHC014	0.005	17	>100	0.30	0.002	>50	>50
BtKY72	0.005	8.2	7.8	0.020	0.006	0.20	10
Khosta2/SARS-CoV chimera	9.1	64.5	>100	0.33	0.12	>100	>100
LYRa3/SARS-CoV chimera	0.076	>100	69	35	0.17	15.4	>100

IC50 (μg/mL): < 0.01 | 0.01 - 0.1 | 0.1 - 1 | 1 - 10 | 10 - 100

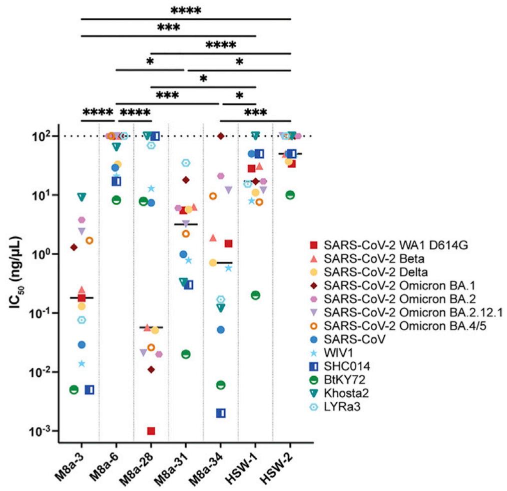

Figure 2. A subset of mAbs elicited in mosaic-8 and homotypic SARS-CoV-2 RBD nanoparticle-immunized mice show cross-reactive binding and neutralization properties

(A) Top four rows: RBDs used for screening of single B cells. Red indicates binding; dark gray indicates no binding. Remaining rows: ELISA EC_{50} values for mouse mAb binding to sarbecovirus RBDs from different clades. RBDs included on the mosaic-8 RBD-nanoparticles are shaded in green. EC_{50} values were derived from

(legend continued on next page)

whereas M8a-6 did not bind detectably to any of the three clade 3 RBDs or to three of the clade 1a and clade 2 RBDs (Figure 2A). M8a-28 showed weak binding to some non-SARS-2 RBDs of clade 1b (RsSTT200 and Pang17), clade 1a (SHC014 and LYRa3), and clade 2 (Rs4081, RmYN02, and Yun11) and weak or no binding to RBDs of clade 3 (weak for BtKY72 and Khosta-2, and no binding to BM4831 RBD of clade 3 (Figure 2A). In contrast, HSW-2 showed binding to RBDs from all clades except SARS-CoV from clade 1a (Figure 2A). M8a-31 and M8a-34 recognized all RBDs in the ELISA panel (Figure 2A). Although M8a-34 and HSW-1 shared a sequence identity of 75.3% for V_H and 88.3% for V_L with the same light-chain IgκV3-5 V gene segment (Figure S2A; Table S1), and both had 16-residue CDRH3s and 10-residue CDRL1s (Figures S2B and S2C), HSW-1 was not as broadly cross-reactive by ELISA (Figure 2A).

We next measured neutralization potencies using a pseudovirus neutralization assay[21] against sarbecoviruses known to use human ACE2 receptor for target cell entry (Figure 2B). M8a-3 was the most consistently potent, exhibiting low half-maximal inhibitory concentrations (IC_{50} values) against all pseudoviruses evaluated (Figure 2B). Despite sharing high sequence identity, the same V gene segments, and similar CDR characteristics with M8a-3 (Figure S2), M8a-6 showed no neutralizing activity except weak activity against BtKY72. A less related mAb, M8a-28, was a potent neutralizer, but only against SARS-CoV-2 variants. M8a-31 and M8a-34 were less potent against SARS-CoV-2 variants but were more broadly cross-reactive, correlating with ELISA profiles (Figures 2A and 2B). By contrast to the five M8a mAbs, HSW-1 and HSW-2 showed overall weaker neutralizing potencies, with 13 of 26 assays showing no neutralizing activity and most of the remaining showing IC_{50} values >10 μg/mL (Figure 2B).

To identify RBD epitopes, we assessed potential competition with proteins that bind to known RBD epitopes, using the four human anti-RBD mAbs used as controls for ELISAs (Figure 2A) plus other potential competitor or control mAbs: C022 (class 1/4),[11,17] CR3022 (class 4),[22] COVA1-16,[23] C135 (class 3), C110 (class 3), C105 (class 1),[17] and a soluble human ACE2-Fc construct.[11] The ELISA revealed the expected competition for the characterized human mAbs, validating its use for mapping RBD epitopes. Three of the five m8a mAbs (M8a-3, M8a-31, and M8a-34) mapped to class 1/4 or class 4 epitopes, M8a-28 mapped to the class 3 RBD region, and Ma-6 did not compete with any of the labeled anti-RBD IgGs (Figure S2D). The identification of a class 3 RBD epitope for M8a-28 rationalized its potent neutralization of SARS-CoV-2 variants and limited neutralization of animal sarbecoviruses (Figure 2B). The class 1/4 RBD epitope identification explained the lower neutralizing potency of M8a-3, M8a-31, and M8a-34, since this class of anti-RBD mAb tends to show less potent neutralization but broader sarbecovirus cross-reactivity, than other classes due to the more occluded nature of the class 1/4 epitope.[11,12,24] Of the two HSW mAbs, HSW-1 showed no detectable competition, and HSW-2 competed with CR3022, a class 4 anti-RBD mAb. These results demonstrated that most of the mAbs identified during Beacon screening mapped to the more conserved class 1/4, 4, and 3 RBD epitopes.

Cryo-EM structures of Fab-spike trimer complexes reveal cross-reactive recognition and rationalize neutralization results

To deduce recognition and neutralization mechanisms, we used single-particle cryo-EM to solve structures of Fabs from the seven cross-reactive mAbs complexed with a SARS-CoV-2 6P spike trimer[25] (Figures 3, 4A, and 5; Table S2; Data S1). Each of the five M8a Fabs were bound to the SARS-CoV-2 WA1 spike, and the M8a-31 Fab was also complexed with the Omicron BA.1 spike (Figures 3A–3F; Table S2; Data S1). We observed one Fab bound to each of the three "up" RBDs, except for the M8a-28-spike structure in which all three RBDs were "down" (Figure 3C) and the M8a-6-spike structure, which showed only one well-resolved Fab per trimer.

A 3.1 Å resolution M8a-3 Fab-spike complex structure revealed Fab V_H-V_L interactions with "up" RBDs using all six CDRs along with residues within the light-chain framework region 2 and 3 (FWRL2 and FWRL3) (Figures 3A, 4B, and S3A; Data S1). Consistent with the competition ELISA results (Figure S2D), comparison of the M8a-3 Fab-RBD interaction with previously characterized representative anti-RBD antibodies in different structural classes[10,11] showed overlap with the class 1 and class 4 RBD epitopes (Figure S3A) and a binding footprint adjacent to that of ACE2 receptor (Figures 3A and 4A). This was similar to the human mAb C118, a class 1/4 anti-RBD antibody that blocks ACE2 binding without substantially overlapping with the ACE2 receptor binding footprint[11] and competes with M8a-3 for RBD binding (Figure S2D). The M8a-3-spike structure recognized a largely conserved region of the RBD (Figure 4B), consistent with ELISA and neutralization results where M8a-3 neutralized and/or bound to most of the sarbecoviruses and the SARS-CoV-2 variants tested (Figure 2).

A 3.2 Å spike trimer structure complexed with the related, but mostly non-neutralizing, M8a-6 mAb showed three "up" RBDs but only one well-resolved Fab (Figures 3B and S3B; Data S1). The M8a-6 Fab shared a similar RBD epitope and approach angle as M8a-3 (Figures 3A and 4A; Figure S3B), interacting with the RBD using all six CDRs plus framework regions FWRH2, FWRL2, and FWRL3 (Figure 4C). Furthermore, M8a-6 also recognized a similar epitope as C118[11] and M8a-3, involving mostly conserved RBD residues (Figures 4C and S3B). Despite sharing high sequence identity and similar binding epitopes on SARS-CoV-2 RBD with M8a-3, M8a-6 was

ELISAs conducted with duplicate samples at least twice (for first seven mAbs) or once (for remaining mAbs). The same EC_{50} values are presented for M8a-11 and M8a-26, which shared the same protein sequences.

(B) Left: neutralization potencies (IC_{50} values) of mAbs against SARS-CoV-2 variants and indicated sarbecoviruses. IC_{50}s are reported from neutralization assays that were conducted using duplicate samples at least twice except for a single assay for M8a-28 against Omicron BA.1. Right: median IC_{50} values. Significant differences calculated using Tukey's multiple comparison test between mAbs linked by horizontal lines are indicated by asterisks: *$p < 0.05$, **$p < 0.01$, ***$p < 0.001$, ****$p < 0.0001$. Medians are represented by black lines for IC_{50} values of each mAb.

See also Figure S2, Table S1, and Data S1.

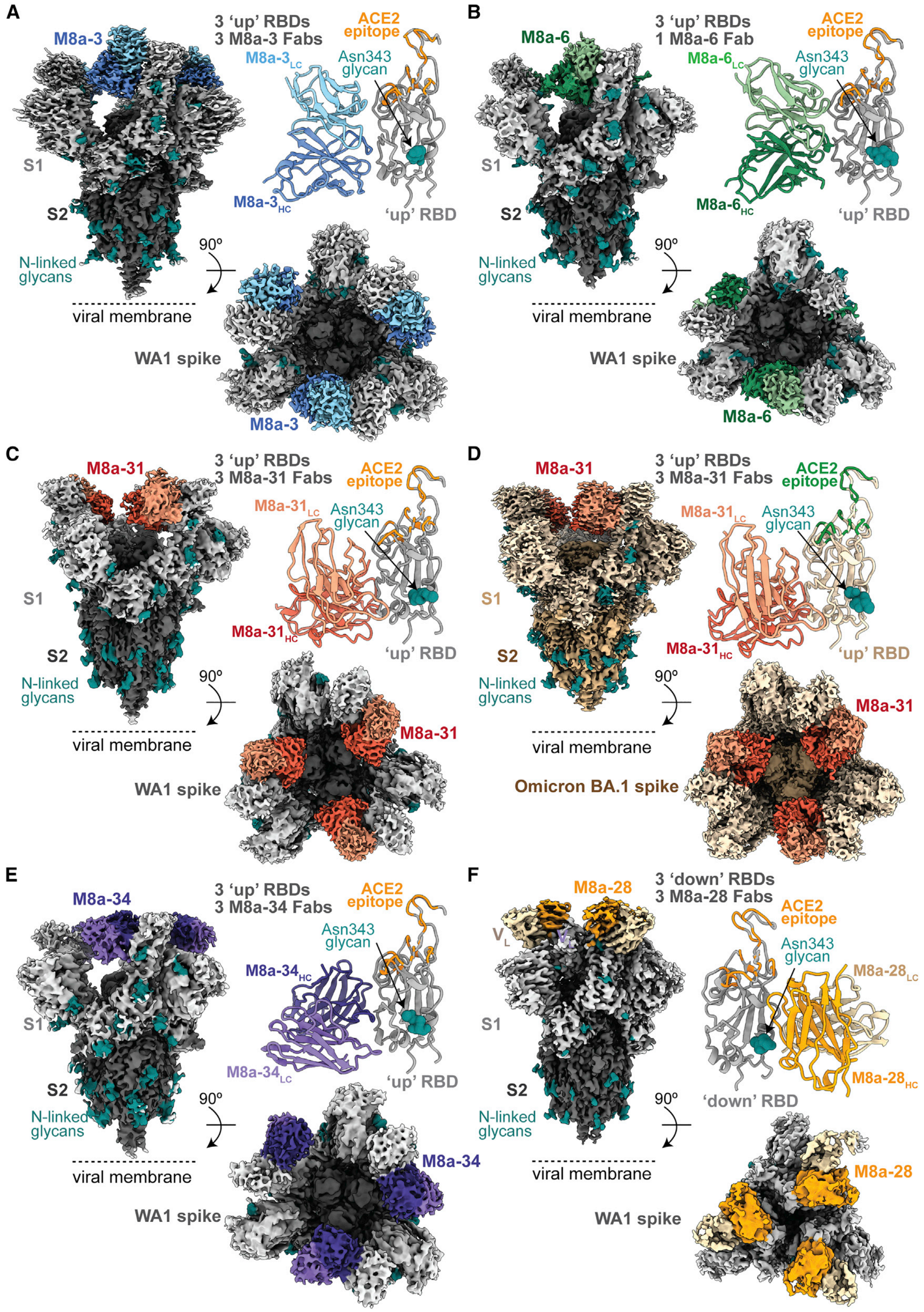

(legend on next page)

CellPress
OPEN ACCESS

non-neutralizing against SARS-CoV-2 and only weakly neutralizing against BtKY72, whereas M8a-3 neutralized SARS-CoV-2 D614G with a 0.18 μg/mL IC_{50} (Figure 2B). These different neutralization profiles likely result from a weaker interaction of M8a-6 compared with M8a-3 with CoV spikes, as demonstrated by incomplete binding of Fabs in the M8a-6-spike complex cryo-EM structure (Figure 3B) and the lack of competition of M8a-6 IgG with any of the IgGs with known epitopes (Figure S2D). To investigate whether M8a-6 binds more weakly to its RBD epitope than M8a-3, we used surface plasmon resonance (SPR) to examine binding of M8a-3 and M8a-6 compared with C118[11] to a set of eight RBDs (Data S1). Visual inspection of sensorgrams and kinetic and equilibrium constants (when they could be derived by fitting data to a 1:1 binding model) showed weaker RBD binding by M8a-6 than by M8a-3 or C118.

Similar to M8a-3, M8a-31 exhibited cross-reactive binding and neutralization across SARS-CoV-2 variants and other sarbecoviruses (Figure 2) and competed with class 1/4 and class 4 anti-RBD antibodies[11] (Figure S2D). Single-particle cryo-EM structures were determined for M8a-31 Fab bound to SARS-CoV-2 WA1 and to Omicron BA.1 (Figures 3C and 3D; Data S1) spike trimers at resolutions of 2.9 and 3.1 Å, respectively. In both structures, three M8a-31 Fabs interacted with "up" RBDs (Figures 3C, 3D, S3C, and S3D). Despite 15 substitutions in the Omicron BA.1 RBD compared with the WA1 RBD, the M8a-31 epitopes and binding poses in both structures were similar (Figures S3C and S3D) (root mean square deviation [RMSD] of 1.0 Å calculated using 1,267 resolved Cα atoms in each Fab-spike protomer). M8a-31 Fab binding to SARS-CoV-2 WA1 and Omicron BA.1 RBDs was mainly stabilized through interactions with FWRH1, FWRH2, FWRL2, and FWRL3, and all CDRs except for CDRL3 (Figure 4D). The M8a-31 epitope overlapped with class 4 anti-RBD antibodies but was shifted toward the ACE2 receptor binding site compared with CR3022 (class 4) (Figures S3C and S3D), consistent with its competition with the C118 class 1/4 mAb (Figure S2D). Conservation of the M8a-31 epitope (Figure 4D) is consistent with its cross-reactive binding and neutralization properties (Figure 2).

M8a-34 also bound and neutralized most sarbecoviruses across different clades and SARS-CoV-2 variants (Figure 2) and exhibited a similar competition as M8a-3 and M8a-31 (Figure S2D). To map its epitope, we determined a cryo-EM structure of M8a-34 Fab bound to the WA1 spike trimer at 3.5 Å resolution (Figure 3E; Data S1), revealing interactions of three Fabs with three "up" RBDs (Figures 3E and S3E) that were modeled using an M8a-34 Fab-RBD crystal structure (Table S3). M8a-34 Fab interacted with the RBD through all three CDRHs as well as CDRL1 and CDRL3 (Figures 4E and S3G). The M8a-34 epitope was similar to epitopes of other class 1/4 mAbs including M8a-3, M8a-6, and M8a-31, which overlapped with the binding epitopes of CR3022 (class 4) and C118 (class 1/4) (Figures 4A and S3E), again consistent with its binding and neutralizing properties (Figure 2) and competition ELISA results (Figure S2D).

M8a-28, which showed the lowest degree of cross-reactive RBD binding (Figure 2A), mapped to the class 3 epitope instead of the more conserved class 1/4 and class 4 epitopes (Figure S2D), and except for M8a-6, it showed the lowest levels of cross-reactive sarbecovirus neutralization of the five mAbs isolated from mosaic-8 immunized mice (Figure 2B). Single-particle cryo-EM structures of the M8a-28 Fab-spike complex were determined in two conformational states: a 2.8 Å structure with each of three Fabs binding to a "down" RBD (Figure 3F) and a 3.1 Å structure with two Fabs bound to adjacent "down" RBDs and a third Fab at lower occupancy bound to a flexible "up" RBD (Data S1). The Fab-RBD interaction was mediated by all six CDRs, plus FWRH3 and FWRL1 (Figures 4F and S3H). The M8a-28 Fab approached the RBD from the opposite direction compared with Fabs from the other M8a mAbs (Figures 4A and S3F), interacting with more variable RBD regions (Figure 4F) that overlap with the epitope of the S309 (class 3) mAb[18] (Figure S3F). Although M8a-28 potently neutralized SARS-CoV-2 WA1 D614G, Beta, Delta, Omicron BA.1, BA.2, BA.2.12.1, and BA.4/BA.5, it was only weakly neutralizing or non-neutralizing against other sarbecoviruses (Figure 2B), consistent with its epitope spanning more variable RBD residues than epitopes of class 4 and class 1/4 anti-RBD mAbs.[11]

Despite broad recognition of sarbecovirus RBDs (Figure 2A), the HSW mAbs exhibited overall weaker neutralization potencies across the sarbecoviruses tested, with all IC_{50} values >10 μg/mL (Figure 2B). To compare recognition properties with the M8a Fabs, we determined a cryo-EM structure of HSW-1 bound to WA1 spike at 3.1 Å resolution, revealing a single well-ordered Fab bound to a trimer with two "up" RBDs and one "down" RBD (Figures 5A and S4A; Data S1). The bound HSW-1 Fab interacted with two RBDs: one "up" RBD (1° RBD) and the adjacent "down" RBD (2° RBD) (Figures 5A and S4A). Interactions between HSW-1 and the 1° RBD were mediated by FWRH1, CDRH1, CDRH3, CDRL1, CDRL2, CDRL3, and FWRL2 and only by the HSW-1 light chain for the 2° RBD (Figures 5A and 5B). Structural comparisons showed the epitope of HSW-1 overlapped somewhat with the binding epitopes of C118 (class 1/4) and CR3022 (class 4) and included mostly conserved residues (Figure S4A).

We next used cryo-EM to investigate HSW-2-spike interactions, observing two main populations of particles: unliganded intact spike trimers and a Fab-spike S1 domain protomer complex (Data S1). From the latter, we obtained an EM reconstruction at 4.1 Å of HSW-2 Fab bound to the WA1 S1 domain (Figures 5C and S4B) using a crystal structure of an HSW-2 Fab-RBD complex (Table S3) to derive detailed interactions. HSW-2 used its six CDRs plus FWRH2, FWRL1, FWRL2, and FWRL3 to recognize the bottom of the RBD (Figures 5D and 5E), consistent with its competition with CR3022 (class 4)

Figure 3. mAbs isolated from mice immunized with mosaic-8 nanoparticles target epitopes outside of the ACE2 receptor binding footprint
EM densities of single-particle cryo-EM structures of Fab V_H-V_L-spike trimer complexes are shown from the side (upper left), top (lower right), and as cartoon diagrams of the Fab V_H-V_L interaction with the RBD (upper right; RBD residues involved in ACE2 receptor binding are orange for complexes with WA1 spike and green for the complex with Omicron BA.1). Complex structures are shown for (A) M8a-3-WA1, (B) M8a-6-WA1, (C) M8a-31-WA1, (D) M8a-31-Omicron BA.1, (E) M8a-34-WA1, and (F) M8a-28-WA1.
See also Figure S3, Tables S2 and S3, and Data S1.

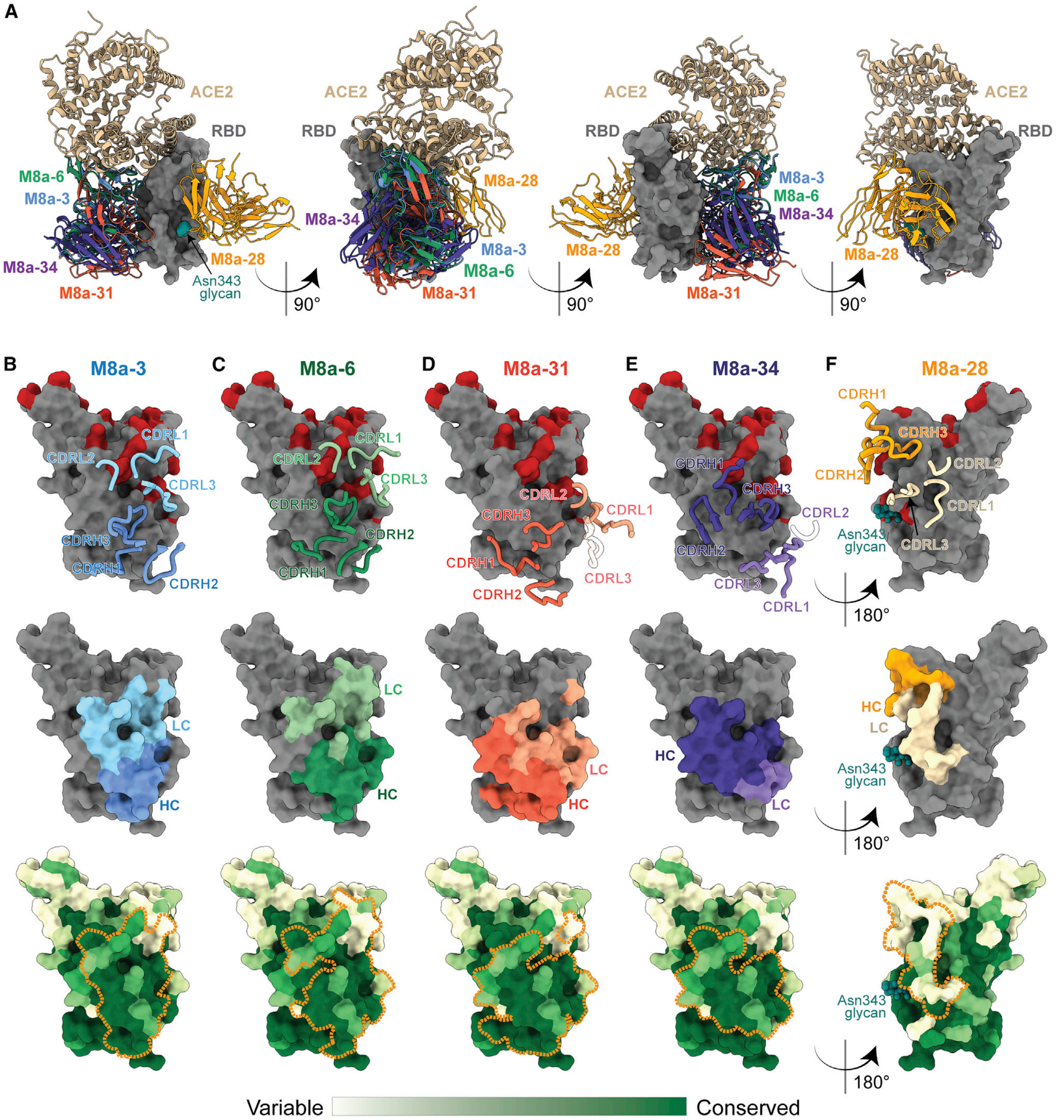

Figure 4. Epitopes of mAbs elicited by mosaic-8 immunization demonstrate targeting of non-class 1/class 2 RBD epitopes

(A) Four views of the RBD surface (dark gray) with overlays of mAb V_H-V_L domains (different colored cartoon representations) from Fab-spike structures. ACE2 receptor (tan cartoon) complexed with RBD (PDB: 6M0J) is shown for comparison.

(B–F) mAb epitopes on RBD surfaces shown with overlaid heavy- and light-chain CDRs (IMGT definitions) (top, CDRs that do not interact with the RBD are shown in transparent cartoons), as colored areas for heavy and light chains (middle) and outlined with orange dotted lines on a sequence conservation surface plot (bottom; calculated using the 16 sarbecovirus RBD sequences shown in Figure S1). The N-linked glycan at RBD position Asn343 is shown as spheres. Omicron BA.1, BA.2, BA.2.12.1, and BA.4/BA.5 substitutions are colored red in the top panels.

(B) M8a-3.

(C) M8a-6.

(D) M8a-31 from complex with WA1 spike.

(E) M8a-34.

(F) M8a-28.

See also Figure S4, Tables S2 and S3, and Data S1.

CellPress
OPEN ACCESS

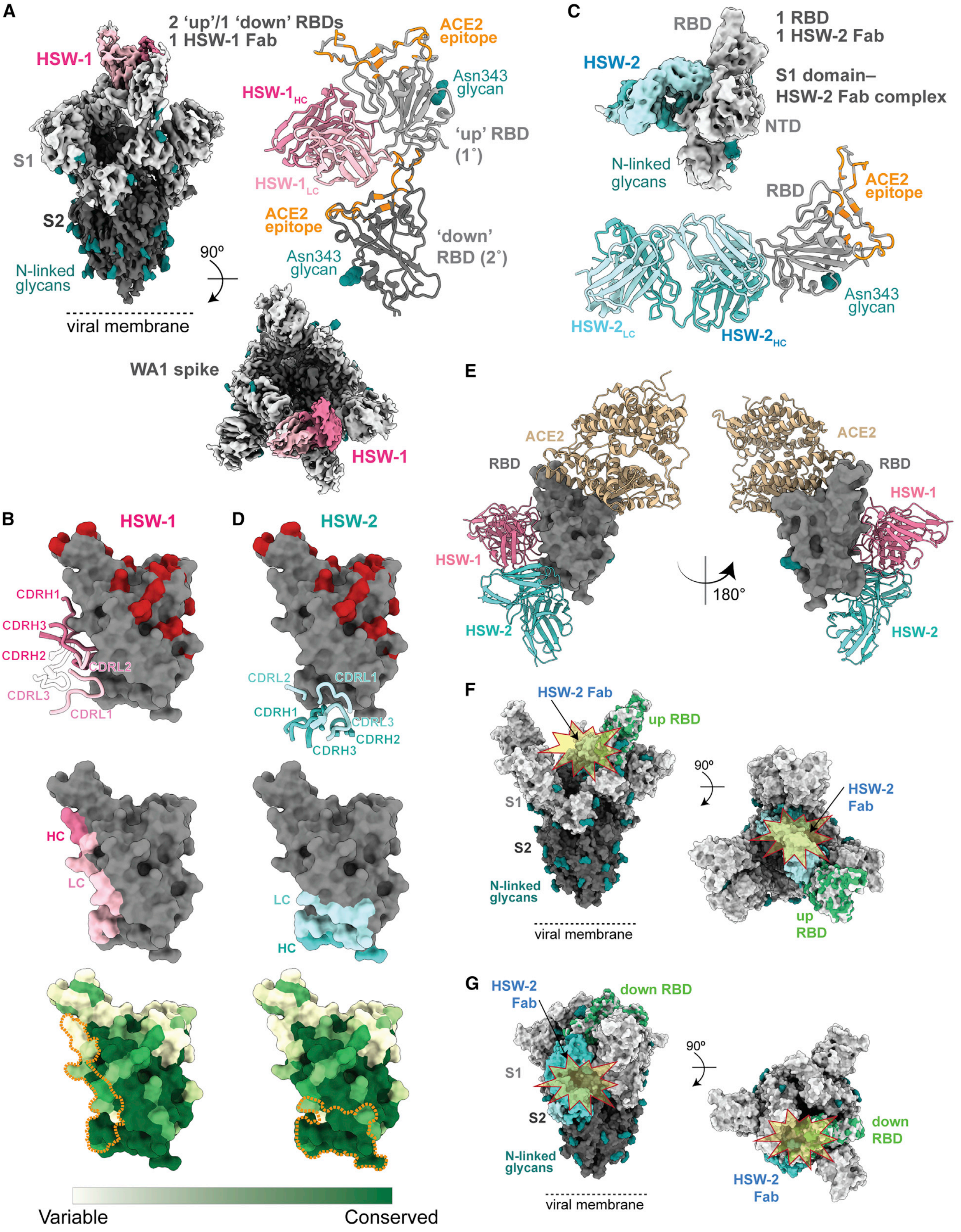

(legend on next page)

(Figure S2D), and although their binding poses differed, the HSW-2 and CR3022 epitopes overlapped (Figure S4B).[22] S1 shedding resulting from mAb binding has been suggested as a possible neutralization mechanism for CR3022 and other class 4 anti-RBD mAbs;[22,26,27] however, HSW-2 was largely non-neutralizing (Figure 2B). To determine accessibility of the HSW-2 epitope in an intact spike trimer, we aligned the RBD portion of the HSW-2 Fab-RBD structure to RBDs from spike structures with all "up" or all "down" RBDs, finding steric clashes in both cases (Figures 5F and 5G). The inability of the HSW-2 Fab to access either "up" or "down" RBDs in an intact spike trimer is consistent with the observation that HSW-2 showed weak or no neutralization activity (Figure 2B) despite binding almost all RBDs evaluated by ELISA (Figure 2A).

In summary, structural studies corroborated the competition assay mapping of the mouse mAb epitopes (Figure S2D) and further revealed details of RBD recognition in the context of spike trimers.

Class 1/4 anti-RBD mAbs induce spike trimer opening and exhibit different potentials for intra-spike crosslinking and susceptibility to mutations

To address potential effects of mAb binding on spike trimer conformation, we compared the Fab-spike structures reported here with other trimer structures. We previously assessed spike openness using measurements of inter-RBD distances between residue 428 Cα atoms in adjacent "up" RBDs, with $\leq$39 Å indicating a typical prefusion spike trimer conformation (Figure 6A) (unliganded, bound to ACE2 or a class 1, 2, or 3 anti-RBD mAb) and increased distances indicating binding of class 4 and class 1/4 anti-RBD mAbs[11] (Figure 6B). In the present study, we found inter-protomer distances of 48–69 Å for trimers bound to M8a-3 (Figure 6C), M8a-6 (Figure 6D), M8a-31 (Figures 6E and 6F), M8a-34 (Figure 6G), and HSW-1 (Figure 6H), consistent with increased openness of class 1/4- and class 4-bound trimers. By contrast, the comparable distance was 31 Å in M8a-28-spikes with all "down" RBDs (Figure 6I), consistent with M8a-28 recognition of the non-occluded class 3 epitope.

To understand how substitutions in VOCs might affect binding of the mAbs for which we had Fab-spike structures, we mapped their binding epitopes compared with Omicron RBD substitutions (Figures 4B–4F, 5B, and 5D). Most of the Omicron substitutions were in the more variable ACE2 receptor binding region (Figures 1A and 4A; Data S1), with fewer substitutions in conserved regions (Figures 1A, 4B–4F, 5B, and 5D; Data S1). Omicron substitutions were mainly at the peripheries of the RBD epitopes of the m8a mAbs isolated from mosaic-8-immunized mice (Figures 4B–4F), and there were no Omicron substitutions within the binding epitopes of the two HSW mAbs isolated from homotypic nanoparticle-immunized mice (Figures 5B and 5D). Despite the Omicron substitutions not greatly affecting RBD binding by the seven mAbs (Figure 2A), some of the class 1/4 M8a mAbs showed somewhat reduced neutralization potencies (Figure 2B).

Although RBD binding correlates with neutralization potencies for polyclonal antisera from RBD-nanoparticle immunized animals,[15] this is true for all mAbs, e.g., CR3022 binds to SARS-CoV-2 RBD but neutralizes only weakly or not at all.[30] One mechanism by which Omicron or other RBD substitutions could indirectly affect neutralization potencies of mAbs without affecting binding to isolated RBDs is by changing the dynamics of the conversion between "up" and "down" RBD conformations on spike trimers. Some classes of anti-RBD mAbs have a strong or absolute preference for binding an "up" versus a "down" RBD, e.g., most class 1 and class 4 anti-RBD mAbs only recognize "up" RBDs.[10] To assess whether the mAbs investigated here recognized "up" and/or "down" RBDs, we evaluated the accessibility of their epitopes on a spike by mapping each binding epitope onto an unliganded trimer structure with one "up" and two "down" RBDs (PDB: 6VYB) (Figure S5) and a trimer with all "up" RBDs (PDB: 7RKV) (Figure S6). The class 4 and 1/4 epitopes of M8a-3, M8a-6, M8a-31, M8a-34, and HSW-1 were buried when RBDs adopted the "down" conformation (Figures S5A–S5D and S5F) but fully exposed in the "up" RBDs (Figures S6A–S6D and S6F). Although the HSW-2 class 4 epitope was buried in "down" RBD conformation (Figure S5G) and could be partially exposed in an "up" RBD conformation (Figure S6G), structure alignments showed that HSW-2 cannot bind "up" or "down" RBDs in the context of a spike trimer (Figures 5F and 5G). By contrast, the class 3 epitope of M8a-28 was exposed in both RBD conformations (Figures S5E and S6E). Likely related to these observations, only the M8a-28-bound trimer structure showed an inter-protomer RBD distance of 31 Å (Figure 6I) equivalent to that of an unliganded trimer

Figure 5. Epitopes of mAbs isolated from mice immunized with homotypic SARS-CoV-2 nanoparticles that target conserved RBD epitopes

(A) EM density of cryo-EM structure of HSW-1 Fab-spike complex shown from the side (upper left), top (lower right), and as a cartoon diagram of the HSW-1 V_H-V_L interaction with two adjacent RBDs (1° and 2°) (upper right). HSW-1 interacts mainly with an "up" RBD (1° RBD, light gray) but also includes V_L interactions with a "down" RBD (2° RBD, dark gray).

(B) HSW-1 epitope on RBD surface shown with overlaid heavy- and light-chain CDRs (IMGT definitions) (top, CDRs that do not interact with the RBD are shown in transparent cartoons), as colored areas for heavy and light chains (middle) and outlined with orange dotted lines on a sequence conservation surface plot (bottom; calculated using the 16 sarbecovirus RBD sequences in Figure S1). Omicron BA.1, BA.2, BA.2.12.1, and BA.4/BA.5 substitutions are colored red in the top panel.

(C) EM density of cryo-EM structure of HSW-2-Fab S1 domain complex (top) and cartoon diagram of the HSW-2 V_H-V_L interaction with the RBD (bottom).

(D) HSW-1 epitope on RBD surface shown with overlaid heavy- and light-chain CDRs (top), as colored areas for heavy and light chains (middle), and outlined with orange dotted lines on a sequence conservation surface plot (bottom; calculated using the 16 sarbecovirus RBD sequences shown in Figure S1). Omicron BA.1, BA.2, BA.2.12.1, and BA.4/BA.5 substitutions are colored red in the top panel.

(E) Two views of RBD surface (dark gray) with overlays of mAb V_H-V_L domains (different colored cartoon representations) from HSW Fab-spike structures and ACE2 (tan cartoon representation from PDB: 6M0J).

(F and G) Superpositions of HSW-2-RBD structure onto the RBD from a spike trimer structure showing that HSW-2 Fab is sterically hindered from binding to either an "up" or "down" RBD on an intact spike due to clashes (starbursts) with the spike S2 domain. (F) HSW-2 Fab-RBD interaction modeled onto an "up" RBD from the M8a-31-spike complex structure. (G) HSW-2 Fab-RBD interaction modeled onto a "down" RBD from the M8a-28-spike complex structure.

See also Figures S5 and S6.

CellPress
OPEN ACCESS

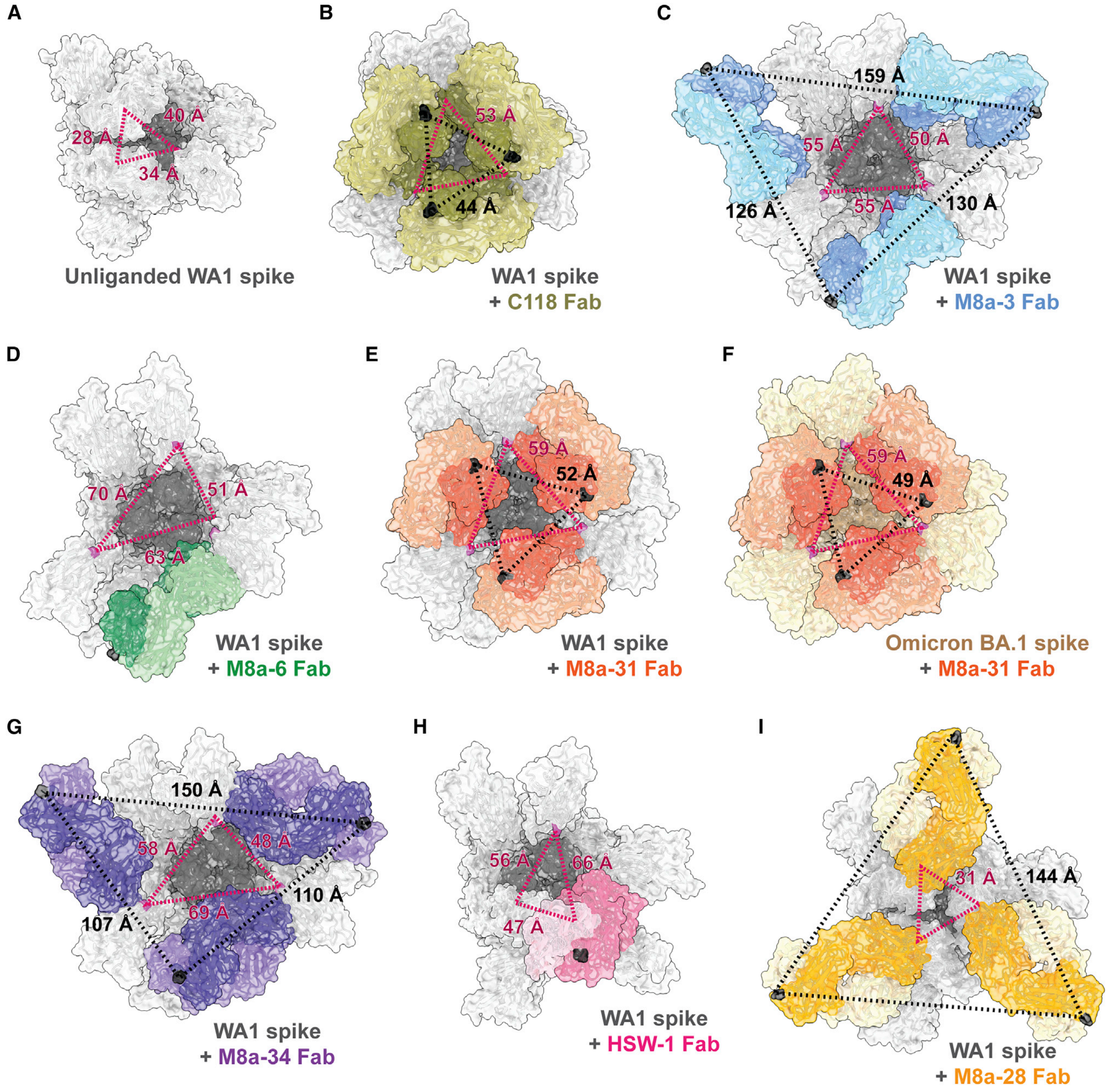

Figure 6. Spike-mAb complex structures show increased trimer openness and the potential for intra-spike IgG cross-linking

Red dotted lines: trimer openness was assessed by measuring distances between the Cα atoms of RBD residue 428 (pink) in each RBD of a spike trimer (top-down views with mAb Fabs shown in colors on a gray spike trimer [WA1] or an orange spike trimer [Omicron BA.1]). Distances of ≤39 Å indicate a typical closed, prefusion spike trimer conformation[10] (A). Binding of class 1/4 anti-RBD antibodies such as C118 and S2X259 result in larger inter-RBD distances indicating a more open trimer conformation: 53 Å for C118 (B) and 43 Å for S2X259 (PDB: 7RA8). Black dotted lines: the potential for intra-spike cross-linking by the two Fabs of a single bound IgG was assessed by measuring distances between the Cα atoms of C-terminal C_H1 residues (black) on adjacent bound Fabs on the RBDs of a spike trimer. Distances <65 Å are considered compatible with the potential for intra-spike cross-linking.[10]

(A) Unliganded spike (PDB: 6VYB): closed prefusion conformation.

(B) C118 Fab-WA1 (PDB: 7RKV): open trimer conformation with potential for intra-spike crosslinking by C118 IgG.

(C) M8a-3 Fab-WA1: open trimer confirmation with no potential for intra-spike crosslinking.

(D) M8a-6 Fab-WA1: open trimer conformation. Black dotted lines between the Cα atoms of C-terminal C_H1 residues are not shown because the reconstruction included only one Fab.

(E) M8a-31 Fab-WA1: open trimer conformation with potential for intra-spike crosslinking by M8a-31 IgG.

(F) M8a-31 Fab-Omicron BA.1: open trimer conformation with potential for intra-spike crosslinking by M8a-31 IgG.

(G) M8a-34 Fab-WA1: open trimer conformation with no potential for intra-spike crosslinking by M8a-34 IgG.

(legend continued on next page)

(28–40 Å) (Figure 6A). The other class 4 and 1/4 mAb Fab-bound trimer structures showed larger inter-protomer RBD distances (up to ~70 Å), corresponding to ~11–34 Å more outward displacement of RBDs in comparison with unliganded or class 1- or ACE2-liganded spike trimer structures (Figures 6B–6H).[10] This outward displacement of RBDs could result in spike trimer destabilization, leading to S1 shedding.[11,18,22,26]

Another property of antibodies that could affect their neutralization potencies relates to their ability to utilize bivalency. Since IgG antibodies have two identical Fab arms, they can increase their apparent affinities for binding to tethered antigens through avidity effects, which can occur through either inter-spike cross-linking (simultaneous binding of two neighboring spike trimers) or intra-spike cross-linking (simultaneous binding of two neighboring RBDs within the same spike trimer). To evaluate whether the M8a or HSW mAbs could enhance their binding through intra-spike crosslinking, we measured distances between neighboring Fabs in the Fab-spike structures to predict if simultaneous binding of both IgG Fabs to adjacent RBDs on a trimer would be possible. A distance of ≤65 Å between the C termini of the C_H1 domains of adjacent bound RBD-bound Fabs is required to allow the N-termini of the two chains of an IgG hinge to each of the C-termini of two bound Fabs.[10] Measured distances in spike trimers complexed with the M8a-3 (126, 130, and 159 Å), M8a-34 (107, 110, and 150 Å), or M8a-28 (144 Å) Fabs were too large to permit intra-spike cross-linking (Figures 6C, 6G, and 6I). Although we could not measure analogous distances in the M8a-6-spike structure because only one Fab was bound (Figure 6D), the similar epitope and pose for M8a-3 and M8a-6 (Figures 3A, 3B, 4B, and 4C) suggest that an IgG version of M8a-6 is unlikely to crosslink adjacent RBDs. Thus, the weak binding of M8a-6 to a spike trimer could not be improved by intra-spike crosslinking avidity effects, again rationalizing its lack of neutralizing activity (Figure 2B). For spike trimers complexed with M8a-31 Fab (Figures 6E and 6F), distances between the C termini of adjacent C_H1 domains were measured as 52 and 49 Å for M8a-31 Fab bound to the WA1 and Omicron BA.1 spikes, respectively, suggestive of potential intra-spike crosslinking. We could not evaluate potentials for intra-spike crosslinking for HSW-1 or HSW-2 because either only one Fab was bound per spike (HSW-1) (Figure 5A) or the reconstructions showed Fab binding to dissociated S1 monomer (HSW-2) (Figure 5C).

We also used modeling to assess how the RBD-nanoparticles used to elicit the mAbs investigated here might engage with bivalent BCRs. To address this issue, we asked whether the geometric arrangement of RBDs on mosaic-8 RBD-mi3 nanoparticles would permit bivalent engagement of neighboring RBDs by IgGs, here representing membrane-bound BCRs hypothesized to engage adjacent RBDs (Figure 1D). We first constructed IgG models of each of the Fabs in the M8a and HSW Fab-spike structures (Figures 3 and 5). Next, we asked if it was sterically possible for both Fabs of an IgG to interact with the epitope identified from its cryo-EM structure on adjacent RBDs on a modeled RBD-mi3 nanoparticle. For each of the seven mAb epitopes, we found that the RBD-mi3 nanoparticle geometry was predicted to allow simultaneous recognition of adjacent RBDs by both Fabs of an IgG (Figure S7), thus confirming that the geometric arrangement of RBD attachment sites on SpyCatcher-mi3 would allow BCR engagement through avidity effects.

mAbs elicited by mosaic-8 RBD-nanoparticles resemble EUA-approved therapeutics or a potent cross-reactive human class 1/4 anti-RBD antibody

Human mAbs that received emergency use authorization (EUA) for COVID-19 treatment include class 1 and class 2 anti-RBD mAbs that are no longer effective against SARS-CoV-2 variants and class 3 anti-RBD mAbs, two of which, Bebtelovimab and Cilgavimab, retain at least partial efficacy against Omicron variants (Figures 7A and 7B). The epitope identified for M8a-28 (Figure 7C) resembles epitopes of the class 3 anti-RBD therapeutic mAbs (Figures 7D–7G), as evaluated by comparisons of common RBD epitope buried surface areas (BSAs) (Figure 7B). Some of these mAbs, including M8a-28 (Figure 2B), neutralize Omicron VOCs, but their epitope locations within a region that varies among sarbecoviruses suggests that future SARS-CoV-2 variants are likely to include substitutions that reduce or completely abrogate their efficacies (Figures 7C–7G). By contrast, the more occluded class 1/4 RBD epitope (Figure 7A), to which bound mAbs can inhibit ACE2 receptor binding,[11,23,24] exhibits less variability across sarbecoviruses likely because substitutions that affect its contacts as a "down" RBD with other spike trimer regions limit its variability between SARS-CoV-2 VOCs and other sarbecoviruses.[12]

DISCUSSION

Here, we characterized mouse mAbs elicited by mosaic (M8a mAbs) or homotypic (HSW mAbs) RBD-nanoparticles using both structural and functional analyses, showing that mosaic nanoparticles induce potently neutralizing antibodies that cross-react between animal sarbecoviruses and SARS-CoV-2 VOCs. Although we identified only five mAbs that bound to ≥2 RBDs from mosaic-8 immunized mice in these first experiments, one mAb (M8a-3) was both cross-reactive and strongly neutralizing and two others (M8a-31 and M8a-34) were less potently neutralizing but were cross-reactive against SARS-CoV-2 variants and animal sarbecoviruses. Another mAb (M8a-28) potently neutralized SARS-CoV-2 variants and resembled therapeutic antibodies in current use. Encouragingly, M8a-3, M8a-28, and M8a-31 neutralized all Omicron variants against which they were evaluated (BA.1, BA.2, BA.2.12.1, and BA.4/BA.5), although the Omicron lineage of SARS-CoV-2 had not emerged at the time these experiments were initiated. Structural studies showed that all five mAbs target the desired more conserved epitopes (class 3 and class 1/4) rather than the class 1 and class 2 RBD epitopes more commonly elicited by vaccination or infection.[26,31,32] By contrast, the only two mAbs isolated from

(H) HSW-1 Fab-WA1: open trimer conformation. Black dotted lines between the Cα atoms of C-terminal C_H1 residues are not shown because the reconstruction included only one Fab.
(I) M8a-28 Fab-WA1: closed trimer conformation with no potential for intra-spike crosslinking.
See also Figure S7.

CellPress
OPEN ACCESS

A

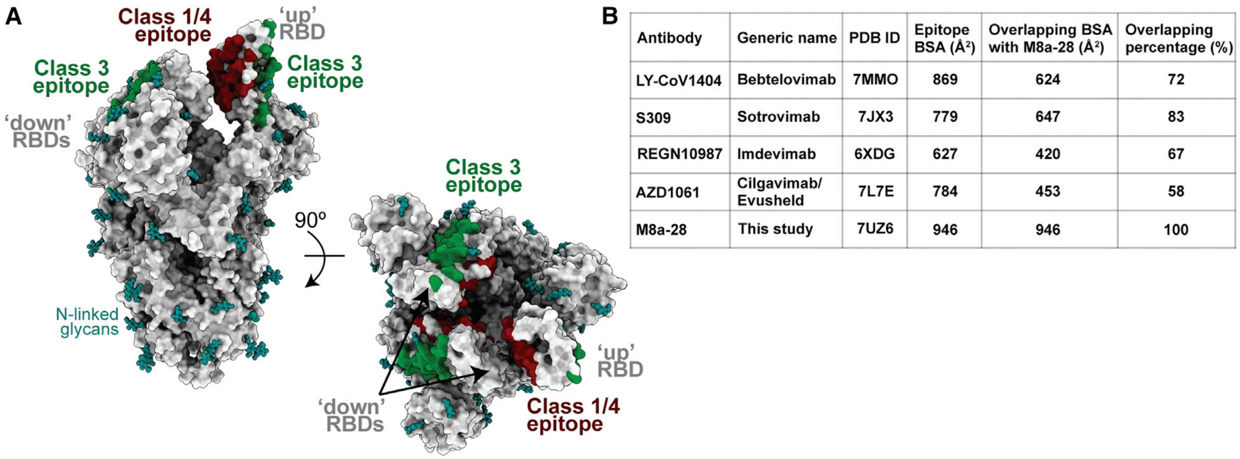

B

Antibody	Generic name	PDB ID	Epitope BSA (Å²)	Overlapping BSA with M8a-28 (Å²)	Overlapping percentage (%)
LY-CoV1404	Bebtelovimab	7MMO	869	624	72
S309	Sotrovimab	7JX3	779	647	83
REGN10987	Imdevimab	6XDG	627	420	67
AZD1061	Cilgavimab/ Evusheld	7L7E	784	453	58
M8a-28	This study	7UZ6	946	946	100

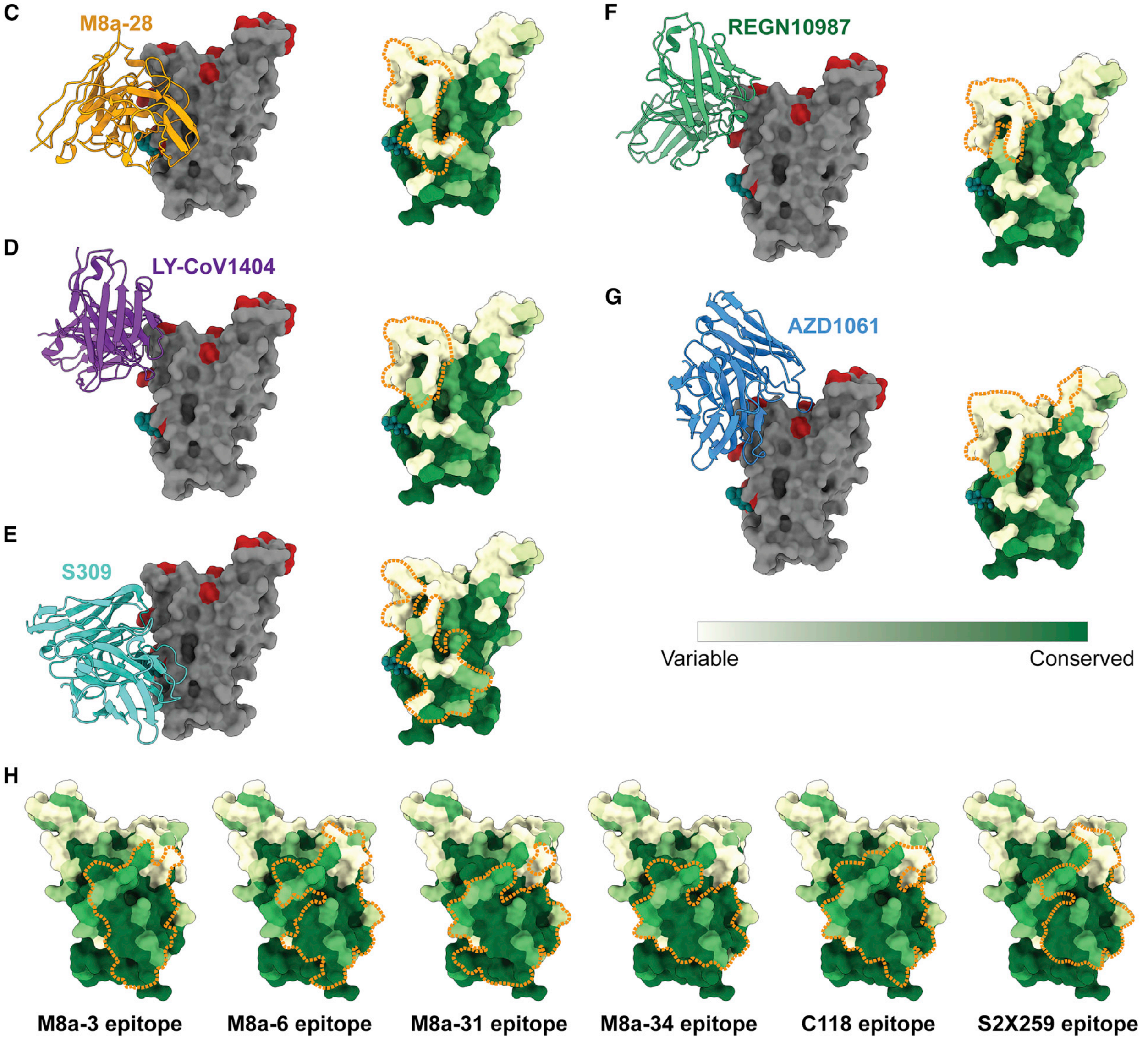

(legend on next page)

homotypic SARS-CoV-2 nanoparticle-immunized mice that were identified as binding ≥2 RBDs during screening targeted different epitopes and were only weakly- or non-neutralizing.

Structural studies of Fab complexes with SARS-CoV-2 spike trimers, including one with Omicron BA.1, demonstrated that four of the five mAbs isolated from mosaic-8 immunized mice recognized conserved epitopes, as designed in the immunization approach and shown for polyclonal antisera raised in mice by mosaic-8 RBD-nanoparticle immunization.[12] By contrast, antibodies raised in homotypic RBD-nanoparticle immunized mice more commonly recognize variable class 1 and class 2 RBD epitopes,[12] likely explaining why it was more difficult in the current study to isolate single B cells from homotypic RBD-nanoparticle immunized mice secreting IgGs that bound ≥2 labeled RBDs. The two cross-RBD binding mAbs we were able to isolate from homotypic RBD-nanoparticle immunized mice showed binding to multiple sarbecovirus RBDs but were only weakly- or non-neutralizing. Corroborating this, the HSW-1-spike structure showed only one bound Fab per trimer compared with three bound Fabs per trimer in the structures of more potently neutralizing mAbs, and the HSW-2 Fab epitope was incompatible with binding to its RBD epitope on intact spike trimer, resulting in a trimer dissociation.

The fact that four of five mouse mAbs identified as binding to ≥2 different RBDs during B cell screening after mosaic-8 immunization target the class 1/4 epitope, in common with the potent, cross-reactive, and protective S2X259 human mAb,[24] supports the potential for using mosaic RBD-nanoparticles as immunogens to efficiently elicit cross-reactive and potent neutralizing mAbs against SARS-CoV-2 variants and animal sarbecoviruses that could spill over to infect humans. In addition, our finding that potent cross-reactive mAbs were identified from relatively few B cells suggest that high-throughput screening of larger samples from animals immunized with mosaic-8 RBD-mi3 could be used to identify many new therapeutic mAbs, which could then be used to prevent or treat infections of Omicron and future SARS-CoV-2 variants. Finally, together with previous challenge and serum epitope mapping studies,[12] these results further validate mosaic-8 RBD-nanoparticles as a broadly protective vaccine candidate.

Limitations of the study

The new mAbs characterized here were derived from immunizations of mice, raising concerns that they could differ from human antibodies elicited by the same immunogens. For example, mouse antibodies generally have shorter CDRH3s than human antibodies.[33] The CDRH3 lengths of the 7 mouse mAbs we characterized structurally ranged from 9 to 16 amino acids (IMGT definition);[34] hence, these mAbs included CDRH3s equivalent to the average length of their human counterparts (15.5 ± 3.2 amino acids).[33] In addition, the class 1/4 and class 4 antibodies primarily elicited by mosaic-8 RBD-nanoparticle immunization[12] tend to rely less on long CDRH3s than, e.g., class 2 anti-RBD antibodies[10] that are less commonly elicited by these immunogens. Another concern is that the murine repertoire might lack V_H and V_L gene segments that provide humans with public responses against SARS-CoV-2 RBDs,[35] of which V_H3-53/V_H3-63,[36,37] V_H3-30,[17] and V_H1-2[38] antibodies have been described. However, epitope mapping of the anti-RBD antibodies including V_H domains encoded by these V gene segments shows that they mainly target more variable RBD epitopes.[35,38] Thus, our working model is that the mouse humoral response to our immunogens is likely to be qualitatively similar to human responses, although particular V gene segments may differ. Future analyses are necessary to

Figure 7. Comparison of M8a epitopes with human mAbs targeting class 3 or class 1/4 RBD epitopes

(A) Locations of class 3 and class 1/4 RBD epitopes mapped on an unliganded spike structure with two "down" and one "up" RBDs (PDB: 6VYB) showing that the class 3 epitope is exposed, whereas the class 1/4 epitope is partially occluded in the context of the spike trimer. The binding epitopes of representative class 3 (S309/Sotrovimab, PDB: 7JX3) and class 1/4 (C118, PDB: 7RKV) anti-RBD antibodies were identified by PDBePISA.[28]

(B) Class 3 anti-RBD mAbs that currently or previously received emergency use authorization (EUA) approval for human administration by the US Food and Drug Administration (modified from Zhou et al.[29]) compared with M8a-28 (this study). Of the human mAbs, only LY-CoV1404/Bebtelovimab retains full neutralization potency against Omicron BA.1,[29] and the NIH COVID-19 treatment guidelines recommend against use of Bamlanivimab plus Etesevimab, Casirivimab plus Imdevimab, or Sotrovimab for the treatment of COVID-19 (US Food and Drug Administration fact sheets listed below). Buried surface areas (BSAs) on the RBD for each mAb are listed.

(C–G) Left: V_H-V_L domains of M8a-28 and currently or previously EUA-approved class 3 anti-RBD mAbs (cartoon representations) shown interacting with an RBD (gray surface representation with Omicron BA.1, BA.2, BA.2.12.1, and BA.4/BA.5 substitutions in red and the RBD Asn343 N-linked glycan shown as teal spheres). Right: mAb epitopes outlined with orange dotted lines on a sequence conservation surface plot (calculated using the 16 sarbecovirus RBD sequences shown in Figure S1).

(C) M8a-28.

(D) LY-CoV1404/Bebtelovimab (PDB: 7MMO).

(E) S309/Sotrovimab (PDB: 7JX3).

(F) REGN10987/Imdevimab (PDB: 6XDG).

(G) AZD1061/Cilgavimab (PDB: 7L7E).

(H) Comparison of the class 1/4 epitopes of M8a mouse mAbs isolated in these studies with the epitopes of human class 1/4 mAbs: C118 (PDB: 7RKV)[11,17] and S2X259 (PDB: 7RAL).[24]

See also Figures S5 and S6.

Food and Drug Administration (2022). Fact sheet for healthcare providers: emergency use authorization (EUA) of Sotrovimab. Available at: https://www.fda.gov/media/149534/download.

Food and Drug Administration (2022). Fact sheet for healthcare providers: emergency use authorization for Evusheld (tixagevimab co-packaged with cilgavimab). Available at: https://www.fda.gov/media/154701/download.

Food and Drug Administration (2022). Fact sheet for health care providers: emergency use authorization (EUA) of bamlanivimab and etesevimab. Available at: https://www.fda.gov/media/145802/download.

Food and Drug Administration (2021). Fact sheet for health care providers: emergency use authorization (EUA) of REGEN-COV (casirivimab and imdevimab). Available at: https://www.fda.gov/media/145611/download.

directly compare antibodies raised in mice versus humans against mosaic-8 RBD-nanoparticle immunogens.

STAR★METHODS

Detailed methods are provided in the online version of this paper and include the following:

- KEY RESOURCES TABLE
- RESOURCE AVAILABILITY
  - Lead contact
  - Materials availability
  - Data and code availability
- EXPERIMENTAL MODEL AND SUBJECT DETAILS
  - Cell lines
  - Bacteria
  - Viruses
- METHOD DETAILS
  - Preparation of homotypic and mosaic-8 RBD-mi3 nanoparticles
  - Immunizations
  - Beacon
  - Cloning
  - IgG and spike trimer production and purification
  - ELISAs
  - Pseudovirus neutralization assays
  - X-ray crystallography
  - Cryo-EM sample preparation
  - Cryo-EM data collection and processing
  - Cryo-EM structure modeling and refinement
  - Structure analyses
  - SPR assays
- QUANTIFICATION AND STATISTICAL ANALYSIS

SUPPLEMENTAL INFORMATION

Supplemental information can be found online at https://doi.org/10.1016/j.immuni.2022.10.019.

ACKNOWLEDGMENTS

We thank J. Vielmetter, P. Hoffman, A. Rorick, K. Storm, and the Caltech Beckman Institute Protein Expression Center for protein production; D. Veesler for BtKY72 neutralization advice; A. Gonzales for isolation and sequencing of positive B cells; the Antibody Discovery Engine and the Drug Discovery and Structural Biology Shared Facility at City of Hope, Songye Chen, and the Caltech Cryo-EM facility for cryo-EM data collection; Jens Kaiser, staff at Stanford Synchrotron Radiation Lightsource, and the Caltech Molecular Observatory for X-ray data collection support; and the Bjorkman lab members for helpful discussions. Cryo-EM was performed in the Beckman Institute Resource Center for Transmission Electron Microscopy at Caltech. Use of the Stanford Synchrotron Radiation Lightsource, SLAC National Accelerator Laboratory, is supported by the U.S. Department of Energy, Office of Science, Office of Basic Energy Sciences under contract no. DE-AC02-76SF00515. The SSRL Structural Molecular Biology Program is supported by the DOE Office of Biological and Environmental Research and by the National Institutes of Health, National Institute of General Medical Sciences (P30GM133894). The contents of this publication are solely the responsibility of the authors and do not necessarily represent the official views of NIGMS or NIH. These studies were funded by the National Institutes of Health (NIH) P01-AI138938-S1 (P.J.B.) and City of Hope's Integrated Drug Development Venture supported by the National Cancer Institute of the National Institutes of Health P30 CA033572 (J.C.W.), Bill and Melinda Gates Foundation INV-034638, INV-004949 (P.J.B.), the Caltech Merkin Institute (P.J.B.), and a George Mason University Fast Grant (P.J.B.).

AUTHOR CONTRIBUTIONS

C.F., A.A.C., J.R.K., H.K., J.C.W., and P.J.B. conceived the study and analyzed the data. C.F. performed single-particle cryo-EM, X-ray crystallography, interpreted structures, and analyzed antibody sequences. A.A.C. prepared nanoparticles and performed negative stain EM. M.P. and A.F.-H.H. isolated B cells and generated mAb sequences. C.F., Y.E.L., and H.G. prepared and purified proteins. A.A.C., J.R.K., and Z.W. performed ELISAs. P.N.P.G. and L.M.K. performed neutralization assays. J.R.K. and C.F. performed SPR assays. C.F., J.R.K., K.E.M., and P.J.B. wrote the paper with contributions from other authors.

DECLARATION OF INTERESTS

P.J.B. serves on the scientific advisory boards for the Vir Biotechnology and Vaccine Company. P.J.B. and A.A.C. are inventors on a US patent application filed by the California Institute of Technology that covers the mosaic nanoparticles described in this work. P.J.B. and A.A.C. are inventors on a US patent application filed by the California Institute of Technology that covers the methodology to generate cross-reactive antibodies using mosaic nanoparticles. P.J.B., A.A.C., C.F., and J.C.W. are inventors on a US patent application filed by the California Institute of Technology that covers the mAbs elicited by vaccination with mosaic-8 RBD-mi3 nanoparticles described in this work. P.J.B., A.A.C., and J.R.K. are inventors on a US patent application filed by the California Institute of Technology that covers the methods of isolating cross-reactive antibodies by vaccination with mosaic nanoparticles.

INCLUSION AND DIVERSITY

We support inclusive, diverse, and equitable conduct of research.

Received: July 4, 2022
Revised: September 7, 2022
Accepted: October 24, 2022
Published: October 26, 2022

REFERENCES

1. Menachery, V.D., Yount, B.L., Debbink, K., Agnihothram, S., Gralinski, L.E., Plante, J.A., Graham, R.L., Scobey, T., Ge, X.Y., Donaldson, E.F., et al. (2015). A SARS-like cluster of circulating bat coronaviruses shows potential for human emergence. Nat. Med. *21*, 1508–1513.
2. Menachery, V.D., Yount, B.L., Sims, A.C., Debbink, K., Agnihothram, S.S., Gralinski, L.E., Graham, R.L., Scobey, T., Plante, J.A., Royal, S.R., et al. (2016). SARS-like WIV1-CoV poised for human emergence. Proc. Natl. Acad. Sci. USA *113*, 3048–3053.
3. Zhou, H., Ji, J., Chen, X., Bi, Y., Li, J., Wang, Q., Hu, T., Song, H., Zhao, R., Chen, Y., et al. (2021). Identification of novel bat coronaviruses sheds light on the evolutionary origins of SARS-CoV-2 and related viruses. Cell *184*, 4380–4391.e14.
4. Burki, T.K. (2022). Omicron variant and booster COVID-19 vaccines. Lancet Respir. Med. *10*, e17. https://doi.org/10.1016/S2213-2600(21)00559-2.
5. Liu, L., Iketani, S., Guo, Y., Chan, J.F., Wang, M., Liu, L., Luo, Y., Chu, H., Huang, Y., Nair, M.S., et al. (2022). Striking antibody evasion manifested by the omicron variant of SARS-CoV-2. Nature *602*, 676–681. https://doi.org/10.1038/s41586-021-04388-0.
6. Planas, D., Bruel, T., Grzelak, L., Guivel-Benhassine, F., Staropoli, I., Porrot, F., Planchais, C., Buchrieser, J., Rajah, M.M., Bishop, E., et al. (2021). Sensitivity of infectious SARS-CoV-2 B.1.1.7 and B.1.351 variants to neutralizing antibodies. Nat. Med. *27*, 917–924.
7. Washington, N.L., Gangavarapu, K., Zeller, M., Bolze, A., Cirulli, E.T., Schiabor Barrett, K.M., Larsen, B.B., Anderson, C., White, S., Cassens, T., et al. (2021). Emergence and rapid transmission of SARS-CoV-2 B.1.1.7 in the United States. Cell *184*, 2587–2594.e7.

8. Bowen, J.E., Addetia, A., Dang, H.V., Stewart, C., Brown, J.T., Sharkey, W.K., Sprouse, K.R., Walls, A.C., Mazzitelli, I.G., Logue, J.K., et al. (2022). Omicron spike function and neutralizing activity elicited by a comprehensive panel of vaccines. Science *377*, 890–894.

9. Starr, T.N., Czudnochowski, N., Liu, Z., Zatta, F., Park, Y.J., Addetia, A., Pinto, D., Beltramello, M., Hernandez, P., Greaney, A.J., et al. (2021). SARS-CoV-2 RBD antibodies that maximize breadth and resistance to escape. Nature *597*, 97–102.

10. Barnes, C.O., Jette, C.A., Abernathy, M.E., Dam, K.-M.A., Esswein, S.R., Gristick, H.B., Malyutin, A.G., Sharaf, N.G., Huey-Tubman, K.E., Lee, Y.E., et al. (2020a). SARS-CoV-2 neutralizing antibody structures inform therapeutic strategies. Nature *588*, 682–687.

11. Jette, C.A., Cohen, A.A., Gnanapragasam, P.N.P., Muecksch, F., Lee, Y.E., Huey-Tubman, K.E., Schmidt, F., Hatziioannou, T., Bieniasz, P.D., Nussenzweig, M.C., et al. (2021). Broad cross-reactivity across sarbecoviruses exhibited by a subset of COVID-19 donor-derived neutralizing antibodies. Cell Rep. *36*, 109760.

12. Cohen, A.A., van Doremalen, N., Greaney, A.J., Andersen, H., Sharma, A., Starr, T.N., Keeffe, J.R., Fan, C., Schulz, J.E., Gnanapragasam, P.N.P., et al. (2022). Mosaic RBD nanoparticles protect against challenge by diverse sarbecoviruses in animal models. Science *377*, eabq0839.

13. Kanekiyo, M., Joyce, M.G., Gillespie, R.A., Gallagher, J.R., Andrews, S.F., Yassine, H.M., Wheatley, A.K., Fisher, B.E., Ambrozak, D.R., Creanga, A., et al. (2019). Mosaic nanoparticle display of diverse influenza virus hemagglutinins elicits broad B cell responses. Nat. Immunol. *20*, 362–372.

14. Landau, M., Mayrose, I., Rosenberg, Y., Glaser, F., Martz, E., Pupko, T., and Ben-Tal, N. (2005). ConSurf 2005: the projection of evolutionary conservation scores of residues on protein structures. Nucleic Acids Res. *33*, W299–W302.

15. Cohen, A.A., Gnanapragasam, P.N.P., Lee, Y.E., Hoffman, P.R., Ou, S., Kakutani, L.M., Keeffe, J.R., Wu, H.J., Howarth, M., West, A.P., et al. (2021). Mosaic nanoparticles elicit cross-reactive immune responses to zoonotic coronaviruses in mice. Science *371*, 735–741.

16. Starr, T.N., Zepeda, S.K., Walls, A.C., Greaney, A.J., Alkhovsky, S., Veesler, D., and Bloom, J.D. (2022). ACE2 binding is an ancestral and evolvable trait of sarbecoviruses. Nature *603*, 913–918.

17. Robbiani, D.F., Gaebler, C., Muecksch, F., Lorenzi, J.C.C., Wang, Z., Cho, A., Agudelo, M., Barnes, C.O., Gazumyan, A., Finkin, S., et al. (2020). Convergent antibody responses to SARS-CoV-2 in convalescent individuals. Nature *584*, 437–442.

18. Pinto, D., Park, Y.-J., Beltramello, M., Walls, A.C., Tortorici, M.A., Bianchi, S., Jaconi, S., Culap, K., Zatta, F., De Marco, A., et al. (2020). Cross-neutralization of SARS-CoV-2 by a human monoclonal SARS-CoV antibody. Nature *583*, 290–295.

19. Davies, D.R., and Metzger, H. (1983). Structural basis of antibody function. Annu. Rev. Immunol. *1*, 87–117.

20. Rettig, T.A., Ward, C., Bye, B.A., Pecaut, M.J., and Chapes, S.K. (2018). Characterization of the naive murine antibody repertoire using unamplified high-throughput sequencing. PLoS One *13*, e0190982.

21. Crawford, K.H.D., Eguia, R., Dingens, A.S., Loes, A.N., Malone, K.D., Wolf, C.R., Chu, H.Y., Tortorici, M.A., Veesler, D., Murphy, M., et al. (2020). Protocol and reagents for pseudotyping lentiviral particles with SARS-CoV-2 spike protein for neutralization assays. Viruses *12*, 513.

22. Huo, J., Zhao, Y., Ren, J., Zhou, D., Duyvesteyn, H.M.E., Ginn, H.M., Carrique, L., Malinauskas, T., Ruza, R.R., Shah, P.N.M., et al. (2020). Neutralization of SARS-CoV-2 by destruction of the prefusion spike. Cell Host Microbe *28*, 445–454.e446.

23. Liu, H., Wu, N.C., Yuan, M., Bangaru, S., Torres, J.L., Caniels, T.G., van Schooten, J., Zhu, X., Lee, C.-C.D., Brouwer, P.J.M., et al. (2020). Cross-neutralization of a SARS-CoV-2 antibody to a functionally conserved site is mediated by avidity. Immunity *53*, 1272–1280.e5.

24. Tortorici, M.A., Czudnochowski, N., Starr, T.N., Marzi, R., Walls, A.C., Zatta, F., Bowen, J.E., Jaconi, S., Di Iulio, J., Wang, Z., et al. (2021). Broad sarbecovirus neutralization by a human monoclonal antibody. Nature *597*, 103–108.

25. Hsieh, C.L., Goldsmith, J.A., Schaub, J.M., DiVenere, A.M., Kuo, H.C., Javanmardi, K., Le, K.C., Wrapp, D., Lee, A.G., Liu, Y., et al. (2020). Structure-based design of prefusion-stabilized SARS-CoV-2 spikes. Science *369*, 1501–1505.

26. Piccoli, L., Park, Y.-J., Tortorici, M.A., Czudnochowski, N., Walls, A.C., Beltramello, M., Silacci-Fregni, C., Pinto, D., Rosen, L.E., Bowen, J.E., et al. (2020). Mapping neutralizing and immunodominant sites on the SARS-CoV-2 spike receptor-binding domain by structure-guided high-resolution serology. Cell *183*, 1024–1042.e21. https://doi.org/10.1016/j.cell.2020.09.037.

27. Wec, A.Z., Wrapp, D., Herbert, A.S., Maurer, D.P., Haslwanter, D., Sakharkar, M., Jangra, R.K., Dieterle, M.E., Lilov, A., Huang, D., et al. (2020). Broad neutralization of SARS-related viruses by human monoclonal antibodies. Science *369*, 731–736.

28. Krissinel, E., and Henrick, K. (2007). Inference of macromolecular assemblies from crystalline state. J. Mol. Biol. *372*, 774–797.

29. Zhou, T., Wang, L., Misasi, J., Pegu, A., Zhang, Y., Harris, D.R., Olia, A.S., Talana, C.A., Yang, E.S., Chen, M., et al. (2022). Structural basis for potent antibody neutralization of SARS-CoV-2 variants including B.1.1.529. Science *376*, eabn8897.

30. Niu, L., Wittrock, K.N., Clabaugh, G.C., Srivastava, V., and Cho, M.W. (2021). A structural landscape of neutralizing antibodies against SARS-CoV-2 receptor binding domain. Front. Immunol. *12*, 647934.

31. Greaney, A.J., Loes, A.N., Crawford, K.H.D., Starr, T.N., Malone, K.D., Chu, H.Y., and Bloom, J.D. (2021a). Comprehensive mapping of mutations in the SARS-CoV-2 receptor-binding domain that affect recognition by polyclonal human plasma antibodies. Cell Host Microbe *29*, 463–476.e6.

32. Greaney, A.J., Starr, T.N., Barnes, C.O., Weisblum, Y., Schmidt, F., Caskey, M., Gaebler, C., Cho, A., Agudelo, M., Finkin, S., et al. (2021b). Mapping mutations to the SARS-CoV-2 RBD that escape binding by different classes of antibodies. Nat. Commun. *12*, 4196.

33. Shi, B., Ma, L., He, X., Wang, X., Wang, P., Zhou, L., and Yao, X. (2014). Comparative analysis of human and mouse immunoglobulin variable heavy regions from IMGT/LIGM-DB with IMGT/HighV-QUEST. Theor. Biol. Med. Model. *11*, 30.

34. Lefranc, M.P., Giudicelli, V., Duroux, P., Jabado-Michaloud, J., Folch, G., Aouinti, S., Carillon, E., Duvergey, H., Houles, A., Paysan-Lafosse, T., et al. (2015). IMGT(R), the international ImMunoGeneTics information system(R) 25 years on. Nucleic Acids Res. *43*, D413–D422.

35. Chen, E.C., Gilchuk, P., Zost, S.J., Suryadevara, N., Winkler, E.S., Cabel, C.R., Binshtein, E., Chen, R.E., Sutton, R.E., Rodriguez, J., et al. (2021). Convergent antibody responses to the SARS-CoV-2 spike protein in convalescent and vaccinated individuals. Cell Rep. *36*, 109604. https://doi.org/10.1016/j.celrep.2021.109604.

36. Barnes, C.O., West, A.P., Jr., Huey-Tubman, K.E., Hoffmann, M.A.G., Sharaf, N.G., Hoffman, P.R., Koranda, N., Gristick, H.B., Gaebler, C., Muecksch, F., et al. (2020b). Structures of human antibodies bound to SARS-CoV-2 spike reveal common epitopes and recurrent features of antibodies. Cell *182*, 828–842.e16.

37. Yuan, M., Liu, H., Wu, N.C., Lee, C.-C.D., Zhu, X., Zhao, F., Huang, D., Yu, W., Hua, Y., Tien, H., et al. (2020). Structural basis of a shared antibody response to SARS-CoV-2. Science *369*, 1119–1123. https://doi.org/10.1126/science.abd2321.

38. Rapp, M., Guo, Y., Reddem, E.R., Yu, J., Liu, L., Wang, P., Cerutti, G., Katsamba, P., Bimela, J.S., Bahna, F.A., et al. (2021). Modular basis for potent SARS-CoV-2 neutralization by a prevalent VH1-2-derived antibody class. Cell Rep. *35*, 108950.

39. Scheid, J.F., Barnes, C.O., Eraslan, B., Hudak, A., Keeffe, J.R., Cosimi, L.A., Brown, E.M., Muecksch, F., Weisblum, Y., Zhang, S., et al. (2021). B cell genomics behind cross-neutralization of SARS-CoV-2 variants and SARS-CoV. Cell *184*, 3205–3221.e24.

CellPress
OPEN ACCESS

40. Pear, W.S., Nolan, G.P., Scott, M.L., and Baltimore, D. (1993). Production of high-titer helper-free retroviruses by transient transfection. Proc. Natl. Acad. Sci. USA *90*, 8392–8396.

41. Elbe, S., and Buckland-Merrett, G. (2017). Data, disease and diplomacy: GISAID's innovative contribution to global health. Glob. Chall. *1*, 33–46.

42. Shu, Y., and McCauley, J. (2017). GISAID: Global initiative on sharing all influenza data - from vision to reality. Euro Surveill. *22*, 30494.

43. Mastronarde, D.N. (2005). Automated electron microscope tomography using robust prediction of specimen movements. J. Struct. Biol. *152*, 36–51.

44. Punjani, A., Rubinstein, J.L., Fleet, D.J., and Brubaker, M.A. (2017). cryoSPARC: algorithms for rapid unsupervised cryo-EM structure determination. Nat. Methods *14*, 290–296.

45. Pettersen, E.F., Goddard, T.D., Huang, C.C., Couch, G.S., Greenblatt, D.M., Meng, E.C., and Ferrin, T.E. (2004). UCSF Chimera–a visualization system for exploratory research and analysis. J. Comput. Chem. *25*, 1605–1612.

46. Goddard, T.D., Huang, C.C., and Ferrin, T.E. (2007). Visualizing density maps with UCSF Chimera. J. Struct. Biol. *157*, 281–287.

47. Goddard, T.D., Huang, C.C., Meng, E.C., Pettersen, E.F., Couch, G.S., Morris, J.H., and Ferrin, T.E. (2018). UCSF ChimeraX: meeting modern challenges in visualization and analysis. Protein Sci. *27*, 14–25.

48. Pettersen, E.F., Goddard, T.D., Huang, C.C., Meng, E.C., Couch, G.S., Croll, T.I., Morris, J.H., and Ferrin, T.E. (2021). UCSF ChimeraX: structure visualization for researchers, educators, and developers. Protein Sci. *30*, 70–82.

49. Kabsch, W. (2010). Xds. Acta Crystallogr. D Biol. Crystallogr. *66*, 125–132.

50. McCoy, A.J. (2007). Solving structures of protein complexes by molecular replacement with Phaser. Acta Crystallogr. D Biol. Crystallogr. *63*, 32–41.

51. Liebschner, D., Afonine, P.V., Baker, M.L., Bunkóczi, G., Chen, V.B., Croll, T.I., Hintze, B., Hung, L.W., Jain, S., McCoy, A.J., et al. (2019). Macromolecular structure determination using X-rays, neutrons and electrons: recent developments in Phenix. Acta Crystallogr. D Struct. Biol. *75*, 861–877.

52. Emsley, P., Lohkamp, B., Scott, W.G., and Cowtan, K. (2010). Features and development of coot. Acta Crystallogr. D Biol. Crystallogr. *66*, 486–501.

53. Winn, M.D., Ballard, C.C., Cowtan, K.D., Dodson, E.J., Emsley, P., Evans, P.R., Keegan, R.M., Krissinel, E.B., Leslie, A.G., McCoy, A., et al. (2011). Overview of the CCP4 suite and current developments. Acta Crystallogr. D Biol. Crystallogr. *67*, 235–242.

54. Chen, V.B., Arendall, W.B., 3rd, Headd, J.J., Keedy, D.A., Immormino, R.M., Kapral, G.J., Murray, L.W., Richardson, J.S., and Richardson, D.C. (2010). MolProbity: all-atom structure validation for macromolecular crystallography. Acta Crystallogr. D Biol. Crystallogr. *66*, 12–21.

55. Lilkova, E., Petkov, P., Ilieva, N., and Litov, L. (2015). The PyMOL Molecular Graphics System, Version 1.8 (Schrodinger, LLC).

56. Dunbar, J., Krawczyk, K., Leem, J., Baker, T., Fuchs, A., Georges, G., Shi, J., and Deane, C.M. (2014). SAbDab: the structural antibody database. Nucleic Acids Res. *42*, D1140–D1146.

57. Keeble, A.H., Turkki, P., Stokes, S., Khairil Anuar, I.N.A., Rahikainen, R., Hytönen, V.P., and Howarth, M. (2019). Approaching infinite affinity through engineering of peptide–protein interaction. Proc. Natl. Acad. Sci. USA *116*, 26523–26533.

58. Gibson, D.G., Glass, J.I., Lartigue, C., Noskov, V.N., Chuang, R.Y., Algire, M.A., Benders, G.A., Montague, M.G., Ma, L., Moodie, M.M., et al. (2010). Creation of a bacterial cell controlled by a chemically synthesized genome. Science *329*, 52–56.

59. Gibson, D.G., Young, L., Chuang, R.Y., Venter, J.C., Hutchison, C.A., 3rd, and Smith, H.O. (2009). Enzymatic assembly of DNA molecules up to several hundred kilobases. Nat. Methods *6*, 343–345.

60. Dunbar, J., Krawczyk, K., Leem, J., Marks, C., Nowak, J., Regep, C., Georges, G., Kelm, S., Popovic, B., and Deane, C.M. (2016). SAbPred: a structure-based antibody prediction server. Nucleic Acids Res. *44*, W474–W478.

61. Escolano, A., Gristick, H.B., Gautam, R., DeLaitsch, A.T., Abernathy, M.E., Yang, Z., Wang, H., Hoffmann, M.A.G., Nishimura, Y., Wang, Z., et al. (2021). Sequential immunization of macaques elicits heterologous neutralizing antibodies targeting the V3-glycan patch of HIV-1 Env. Sci. Transl. Med. *13*, eabk1533.

62. West, A.P., Jr., Scharf, L., Horwitz, J., Klein, F., Nussenzweig, M.C., and Bjorkman, P.J. (2013). Computational analysis of anti-HIV-1 antibody neutralization panel data to identify potential functional epitope residues. Proc. Natl. Acad. Sci. USA *110*, 10598–10603.

63. McPhillips, T.M., McPhillips, S.E., Chiu, H.J., Cohen, A.E., Deacon, A.M., Ellis, P.J., Garman, E., Gonzalez, A., Sauter, N.K., Phizackerley, R.P., et al. (2002). Blu-Ice and the Distributed Control System: software for data acquisition and instrument control at macromolecular crystallography beamlines. J. Synchrotron Radiat. *9*, 401–406.

64. Rich, R.L., and Myszka, D.G. (2010). Grading the commercial optical biosensor literature-Class of 2008: 'The Mighty Binders'. J. Mol. Recognit. *23*, 1–64.

65. Rich, R.L., and Myszka, D.G. (2011). Survey of the 2009 commercial optical biosensor literature. J. Mol. Recognit. *24*, 892–914.

66. Wlodawer, A., Minor, W., Dauter, Z., and Jaskolski, M. (2013). Protein crystallography for aspiring crystallographers or how to avoid pitfalls and traps in macromolecular structure determination. FEBS J. *280*, 5705–5736.

67. Scheres, S.H., and Chen, S. (2012). Prevention of overfitting in cryo-EM structure determination. Nat. Methods *9*, 853–854.

68. Henderson, R. (2013). Avoiding the pitfalls of single particle cryo-electron microscopy: einstein from noise. Proc. Natl. Acad. Sci. USA *110*, 18037–18041.

STAR★METHODS

KEY RESOURCES TABLE

REAGENT or RESOURCE	SOURCE	IDENTIFIER
**Antibodies**		
Goat Anti-Human IgG(H+L)-HRP	SouthernBiotech	Cat# 2015-05; RRID:AB_2795588
Goat Anti-Human IgG Fc-HRP	SouthernBiotech	Cat# 2014-05; RRID:AB_2795580
**Bacterial and virus strains**		
E. coli DH5 Alpha	Zymo Research	Cat# T3009
E. coli BL21-CodonPlus (DE3)-RIPL	Agilent Technology	Cat# 230280
SARS-CoV-2 pseudotyped reporter virus	BEI	Cat# NR-53817
SARS-CoV-2 Beta pseudotyped reporter virus	Scheid et al.[39]	https://linkinghub.elsevier.com/retrieve/pii/S0092867421005353
SARS-CoV-2 Delta pseudotyped reporter virus	Cohen et al.[12]	https://www.science.org/10.1126/science.abq0839
SARS-CoV-2 Omicron BA.1 pseudotyped reporter virus	Cohen et al.[12]	https://www.science.org/10.1126/science.abq0839
SARS-CoV-2 Omicron BA.2 pseudotyped reporter virus	Cohen et al.[12]	https://www.science.org/10.1126/science.abq0839
SARS-CoV-2 Omicron BA.2.12.1 pseudotyped reporter virus	Cohen et al.[12]	https://www.science.org/10.1126/science.abq0839
SARS-CoV-2 Omicron BA.4/BA.5 pseudotyped reporter virus	Cohen et al.[12]	https://www.science.org/10.1126/science.abq0839
SARS-CoV pseudotyped reporter virus	Robbiani et al.[17]	https://www.nature.com/articles/s41586-020-2456-9
WIV1-CoV pseudotyped reporter virus	Cohen et al.[15]	https://www.science.org/10.1126/science.abf6840
SHC014-CoV pseudotyped reporter virus	Cohen et al.[15]	https://www.science.org/10.1126/science.abf6840
BtKY72-CoV K493Y-T498W pseudotyped reporter virus	Starr et al.[16]	https://www.nature.com/articles/s41586-022-04464-z
Khosta2/SARS-CoV chimera	Cohen et al.[12]	https://www.science.org/10.1126/science.abq0839
LYRa3/SARS-CoV chimera	Cohen et al.[12]	https://www.science.org/10.1126/science.abq0839
**Chemicals, peptides, and recombinant proteins**		
Dulbecco's Modified Eagle Medium (DMEM)	Gibco	Cat# 11960-044
Fetal bovine serum (FBS)	Sigma-Aldrich	Cat# F4135
Gentamicin solution	Sigma-Aldrich	Cat# G1397 CAS:1405-41-0
Blasticidin S HCl	Gibco	Cat# A1113902 CAS:3513-03-9
Expi293™ Expression Medium	Gibco	Cat# A1435102
Expi293 Expression System Kit	Gibco	Cat# A14635
LB Broth (Miller)	Sigma-Aldrich	Cat# L3522
HBS-EP+ Buffer 20x	Teknova	Cat# H8022
BLI Mouse Plasma Cell Media	Berkeley Lights	Cat# 750-70004
BirA biotin-protein ligase standard reaction kit	Avidity	Cat# BirA500
SuperSignal ELISA Femto Maximum Sensitivity Substrate	Thermo Fisher Scientific	Cat# 37074
HRP-conjugated streptavidin	SouthernBiotech	Cat# 7105-05
Fluorinated octylmaltoside	Anatrace	Cat# O310F

(*Continued on next page*)

Continued

REAGENT or RESOURCE	SOURCE	IDENTIFIER
**Critical commercial assays**		
Luciferase Cell Culture Lysis 5X Reagent	Promega	Cat# E1531
Nano-Glo Luciferase Assay System	Promega	Cat# N1110
EZ-link NHS-PEG4 Biotinylation Kit	Thermo Fisher Scientific	Cat# 21455
**Deposited data**		
SARS-CoV-2 WA1 S 6P + M8a-3 Fab complex coordinate	This paper	PDB: 7UZ4
SARS-CoV-2 WA1 S 6P + M8a-3 Fab complex cryo-EM map	This paper	EMDB: EMD-26878
SARS-CoV-2 WA1 S 6P + M8a-6 Fab complex coordinate	This paper	PDB: 7UZ5
SARS-CoV-2 WA1 S 6P + M8a-6 Fab complex cryo-EM map	This paper	EMDB: EMD-26879
SARS-CoV-2 WA1 S 6P + M8a-28 Fab complex coordinate	This paper	PDB: 7UZ6
SARS-CoV-2 WA1 S 6P + M8a-28 Fab complex cryo-EM map	This paper	EMDB: EMD-26880
SARS-CoV-2 WA1 S 6P + M8a-31 Fab complex coordinate	This paper	PDB: 7UZ7
SARS-CoV-2 WA1 S 6P + M8a-31 Fab complex cryo-EM map	This paper	EMDB: EMD-26881
SARS-CoV-2 WA1 S 6P + M8a-34 Fab complex coordinate	This paper	PDB: 7UZ9
SARS-CoV-2 WA1 S 6P + M8a-34 Fab complex cryo-EM map	This paper	EMDB: EMD-26883
SARS-CoV-2 WA1 S 6P + HSW-1 Fab complex coordinate	This paper	PDB: 7UZA
SARS-CoV-2 WA1 S 6P + HSW-1 Fab complex cryo-EM map	This paper	EMDB: EMD-26884
SARS-CoV-2 WA1 S 6P + HSW-2 Fab complex coordinate	This paper	PDB: 7UZB
SARS-CoV-2 WA1 S 6P + HSW-2 Fab complex cryo-EM map	This paper	EMDB: EMD-26885
SARS-CoV-2 Omicron BA.1 S 6P + M8a-31 Fab complex coordinate	This paper	PDB: 7UZ8
SARS-CoV-2 Omicron BA.1 S 6P + M8a-31 Fab complex cryo-EM map	This paper	EMDB: EMD-26882
SARS-CoV-2 RBD + M8a-34 Fab crystal structure	This paper	PDB: 7UZC
SARS-CoV-2 RBD + HSW-2 Fab crystal structure	This paper	PDB: 7UZD
**Experimental models: Cell lines**		
HEK293T cells	Pear et al.[40]	Cat# CCLV-RIE 1018<br>RRID: CVCL_0063
HEK293T$_{Ace2}$ cells	BEI	Cat# NR-52511
Expi293F cells	Gibco	Cat# A14527<br>RRID: CVCL_D615
**Experimental models: Organisms/strains**		
C57BL/6 mice (4–6-week-old female)	Charles River	N/A
**Recombinant DNA**		
pPPI4-SARS-CoV-2 S 6P	Hsieh et al.[25]	N/A
p3BNC-SARS-CoV-2 RBD (residues 323-528)	This paper	N/A

(*Continued on next page*)

Continued

REAGENT or RESOURCE	SOURCE	IDENTIFIER
p3BNC-SARS-CoV-2 Omicron BA.1 S 6P	This paper	N/A
p3BNC-M8a-3 IgG HC<br>p3BNC-M8a-3 Fab HC<br>p3BNC-M8a-3 LC	This paper	N/A
p3BNC-M8a-6 IgG HC<br>p3BNC-M8a-6 Fab HC<br>p3BNC-M8a-6 LC	This paper	N/A
p3BNC-M8a-28 IgG HC<br>p3BNC-M8a-28 Fab HC<br>p3BNC-M8a-28 LC	This paper	N/A
p3BNC-M8a-31 IgG HC<br>p3BNC-M8a-31 Fab HC<br>p3BNC-M8a-31 LC	This paper	N/A
p3BNC-M8a-34 IgG HC<br>p3BNC-M8a-34 Fab HC<br>p3BNC-M8a-34 LC	This paper	N/A
p3BNC-HSW-1 IgG HC<br>p3BNC-HSW-1 Fab HC<br>p3BNC-HSW-1 LC	This paper	N/A
p3BNC-HSW-2 IgG HC<br>p3BNC-HSW-2 Fab HC<br>p3BNC-HSW-2 LC	This paper	N/A
p3BNC-M8a-7 IgG HC<br>p3BNC-M8a-7 LC	This paper	N/A
p3BNC-M8a-11 IgG HC<br>p3BNC-M8a-11 LC	This paper	N/A
p3BNC-M8a-15 IgG HC<br>p3BNC-M8a-15 LC	This paper	N/A
p3BNC-M8a-25 IgG HC<br>p3BNC-M8a-25 LC	This paper	N/A
p3BNC-M8a-29 IgG HC<br>p3BNC-M8a-29 LC	This paper	N/A
p3BNC-M8a-30 IgG HC<br>p3BNC-M8a-30 LC	This paper	N/A
C102 IgG HC<br>C102 LC	Barnes et al.[10]	https://www.nature.com/articles/s41586-020-2852-1
C144 IgG HC<br>C144 LC	Robbiani et al.[17]	https://www.nature.com/articles/s41586-020-2456-9
S309 IgG HC<br>S309 LC	Pinto et al.[18]	https://www.nature.com/articles/s41586-020-2349-y
C118 IgG HC<br>C118 LC	Robbiani et al.[17]	https://www.nature.com/articles/s41586-020-2456-9
p3BNC-SARS-CoV-2 WA1 RBD SpyTag<br>p3BNC-SARS-CoV-2 WA1 RBD HisAvi	Cohen et al.[15]	https://www.science.org/10.1126/science.abf6840
p3BNC-SARS-CoV-2 Beta RBD SpyTag<br>p3BNC-SARS-CoV-2 Beta RBD HisAvi	Cohen et al.[12]	https://www.science.org/10.1126/science.abq0839
p3BNC-SARS-CoV-2 Delta RBD SpyTag<br>p3BNC-SARS-CoV-2 Delta RBD HisAvi	Cohen et al.[12]	https://www.science.org/10.1126/science.abq0839
p3BNC-SARS-CoV-2 Omicron BA.1 RBD SpyTag<br>p3BNC-SARS-CoV-2 Omicron BA.1 RBD HisAvi	Cohen et al.[12]	https://www.science.org/10.1126/science.abq0839
p3BNC-SARS-CoV-2 Omicron BA.2 RBD SpyTag<br>p3BNC-SARS-CoV-2 Omicron BA.2 RBD HisAvi	Cohen et al.[12]	https://www.science.org/10.1126/science.abq0839
p3BNC-SARS-CoV-2 Omicron BA.2.12.1 RBD SpyTag<br>p3BNC-SARS-CoV-2 Omicron BA.2.12.1 RBD HisAvi	Cohen et al.[12]	https://www.science.org/10.1126/science.abq0839

(*Continued on next page*)

Continued

REAGENT or RESOURCE	SOURCE	IDENTIFIER
p3BNC-SARS-CoV-2 Omicron BA.4/BA.5 RBD SpyTag<br>p3BNC-SARS-CoV-2 Omicron BA.4/BA.5 RBD HisAvi	Cohen et al.[12]	https://www.science.org/10.1126/science.abq0839
p3BNC-RsSTT200-CoV RBD SpyTag<br>p3BNC-RsSTT200-CoV RBD HisAvi	Cohen et al.[12]	https://www.science.org/10.1126/science.abq0839
p3BNC-Pang17-CoV RBD HisAvi<br>p3BNC-Pang17-CoV RBD SpyTag	Cohen et al.[15]	https://www.science.org/10.1126/science.abf6840
p3BNC-RaTG13-CoV RBD SpyTag<br>p3BNC-RaTG13-CoV RBD HisAvi	Cohen et al.[15]	https://www.science.org/10.1126/science.abf6840
p3BNC-SARS-CoV RBD SpyTag<br>p3BNC-SARS-CoV RBD HisAvi	Cohen et al.[15]	https://www.science.org/10.1126/science.abf6840
p3BNC-WIV1-CoV RBD SpyTag<br>p3BNC-WIV1-CoV RBD HisAvi	Cohen et al.[15]	https://www.science.org/10.1126/science.abf6840
p3BNC-SHC014-CoV RBD SpyTag<br>p3BNC-SHC014-CoV RBD HisAvi	Cohen et al.[15]	https://www.science.org/10.1126/science.abf6840
p3BNC-LYRa3-CoV RBD SpyTag<br>p3BNC-LYRa3-CoV RBD HisAvi	Cohen et al.[15]	https://www.science.org/10.1126/science.abq0839
p3BNC-C028 RBD SpyTag<br>p3BNC-C028 RBD HisAvi	This paper	N/A
p3BNC-Rs4081-CoV RBD SpyTag<br>p3BNC-Rs4081-CoV RBD HisAvi	Cohen et al.[15]	https://www.science.org/10.1126/science.abf6840
p3BNC-RmYN02-CoV RBD SpyTag<br>p3BNC-RmYN02-CoV RBD HisAvi	Cohen et al.[15]	https://www.science.org/10.1126/science.abf6840
p3BNC-Rf1-CoV RBD SpyTag<br>p3BNC-Rf1-CoV RBD HisAvi	Cohen et al.[15]	https://www.science.org/10.1126/science.abf6840
p3BNC-Yun11-CoV RBD SpyTag<br>p3BNC-Yun11-CoV RBD HisAvi	Cohen et al.[15]	https://www.science.org/10.1126/science.abf6840
p3BNC-BM4831-CoV RBD SpyTag<br>p3BNC-BM4831-CoV RBD HisAvi	Cohen et al.[15]	https://www.science.org/10.1126/science.abf6840
p3BNC-BtKY72-CoV RBD SpyTag<br>p3BNC-BtKY72-CoV RBD HisAvi	Cohen et al.[15]	https://www.science.org/10.1126/science.abf6840
p3BNC-Khosta2-CoV RBD SpyTag<br>p3BNC-Khosta2-CoV RBD HisAvi	Cohen et al.[12]	https://www.science.org/10.1126/science.abq0839
Software and algorithms		
GISAID	Elbe and Buckland-Merrett[41] and Shu and McCauley[42]	https://www.gisaid.org<br>RRID:SCR_018251
Geneious	Geneious	https://www.geneious.com/
Prism v9.3.1	GraphPad	https://www.graphpad.com/scientific-software/prism/<br>RRID:SCR_002798
SerialEM 3.7	Mastronarde[43]	https://bio3d.colorado.edu/SerialEM/<br>RRID:SCR_017293
cryoSPARC 3.2	Punjani et al.[44]	https://www.cryosparc.com<br>RRID:SCR_016501
UCSF Chimera	Goddard et al.[45] and Pettersen et al.[46]	http://plato.cgl.ucsf.edu/chimera/<br>RRID:SCR_004097
UCSF ChimeraX	Goddard et al.[47] and Pettersen et al.[48]	https://www.cgl.ucsf.edu/chimerax/<br>RRID:SCR_015872
XDS	Kabsch[49]	http://xds.mpimf-heidelberg.mpg.de<br>RRID:SCR_015652
PHASER	McCoy[50]	https://www.phenix-online.org/documentation/reference/phaser.html<br>RRID:SCR_014219
Phenix	Liebschner et al.[51]	https://www.phenix-online.org/<br>RRID:SCR_014224

(*Continued on next page*)

Continued		
REAGENT or RESOURCE	SOURCE	IDENTIFIER
Coot	Emsley et al.[52]	https://www2.mrc-lmb.cam.ac.uk/personal/pemsley/coot/ RRID:SCR_014222
AIMLESS	Winn et al.[53]	http://www.ccp4.ac.uk/html/aimless.html RRID:SCR_015747
MolProbity	Chen et al.[54]	http://molprobity.biochem.duke.edu RRID:SCR_014226
PyMOL 2.4.0	Lilkova et al.[55]	https://pymol.org/2/ RRID:SCR_000305
ConSurf Database	Landau et al.[14]	https://consurf.tau.ac.il RRID:SCR_002320
SAbDab	Dunbar et al.[56]	http://opig.stats.ox.ac.uk/webapps/newsabdab/sabdab/
PDBePISA	Krissinel and Henrick[28]	https://www.ebi.ac.uk/pdbe/pisa/
Bruker Sierra SPR-32 Pro analysis software	Bruker	https://www.bruker.com/en/products-and-solutions/surface-plasmon-resonance/sierra-spr-32-pro.html
Illustrator	Adobe	https://www.adobe.com
Other		
384-well Nunc MaxiSorp ELISA plate	Millipore Sigma	Cat# P6491
100kDa cutoff Amicon concentrator	EMD Millipore	Cat# UFC910096
30kDa cutoff Amicon concentrator	EMD Millipore	Cat# UFC903096
10kDa cutoff Amicon concentrator	EMD Millipore	Cat# UFC901096
HisTrap HP	Cytiva	Cat# 17-5248-02
HiLoad 16/600 Superdex 200 pg	Cytiva	Cat# 28-9893-35
Superose 6 Increase 10/300 GL	Cytiva	Cat# 29-0915-96
HiTrap MabSelect SuRe	Cytiva	Cat# 11-0034-95
Superdex 200 Increase 10/300 GL	Cytiva	Cat# 28-9909-44
400 Mesh carbon-coated copper grids	Ted Pella	Cat# 01844-F
300 Mesh Quantifoil holey carbon 1.2/1.3 cooper grids	Electron Microscopy Sciences	Cat# Q350AR13A
High Capacity Amine Sensor chip	Bruker	Cat# 1862614
OptoSelect chip 11k	Berkeley Lights	Cat# 750-08090
BLI assay beads	Berkeley Lights	Cat# 520-00053

RESOURCE AVAILABILITY

Lead contact

Further information and requests for resources and reagents should be directed to and will be by the lead contact, Pamela J. Bjorkman: bjorkman@caltech.edu.

Materials availability

All expression plasmids generated in this study for CoV proteins, CoV pseudoviruses, mouse Fabs and IgGs are available upon request through a Materials Transfer Agreement.

Data and code availability

Atomic models and cryo-EM maps generated from cryo-EM studies of the M8a-3–WA1 spike 6P, M8a-6–WA1 spike 6P, M8a-28–WA1 spike 6P, M8a-31–WA1 spike 6P, M8a-31–Omicron BA.1 spike 6P, M8a-34–WA1 spike 6P, HSW-1–WA1 spike 6P, and HSW-2–WA1 spike S1 domain complexes have been deposited at the Protein Data Bank (PDB) and Electron Microscopy Data Bank (EMDB) under the following accession codes: PDB: 7UZ4, 7UZ5, 7UZ6, 7UZ7, 7UZ8, 7UZ9, 7UZA, and 7UZB; EMDB: EMD-26878, EMD-26879, EMD-26880, EMD-26881, EMD-26882, EMD-26883, EMD-26884, and EMD-26885. Atomic models

CellPress
OPEN ACCESS

generated from crystal structures of M8a-34–RBD and HSW-2–RBD complexes have been deposited at the PDB under accession codes PDB: 7UZC and 7UZD, respectively. Additional information required to analyze the data reported in this paper is available from the lead contact upon request.

This paper does not report original code.

EXPERIMENTAL MODEL AND SUBJECT DETAILS

Cell lines

HEK293T cells were cultured in Dulbecco's modified Eagle's medium (DMEM, Gibco) supplemented with 10% heat-inactivated fetal bovine serum (FBS, Sigma-Aldrich) and 5 mg/ml gentamicin (Sigma-Aldrich) at 37 °C and 5% CO_2 for pseudovirus production.

$HEK293T_{ACE2}$ cells were cultured in DMEM (Gibco) supplemented with 10% heat-inactivated FBS (Sigma-Aldrich), 5 mg/ml gentamicin (Sigma-Aldrich), and 5 mg/mL blasticidin (Gibco) at 37 °C and 5% CO_2 as described previously[17] for pseudovirus neutralization experiments.

Expi293T cells (Gibco) for protein expression were maintained at 37 °C and 8% CO_2 in Expi293 expression medium (Gibco). Transfections were carried out with an Expi293 Expression System Kit (Gibco) and maintained under shaking at 130 rpm. All cell lines were derived from female donors and were not specially authenticated.

Bacteria

E. coli DH5 Alpha cells (Zymo Research) used for expression plasmid productions were cultured in LB broth (Sigma-Aldrich) with shaking at 250 rpm at 37 °C.

E. coli BL21-CodonPlus (DE3)-RIPL (Agilent Technology) used for producing SpyCatcher003-mi3 were cultured in 2xYT media 220 rpm at 37 °C, IPTG was added at OD of 0.5 and induction lasted for 5 hours at 30°C.

Viruses

Pseudovirus stocks were generated by transfecting HEK293T cells with pNL4-3DEnv-nanoluc and coronavirus pseudovirus constructs[17] using polyethyleneimine; co-transfection of pNL4-3DEnv-nanoluc with a coronavirus construct will lead to the production of HIV-1-based pseudovirions carrying the coronavirus spike protein at the surface. Eight hours after the transfection, cells were washed twice with phosphate buffered saline (PBS) and fresh media was added. Pseudoviruses in the supernatants were harvested 48 hours post-transfection, filtered, and stored in -80 °C until use. Infectivity of pseudoviruses was determined by titration on $HEK293T_{ACE2}$ cells.

METHOD DETAILS

Preparation of homotypic and mosaic-8 RBD-mi3 nanoparticles

Mammalian expression vectors encoding RBDs of SARS-CoV-2 and other sarbecoviruses were constructed as described[15] in two versions: one with a C-terminal 6x-His tag and a SpyTag003 (RGVPHIVMVDAYKRYK)[57] for the 8 RBDs that were coupled to SpyCatcher003-mi3 nanoparticles[57] and other versions with only a 6x-His tag or with a His tag plus an Avi tag for ELISAs. Expression vectors encoding RBDs were constructed similarly for the following sarbecoviruses: BM4831-CoV (GenBank: NC014470; spike residues 310-530), BtKY72-CoV (GenBank: KY352407; spike residues 309-530), C028 (GenBank: AAV98001.1; spike residues 306-523), Khosta2 (GenBank: QVN46569.1; spike residues 307-526), LYRa3 (GenBank: AHX37569.1; spike residues 310-527), Pangolin17-CoV (GenBank: QIA48632; spike residues 317-539), RaTG13-CoV (GenBank: QHR63300; spike residues 319-541), Rf1-CoV (GenBank: DQ412042; spike residues 310-515), RmYN02-CoV (GISAID: EPI_ISL_412977; spike residues 298-503), Rs4081-CoV (GenBank: KY417143; spike residues 310-515), RshSTT200 (GISAID: EPI_ISL_852605; spike residues 306-519), SARS-CoV (GenBank: AAP13441.1; spike residues 318-510), SARS-CoV-2 WA1 (GenBank: MT246667.1; spike residues 319-539), SHC014-CoV (GenBank: KC881005; spike residues 307-524), W1V1-CoV (GenBank: KF367457; spike residues 307-528), and Yun11-CoV (GenBank: JX993988; spike residues 310-515). SARS-CoV-2 variants with C-terminal 6x-His tags were also constructed similarly to the SARS-CoV-2 WA1 RBD construct for ELISAs. All RBD proteins were expressed by transient transfection of Expi293F cells and purified by Ni-NTA and size exclusion chromatography (SEC) using a HiLoad 16/600 Superdex 200 column (Cytiva).[12] Peak fractions were pooled, concentrated, and stored at 4°C until use.

SpyCatcher003-mi3[57] were expressed in *E. coli* BL21-CodonPlus (DE3)-RIPL (Agilent Technology) and purified as described previously.[15] Briefly, *E. coli* transduced with a SpyCatcher003-mi3 expression plasmid (Addgene) were lysed with a cell disrupter in the presence of 2 mM PMSF. After spinning at 21,000 x *g* for 30 minutes, supernatant containing SpyCatcher003-mi3 particles was passed over a pre-packed Ni-NTA column. The eluent was concentrated and further purified by SEC using a HiLoad 16/600 Superdex 200 column (Cytiva). Peak fractions were pooled and stored at 4°C until use. SpyCatcher003-mi3 particles were used for SpyTagged RBD conjugation for up to a month after clarification by filtering using a 0.2 μm filter or spinning at 21,000 x *g* for 10 min.

For conjugation, purified SpyCatcher003-mi3 was incubated with purified SpyTagged RBDs (either 8 different RBDs to make mosaic-8 RBD-mi3 or SARS-CoV-2 RBD only to make homotypic RBD-mi3) at a molar ratio of 1:1.2 overnight at room temperature. Conjugation efficiencies of individual RBDs to SpyCatcher003-mi3 were verified as shown in Figure S2 of Cohen et al.[15] Conjugated mi3-RBD particles were purified by SEC using a Superose 6 10/300 column (Cytiva). Peak fractions pooled and the concentrations of

conjugated mi3 particles were determined using a Bio-Rad Protein Assay (Bio-Rad). Conjugated nanoparticles were characterized by SEC, SDS-PAGE, and electron microscopy imaging as shown in Figures S1C–S1E, and by electron microscopy, SEC and dynamic light scattering previously.[12]

For negative-stain electron microscopy imaging of mosaic-8 and homotypic SARS-CoV-2 RBD-nanoparticles: ultrathin, holey carbon-coated, 400 mesh Cu grids (Ted Pella) were glow discharged (60 s at 15 mA), and a 3 μL aliquot of SEC-purified RBD-nanoparticles was diluted to ~40-100 μg/mL and applied to grids for 60 s. Grids were negatively stained with 2% (w/v) uranyl acetate for 30 s, and images were collected with a 120 keV FEI Tecnai T12 transmission electron microscope at 42,000x magnification.

Immunizations

Immunizations were done using protocols, #19023, approved by the City of Hope IACUC committee. Experiments were conducted using 4–6-week-old female C57BL/6 mice (Charles River Laboratories). Immunizations were carried out as previously described[15] using intraperitoneal injections of 5 μg of conjugated RBD-mi3 nanoparticle (calculated as the mass of the RBD, assuming 100% efficiency of conjugation to SpyCatcher003-mi3) in 100 μL of 50% v/v AddaVax™ adjuvant (Invivogen). Animals were boosted 4 weeks after the prime with the same quantity of antigen in adjuvant. A final booster was administered intraperitoneally 3 days before mouse spleen harvest.

Beacon

Plasma B cells were isolated from immunized animals for characterization on a Berkeley Lights Beacon instrument. Spleens were isolated from two immunized mice per condition and prepared into single cell suspensions as described.[15] Plasma B cells were isolated by $CD138^+$ cell enrichment (Miltenyi Biotec $CD138^+$ plasma cell isolation kit). Enriched plasma B cell samples were loaded onto an OptoSelect 11k chip (Berkeley Lights) in BLI Mouse Plasma Cell Media (Berkeley Lights). Single cells were then isolated in individual nanoliter-volume compartments (Nanopens using light-based OptoElectro Positioning (OEP) manipulation with settings optimized for plasma B cells. From Mosaic-8 RBD-nanoparticle immunized animals, 9,695 cells were penned in one chip, of which 7,747 were single cell pens. For homotypic SARS-CoV-2 RBD-nanoparticle immunized animals, 9,130 cells were penned in a second chip, of which 7,699 were single cell pens (Data S1). On chip fluorescence assays were used to identify cells secreting antibodies specific to RBD antigens. Briefly, C-terminally Avi-tagged RBDs were modified with site-specific biotinylation (Avidity) according to the manufacturer's protocol and immobilized on streptavidin-coated beads (Berkeley Lights). Assays were conducted by mixing beads coupled with one of four RBDs used for screening with a fluorescently labeled goat anti-mouse secondary antibody Alexa568 at 1:2500 dilution and importing this assay mixture into the OptoSelect 11k chip. Assays were conducted post 30 minutes incubation after cell penning at 36 °C. Images were acquired every 5 minutes for 9 cycles while the beads remained stationary in the main channel above the Nanopens of the OptoSelect chip. Antibodies specific for the immobilized RBD bound the antigen-coupled beads, which sequestered the fluorescent secondary antibody, creating a "bloom" of fluorescent signal immediately above Nanopens containing plasma B cells. Beads were washed out of the chip, and this assay was conducted for each of the four RBDs. After completion of all assays, RBD-specific cells of interest were exported using OEP from individual nanopen chambers to individual wells of a 96-well PCR plate containing lysis buffer.

After running assays and selecting positive blooms with single cells, we ran the OptoSeq BCR Export workflow, which performs reverse transcription overnight on the chip and exports cell lysates containing cDNA on capture beads onto a 96 well plate. cDNA amplification and chain-specific PCR were performed the following day and run on an agarose gel to confirm that bands of the correct size were present. PCR products were then purified using AMPure XP magnetic beads and submitted for Sanger sequencing at the City of Hope Sequencing Core.

Cloning

Sequences for V_H and V_L domains were codon optimized using GeneArt (Thermo Fisher Scientific) and gene blocks for each domain were purchased from Integrated DNA Technologies (IDT). Expression constructs were assembled using Gibson reactions.[58,59] The heavy chain for IgG expression was constructed by subcloning the V_H gene into a p3BNC expression vector encoding the human IgG C_H1, C_H2, and C_H3 domains, and the heavy chain for Fab expression was constructed by assembling the V_H gene into a p3BNC expression vector encoding a human C_H1 and a C-terminal 6x-His tag. The expression plasmid for the light chain was constructed by subcloning the V_L gene into a p3BNC vector that also encoded kappa human C_L. The numbering of V_H and V_L protein sequences and the identification of the V gene segments were determined using the ANARCI server.[60]

IgG and spike trimer production and purification

Proteins were expressed in Expi293 cells by transient transfection. IgGs and a previously described human ACE2-Fc construct[11] were purified from cell supernatants using MabSelect SURE columns (Cytiva), and His-tagged Fabs were isolated from cell supernatants using Ni-NTA columns (Qiagen). IgGs, ACE2-Fc, and Fabs were further purified by SEC using a HiLoad 16/600 Superdex 200 column (Cytiva). Purified proteins were concentrated using a 100 kDa and 30 kDa cutoff concentrator (EMD Millipore), respectively, to 10 to 15 mg/mL, and final concentrated proteins were stored at 4 °C until use. 6P versions[25] of soluble SARS-CoV-2 WA1 and SARS-CoV-2 Omicron BA.1 spike trimers were isolated from cell supernatants using a pre-packed Ni-NTA column (Cytiva). Eluents from Ni-NTA purifications were subjected to SEC using a HiLoad Superdex 200 16/600 column followed by a Superose 6 10/300 (Cytiva) column. Peak fractions were pooled and concentrated to ~6 mg/ml, flash frozen in 50 μL aliquots, and stored at -80 °C until use.

ELISAs

Nunc® MaxiSorp™ 384-well plates (Millipore Sigma) were coated with 10 μg/mL of purified RBD in 0.1 M $NaHCO_3$ pH 9.8 and stored overnight at 4 °C. After blocking with 3% bovine serum albumin (BSA) for an hour at room temperature, plates were washed with Tris-buffered saline including 0.1% Tween 20 (TBST). After removing blocking solution from the plates, 100 μg/mL of purified IgGs were serially diluted by 4-fold using TBST with 3% BSA and incubated with plates at room temperature for 3 hours. Plates were then washed with TBST and incubated with secondary HRP-conjugated goat anti-human IgG (SouthernBiotech) at a 1:15,000 dilution for 45 minutes at room temperature. Plates were washed with TBST, developed using SuperSignal ELISA Femto Maximum Sensitivity Substrate (Thermo Fisher Scientific), and read at 425 nm. ELISA data were collected in duplicate, and each assay was conducted at least twice for the seven mAbs that were structurally characterized. Curves were plotted and integrated to obtain half-maximal effective concentrations (EC_{50}) using Graphpad Prism v9.3.1 assuming a one-site binding model with a Hill coefficient.

Competition ELISAs were performed using a Tecan Evo liquid handling robot using modifications of a previously described protocol.[61] IgGs were randomly biotinylated at primary amines using EZ-link NHS-PEG4 Biotinylation Kit according to the manufacturer's protocol (Thermo Fisher Scientific). SARS-CoV-2 RBD (2.5 μg/mL) was adsorbed overnight at 4°C to a 384-well Nunc MaxiSorp ELISA plate (Millipore Sigma). The RBD was removed via aspiration and the plate blocked with 3% BSA in TBST for 1 hour at room temperature. The blocking was removed via aspiration and 10 μg/mL unlabeled IgG was added and incubated for 2 hours, followed by addition of 0.25 μg/mL biotinylated IgG. The plate was incubated for 2 hours at room temperature, and bound biotinylated IgG was detected using horseradish peroxidase-conjugated streptavidin (SouthernBiotech) (1 hour, room temperature) and developed with SuperSignal ELISA Femto Substrate (Thermo Fisher Scientific). Relative light units (RLU) were measured and the signal for each competition pair was normalized to the signal for the biotinylated IgG when unlabeled IgG was not present. Measurements were performed in technical quadruplicates. Data presented are representative of two independent experiments.

Pseudovirus neutralization assays

SARS-CoV-2, SARS-CoV-2 VOCs, SARS-CoV, WIV1, SHC014, BtKY72 (including mutations allowing human ACE2 receptor binding,[16] Khosta2/SARS-CoV, and LYRa3/SARS-CoV chimera pseudoviruses based on HIV lentiviral particles were prepared as described.[17,21] Khosta2/SARS-CoV and LYRa3/SARS-CoV chimeric spikes were constructed by replacing the RBD of the SARS-CoV spike with the RBD of either Khosta2 and LYRa3 spike as described.[12] Assays were done using 4-fold dilutions of purified IgGs at a starting concentration of 100 μg/mL by incubating with a pseudovirus at 37 °C for an hour. After incubating with $293T_{ACE2}$ target cells for 48 hours at 37 °C, cells were washed 2 times with phosphate-buffered saline (PBS) and lysed with Luciferase Cell Culture Lysis 5x reagent (Promega). Using the Nano-Glo Luciferase Assay System (Promega), the NanoLuc Luciferase activity in lysates was measured. Relative luminescence units (RLUs) were normalized to values derived from cells infected with pseudovirus in the absence of IgG. Data were collected at each IgG concentration in duplicate and reported data come from assays performed at least twice. Half-maximal inhibitory concentrations (IC_{50} values) in Figure 2B were determined using nonlinear regression in AntibodyDatabase.[62] Differences between neutralization titers were evaluated for statistical significance between mAbs using analysis of variance (ANOVA) followed by Tukey's multiple comparison with Graphpad Prism v9.3.1. Significant differences calculated using Tukey's multiple comparison test between mAbs linked by horizontal lines are indicated by asterisks: $*p < 0.05$, $**p < 0.01$, $***p < 0.001$, $****p < 0.0001$.

X-ray crystallography

RBD-Fab complexes were formed by incubating SARS-CoV-2 RBD with a 1.1x molar excess of Fab for an hour at room temperature. Complexes were purified by SEC using a Superdex 200 10/300 Increase column (Cytiva). Peak fractions containing RBD-Fab complexes were pooled and concentrated to ~15 mg/ml. Crystallization trials were set up using commercially available screens by mixing 0.2 μL of RBD-Fab complex and 0.2 μL well solution using a TTP LabTech Mosquito instrument via the sitting drop vapor diffusion method at room temperature. Crystals of M8a-6 Fab–RBD complex were obtained from Proplex screen (Molecular Dimensions), containing 0.1 M sodium citrate pH 5.5 and 15 % PEG 6,000. Crystals of M8a-34 Fab–RBD complex were obtained from a PEGion screen (Hampton Research), containing 2% v/v tacsimate pH 4.0, 0.1 M sodium acetate trihydrate pH 4.6, 16 % PEG 3,350. Crystals of RBD–HSW-2 complexes were obtained from a Proplex screen (Molecular Dimensions), containing 0.2 M sodium chloride, 0.1 M sodium/potassium phosphate pH 6.5, 25 % PEG 1,000. All crystals were cryoprotected in well solution mixed with 20% glycerol or PEG 400 before freezing in liquid nitrogen.

X-ray diffraction data were collected at the Stanford Synchrotron Radiation Lightsource (SSRL) beamline 12-2 with a Pilatus 6M pixel detector (Dectris) using the Blu-ice interface[63] (Table S3). All X-ray datasets were indexed and integrated with XDS[49] and scaled with Aimless.[53] The M8a-6 Fab–RBD structure was solved by molecular replacement using a structure of a Fab-RBD complex from a single-particle cryo-EM structure (PDB 7SC1) as the input model for *Phaser* in Phenix.[51] During the refinement of the M8a-6 Fab–RBD structure, we observed electron density for a second RBD and the variable domains of M8a-6 Fab, but no Fab constant domains were found. Refinement of a model containing the original M8a-6 Fab–RBD complex, a second copy of RBD and the variable domains resulted in no improvements in the refinement statistics. We thus only partially refined the coordinates for the M8a-6 Fab–RBD crystal structure, which were then docked and refined in the cryo-EM M8a-6–spike reconstruction. The M8a-34 Fab–RBD structure was solved by molecular replacement using the partially refined model of M8a-6–RBD complex structure as the input model for *Phaser* in Phenix.[51] The HSW-2 Fab–RBD structure was solved by molecular replacement using the partially refined model of M8a-34–RBD complex structure as the input model for *Phaser* in Phenix.[51] Iterative refinement and model-building cycles were carried out with

phenix.refine in Phenix[51] and *Coot*,[52] respectively. The refined models were subsequently used as input models for docking into cryo-EM maps of Fab-spike complexes.

Cryo-EM sample preparation

SARS-CoV-2 S–Fab complexes were formed by incubating purified spike trimer and Fabs at a 1.1x molar excess of Fab per spike protomer at room temperature for 30 minutes to a final concentration of ~2 mg/mL. Fluorinated octylmaltoside solution (Anatrace) was added to the spike-Fab complex to a final concentration of 0.02% (w/v) prior to freezing, and 3 μL of the complex/detergent mixture was immediately applied to QuantiFoil 300 mesh 1.2/1.3 grids (Electron Microscopy Sciences) that had been freshly glow discharged with PELCO easiGLOW (Ted Pella) for 1 min at 20 mA. Grids were blotted for 3 to 4 seconds with 0 blot force using Whatman No.1 filter paper and 100% humidity at room temperature and vitrified in 100% liquid ethane using a Mark IV Vitrobot (Thermo Fisher Scientific).

Cryo-EM data collection and processing

Single-particle cryo-EM datasets for complexes of SARS-CoV-2 WA1 spike 6P with M8a-3 Fab, M8a-6 Fab, M8a-28 Fab, M8a-31 Fab, M8a-34 Fab or HSW-1 Fab and SARS-CoV-2 Omicron BA.1 spike 6P with M8a-31 Fab were collected using SerialEM automated data collection software[43] on a 300 keV Titan Krios (Thermo Fisher Scientific) cryo-electron microscope equipped with a K3 direct electron detector camera (Gatan). For SARS-CoV-2 WA1 spike 6P complexed with HSW-2, a dataset was collected with SerialEM[43] on a 200 keV Talos Arctica cryo-electron microscope (Thermo Fisher Scientific) equipped with a K3 camera (Gatan). Movies were recorded with 40 frames, a defocus range of -1 to -3 μm, and a total dosage of 60 e^{-}/Å^2 using a 3x3 beam image shift pattern with 3 exposures per hole in the superresolution mode with a pixel size of 0.416 Å for the collections on the Krios and a single exposure per hole in the superresolution mode with a pixel size of 0.4345 Å for the collection on the Talos Arctica. Detailed data processing workflows for each complex structure are outlined in Data S1. All datasets were motion corrected with patch motion correction using a bining factor of 2, and CTF parameters were estimated using Patch CTF in cryoSPARC v3.2.[44] Particle picking was done with blob picker in cryoSPARC using a particle diameter of 100 to 200 Å, and movies and picked particles were inspected before extraction. Particles were extracted and classified using 2D classification in cryoSPARC.[44] After discarding ice and junk particles, the remaining particles were used for *ab initio* modeling with 4 volumes, which were futher refined with heterogeneous refinement in cryoSPARC.[44] Subsequent homogeneous and non-uniform refinements were carried out for final reconstructions in cryoSPARC.[44] Because Fab interactions with ‘up’ RBDs are generally not well resolved in Fab-spike complex structures,[18] we used masks to locally refine and improve the interfaces of Fabs bound to ‘up’ RBDs when necessary. For local refinements, masks were generated using UCSF Chimera[45] and refinements were carried out in cryoSPARC.[44]

Cryo-EM structure modeling and refinement

An initial model of the M8a-3 Fab–spike trimer complex was generated by docking a single-particle cryo-EM Fab-SARS-CoV-2 spike 6P complex structure (PDB 7SC1) into the cryo-EM density using UCSF Chimera.[45] The model was refined using real space refinement in Phenix.[51] The Fab amino acid seqence was manually corrected in *Coot*.[52] The model of the M8a-3 Fab–spike complex was subsequently used for docking and model generation for remaining Fab-spike trimer complexes. For the Fab-spike complexes that we have RBD-Fab crystal structures for (M8a-6 Fab-RBD, M8a-34 Fab–RBD and HSW-2 Fab–RBD structures), we first docked the spike trimer (PDB 7SC1) in the EM density map, manually fitted the RBDs in *Coot*[52] and refined the spike trimer using phenix.real_space_refine.[51] The RBD-Fab structures were then aligned to each of the RBDs in the corresponding Fab–spike complexes, and the RBD regions in the EM model were replaced by the RBDs from crystal structures upon structural alignments in *Coot*.[52] The final model containing the spike trimer and the Fabs were subsequently refined with phenix.real_space_refine.[51] Iterative real space refinement and model building were separately carried out in Phenix[51] and *Coot*.[52] Single-particle cryo-EM refinement statistics are reported in Table S2.

Structure analyses

Structure figures were made using UCSF ChimeraX.[47,48] Distances were measured using PyMOL v2.4.0.[55] Interacting residues between a Fab and RBD were analyzed by PDBePISA[28] using the following interaction definitions: potential H bonds were defined as a distance less than 3.9 Å between the donor and acceptor residues when H was present at the acceptor and there was an A-D-H angle between 90° and 270°; potential salt bridges were defined between residues that were less than 4 Å. Sequence alignments were done using Geneious (https://www.geneious.com/). Buried surface areas (BSAs) were calculated by PDBePISA using a 1.4 Å diameter probe.[28]

To evaluate the potential for intra-spike crosslinking by the two Fabs of a single IgG binding to adjacent RBDs within a single spike trimer, we measured the distances between the Cα atoms of the C-terminal residues of the C_H1 domains of adjacent RBD-binding Fabs in the structures of mAb-spike complexes as described previously.[10] A cut-off of no more than 65 Å was used to identify IgGs whose binding orientation could allow for both Fabs to bind simultaneously to adjacent RBDs in a single spike trimer. This cut-off was larger than the distance measured between comparable residues of C_H1 domains in intact IgG crystal structures (42Å, PDB: 1HZH; 48Å, PDB: 1IGY; 52Å, PDB: 1IGT) to account for potential influences of crystal packing, flexibilities in the elbow bend angle relating the V_H-V_L and C_H1-C_L, and uncertainties in the placements of C_H1-C_L domains in cryo-EM structures of the Fab-spike complexes.[10]

SPR assays

SPR experiments were done using a Bruker Sierra SPR-32 Pro instrument (Bruker). Protein A was immobilized on a High Capacity Amine chip by primary amine chemistry to ~3,000 response units (RUs). C118, M8a-3, and M8a-6 IgGs were captured by Protein A and were used as the ligands. The eight RBDs listed in Data S1 were used as analytes and were prepared in concentration series of 11 threefold dilutions from a top concentration of 10,000 nM. Analytes were injected at a flow rate of 30 μL/min over immobilized IgGs for 60 s, followed by a dissociation phase injection of 1x HBS-EP^{+} buffer for 300 s. K_D values were calculated from the ratio of association and dissociation rates ($K_D = k_d / k_a$) derived from a 1:1 binding model for sensorgrams in which kinetic constants are listed in Data S1. Kinetic constants were calculated using Bruker Sierra SPR-32 Pro analysis software with a global fit to experimental curves indicated with model fits (black lines) in Data S1. For binding sensorgrams that reached or approached equilibrium (two of the M8a-3 and all of the M8a-6 sensorgrams), we derived K_D values from the midpoints of plots of RU_{max} versus concentration fit to a 1:1 binding model; thus, kinetic constants are not listed for these sensorgrams in Data S1. As recommended for SPR data analysis,[64,65] we did not derive kinetic and/or equilibrium constants for data sets that could not be fit to a biologically-relevant binding model (a 1:1 binding model in this case). Flow cells were regenerated with 10 mM glycine, pH 2.0, at a flow rate of 30 μL/min.

QUANTIFICATION AND STATISTICAL ANALYSIS

For ELISAs shown in Figure 2A, half-maximal effective concentrations (EC_{50} values) were obtained by plotting concentrations versus relative light units (RLUs) and fitting to a sigmoidal curve by assuming a one-site binding model with a Hill coefficient using Graphpad Prism v9.3.1. Half-maximal inhibitory concentrations (IC_{50} values) in Figure 2B were obtained using nonlinear regression in AntiboдyDatabase.[62] Differences between neutralization titers were evaluated for statistical significance between mAbs using analysis of variance (ANOVA) followed by Tukey's multiple comparison with Graphpad Prism v9.3.1. Significant differences calculated using Tukey's multiple comparison test between mAbs linked by horizontal lines are indicated by asterisks: *$p < 0.05$, **$p < 0.01$, ***$p < 0.001$, ****$p < 0.0001$. Structures determined by X-ray crystallography are objectively evaluated using statistical criteria[66] that are required when depositing coordinates in the Protein Data Bank (PDB). The PDB validation report compares coordinate geometry and refinement statistics for a new structure to others at the same resolution, thus ensuring that poorly refined or incorrect structures are flagged. For cryo-EM structures, we deposit maps in the Electron Microscopy Data Bank (EMDB) and coordinates in the PDB, following recommendations to avoid over-fitting[67] and model bias influences.[68]

Immunity, Volume *55*

Supplemental information

Neutralizing monoclonal antibodies elicited by mosaic RBD nanoparticles bind conserved sarbecovirus epitopes

Chengcheng Fan, Alexander A. Cohen, Miso Park, Alfur Fu-Hsin Hung, Jennifer R. Keeffe, Priyanthi N.P. Gnanapragasam, Yu E. Lee, Han Gao, Leesa M. Kakutani, Ziyan Wu, Harry Kleanthous, Kathryn E. Malecek, John C. Williams, and Pamela J. Bjorkman

Supplemental figures

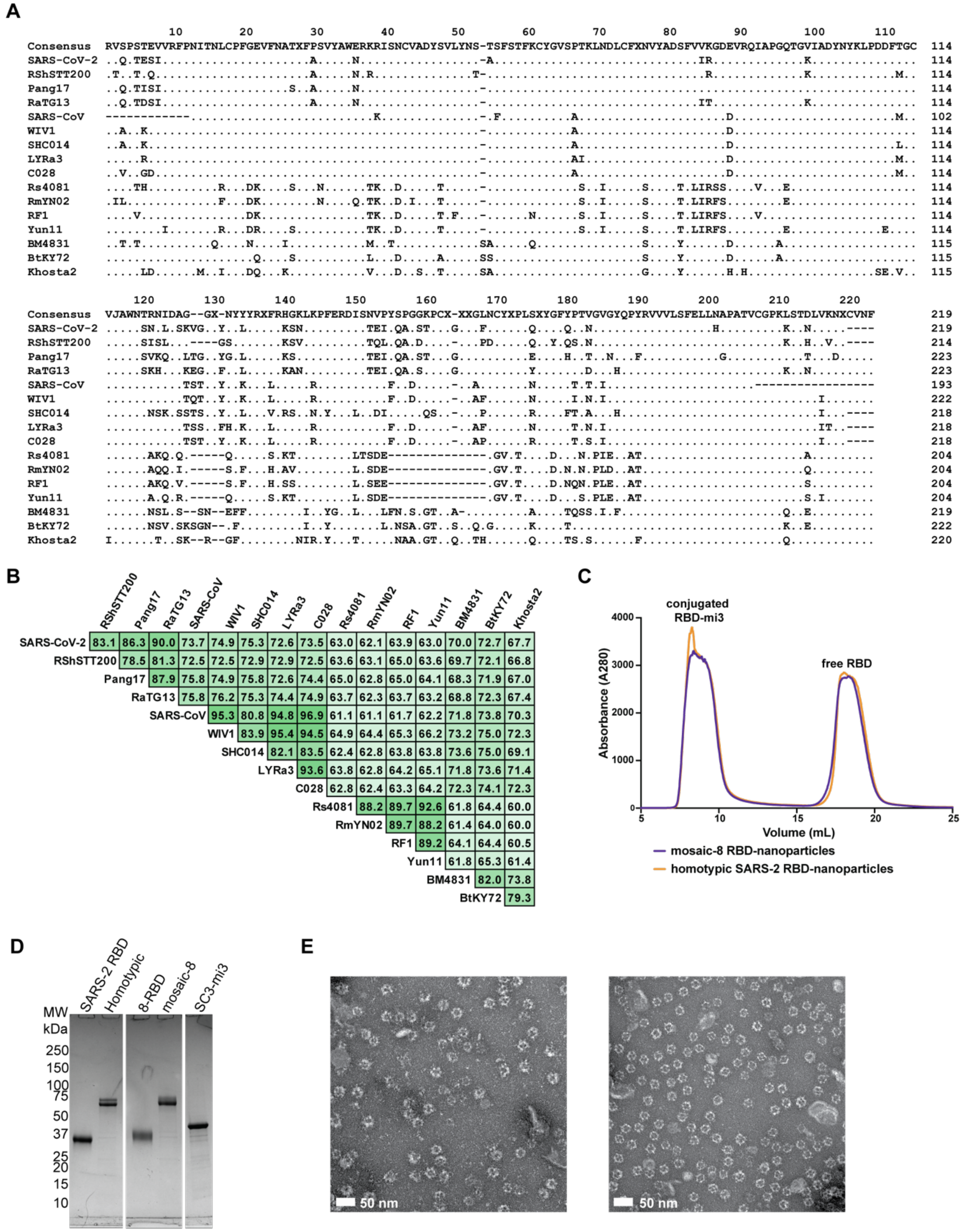

Figure S1. Sarbecovirus RBDs and construction of RBD-nanoparticles. Related to Figure 1.

(**A**) Sequence alignment of RBDs from 16 sarbecoviruses. (**B**) Pairwise sequence identities (%) relating the RBDs in panel A. (**C**) SEC profile showing separation of conjugated SpyCatcher003-mi3 nanoparticles from free RBDs. RBDs were added at a 2-fold molar excess over SpyCatcher003-mi3 subunits. (**D**) Reducing SDS-PAGE analysis of free RBD, purified conjugated RBD-nanoparticles, and unconjugated SpyCatcher-mi3 nanoparticles. (**E**) Negative stain EM of conjugated nanoparticles. Left: mosaic-8 RBD-nanoparticles. Right: homotypic SARS-CoV-2 RBD-nanoparticles.

A

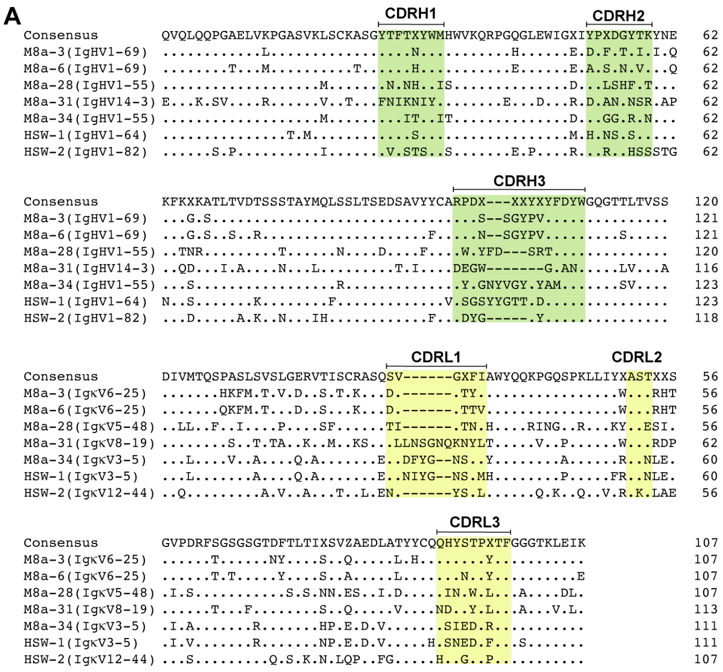

B

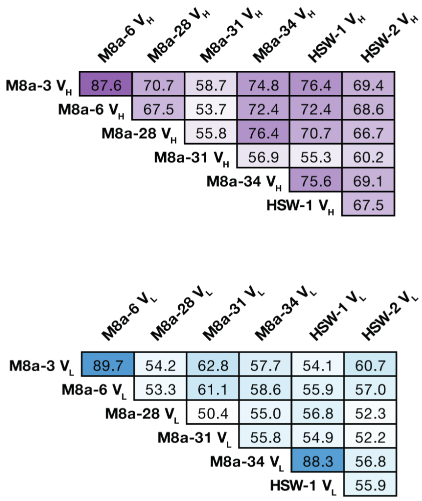

C

	CDRH1	CDRH2	CDRH3	CDRL1	CDRL2	CDRL3
**M8a-3**	8	8	14	6	3	9
**M8a-6**	8	8	14	6	3	9
**M8a-28**	8	8	13	6	3	9
**M8a-31**	8	8	9	12	3	9
**M8a-34**	8	8	16	10	3	9
**HSW-1**	8	8	16	10	3	9
**HSW-2**	8	8	11	6	3	9

D

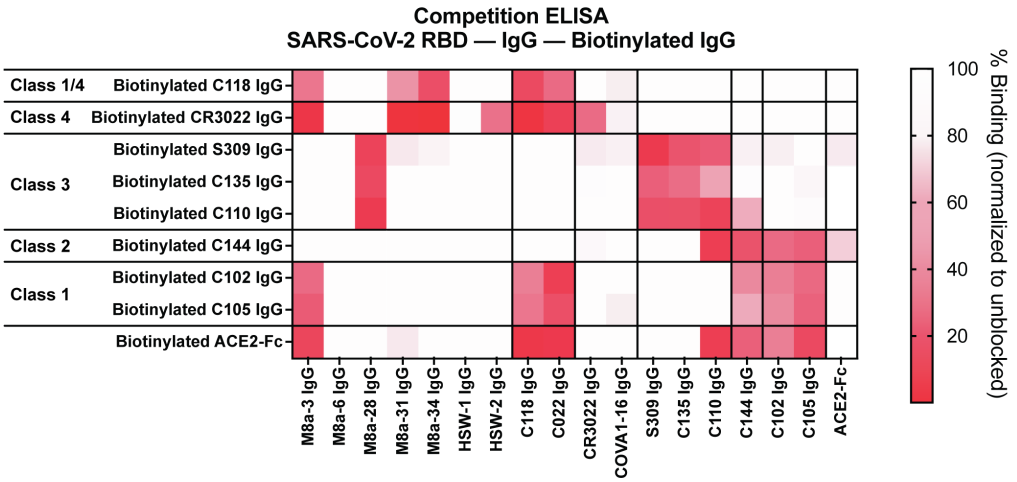

Figure S2. Sequence alignment of seven mAbs isolated from RBD nanoparticle-immunized mice that bound two or more sarbecovirus RBDs. Related to Figure 2.

(**A**) Sequence alignment of V_H and V_L domains. CDRs were assigned using IMGT definitions [S3]. VH and VL gene segment assignments for each mAb were shown in parentheses. (**B**) Pairwise

sequence identities (%) between V_H and V_L domains in (**A**). (**C**) Number of residues in CDRs. (**D**) Competition ELISA experiment in which a test set of biotinylated IgGs of known epitopes or human ACE2-Fc (y-axis) were assayed for binding to SARS-CoV-2 RBD in the presence of unlabeled M8a, HSW, control IgGs, or ACE2-Fc (x-axis). The heat map shows shades of red indicating the percent binding of the tested IgGs or ACE2-Fc in the presence of competitor. M8a-3, M8a-31, M8a-34, and HSW-2 IgGs competed with biotinylated class 1/4 and/or class 4 anti-RBD, with HSW-2 competing with the biotinylated class 4 antibody only. M8a-3 IgG competed with biotinylated class 1 antibodies and ACE2-Fc in addition to the biotinylated class 1/4 and class 4 antibodies. M8a-28 IgG competed with biotinylated class 3 antibodies. M8a-6 and HSW-1 IgGs showed no competition in this assay.

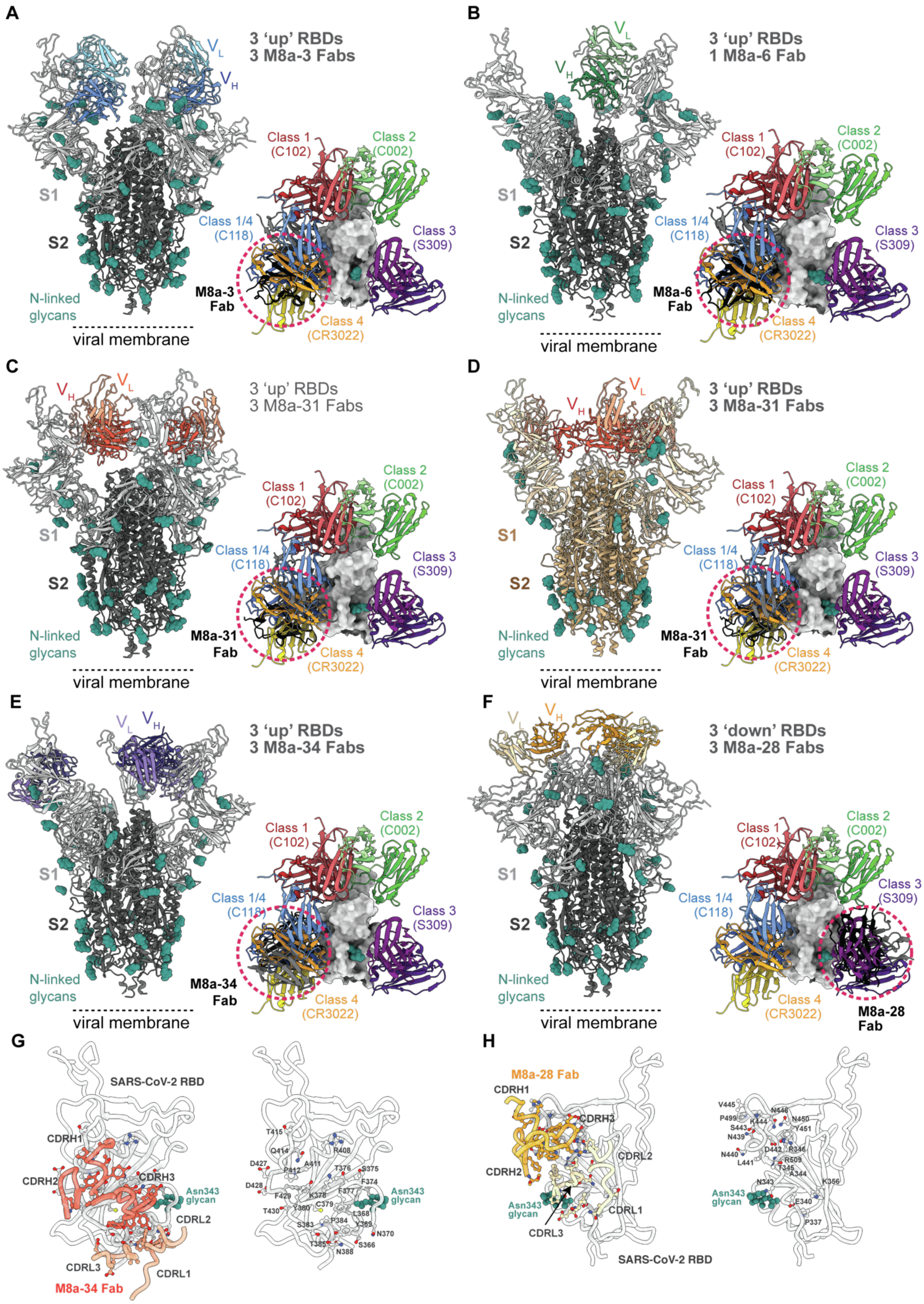

A
3 'up' RBDs
3 M8a-3 Fabs
VL
VH
S1
S2
N-linked glycans
viral membrane
Class 1 (C102)
Class 2 (C002)
Class 1/4 (C118)
Class 3 (S309)
M8a-3 Fab
Class 4 (CR3022)
B
3 'up' RBDs
1 M8a-6 Fab
M8a-6 Fab
C
3 'up' RBDs
3 M8a-31 Fabs
M8a-31 Fab
D
3 'up' RBDs
3 M8a-31 Fabs
E
3 'up' RBDs
3 M8a-34 Fabs
M8a-34 Fab
F
3 'down' RBDs
3 M8a-28 Fabs
M8a-28 Fab
G
SARS-CoV-2 RBD
CDRH1
CDRH2
CDRH3
Asn343 glycan
CDRL2
CDRL3
CDRL1
M8a-34 Fab
T415
Q414
R408
D427
A411
T376
S375
P412
F374
D428
F429
K378
F377
T430
Y380
C379
S383
P384
L368
Y369
N370
T385
S366
N388
H
M8a-28 Fab
CDRH1
CDRH3
CDRL2
CDRH2
Asn343 glycan
CDRL1
CDRL3
SARS-CoV-2 RBD
V445
N448
P499
K444
N450
S443
Y451
N439
D442
R346
N440
L441
R509
T345
A344
N343
K356
E340
P337

Figure S3. Structural analysis of mAbs isolated from mice immunized with mosaic-8 nanoparticles. Related to Figure 3.

Cartoon representations of single particle cryo-EM structures of Fab-spike trimer complexes are shown from the side (left) with a comparison of binding epitopes of the Fab with representative anti-RBD antibodies: class 1 (C102, PDB 7K8M), class 2 (C002, PDB 7K8T), class 3 (S309, PDB 7JX3), class 4 (CR3022, PDB 7LOP), and class 1/4 (C118, PDB 7RKV)) aligned on an RBD in surface representation (right). Only V_H-V_L domains are shown for each Fab. Fabs of interests (colored in black and circled with a red dotted line) and the anti-RBD antibodies used for classification are aligned on a surface representation of the RBD. N-linked glycans are shown as teal spheres. (**A**) WA1 spike complexed with M8a-3. (**B**) SARS-CoV-2 WA1 spike complexed with M8a-6. (**C**) WA1 spike complexed with M8a-31. (**D**) Omicron BA.1 spike complexed with M8a-31. (**E**) WA1 spike complexed with M8a-34. (**F**) WA1 spike complexed with M8a-28. Left: interactions between SARS-CoV-2 RBD and the CDRs of (**G**) M8a-28 Fab and (**H**) M8a-34 Fab. Right: SARS-CoV-2 RBD residues involved in interactions with (**G**) M8a-28 Fab and (**H**) M8a-34 Fab.

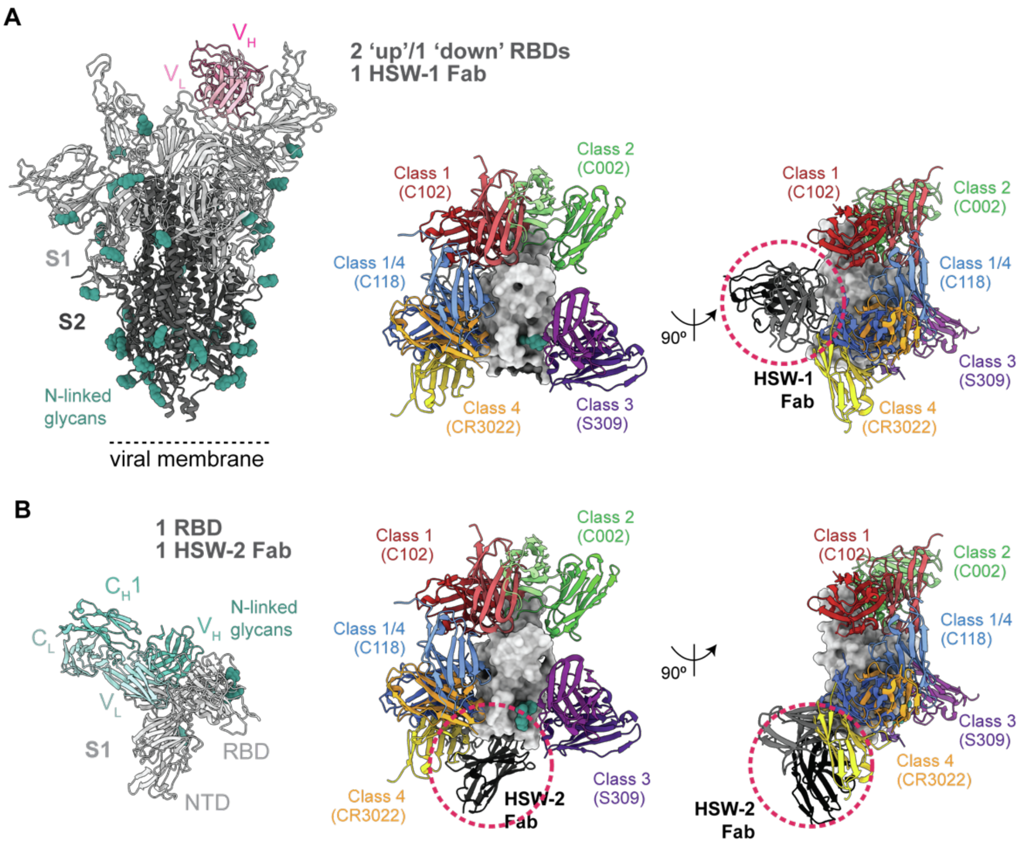

Figure S4. Structural analysis of mAbs isolated from mice immunized with homotypic nanoparticles. Related to Figure 4.

Cartoon representations of single particle cryo-EM structures of Fab-spike trimer complexes are shown from the side (left) with a comparison of binding epitopes of the Fab with representative anti-RBD antibodies: class 1 (C102, PDB 7K8M), class 2 (C002, PDB 7K8T), class 3 (S309, PDB 7JX3), class 4 (CR3022, PDB 7LOP) and class 1/4 (C118, PDB 7RKV)) aligned on an RBD in surface representation (middle and right). Only V_H-V_L domains are shown for each Fab. Fabs of interests (colored in black and circled with a red dotted line) and the anti-RBD antibodies used for classification are aligned on a surface representation of the RBD. N-linked glycans are shown as teal spheres. (**A**) SARS-CoV-2 WA1 spike complexed with HSW-1. (**B**) WA1 spike S1 domain complexed with HSW-2.

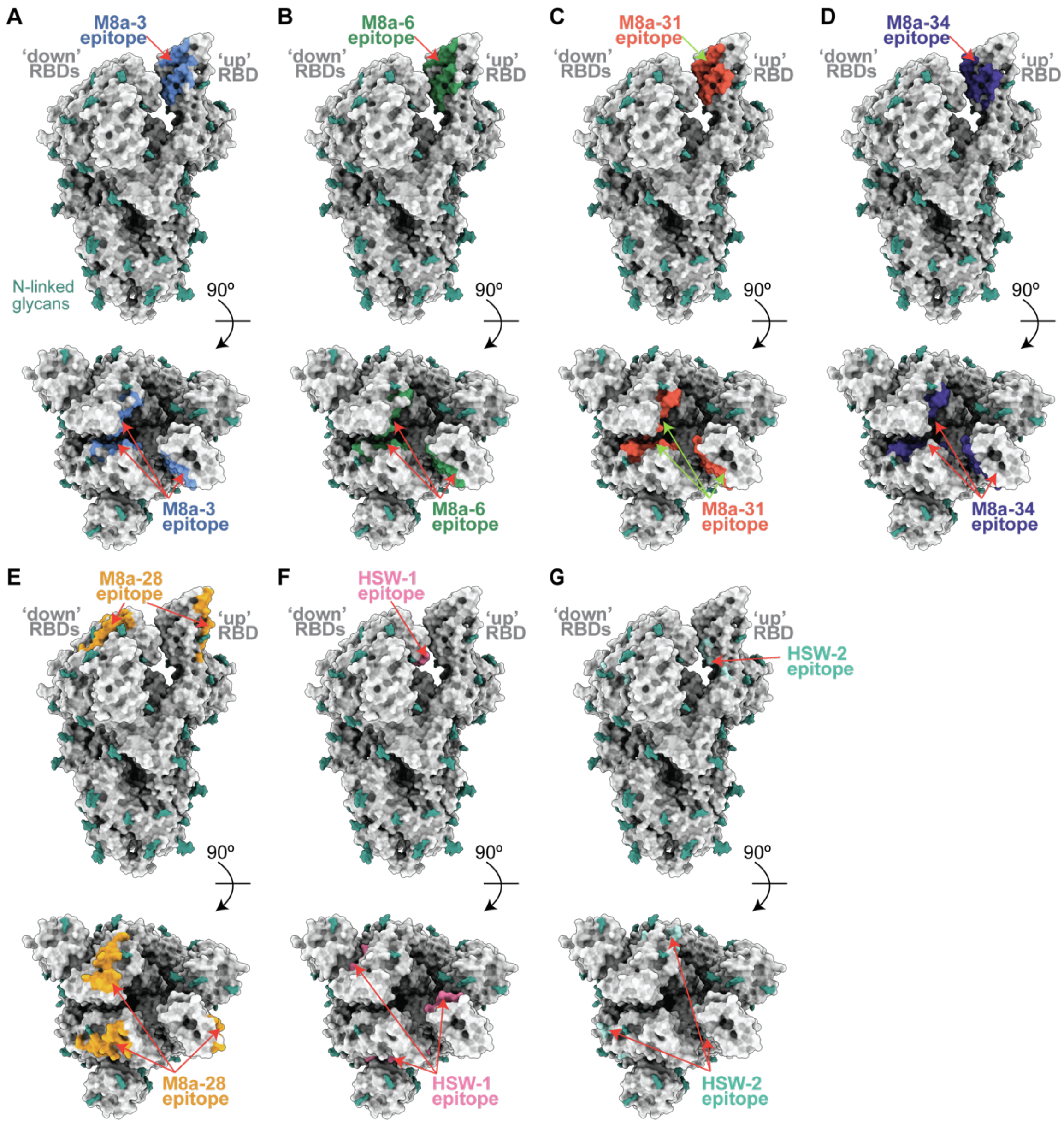

Figure S5. Epitopes of mAbs mapped on a unliganded SARS-CoV-2 spike trimer. Related to Figure 5 and 6.

Binding epitopes for all mAbs were identified by PDBePISA [S2] and then mapped on to a unliganded spike trimer with two 'down' and one 'up' RBDs (PDB 6VYB). The spike trimer is shown as a surface representation with N-linked glycans shown as teal spheres. The epitopes of (**A**) M8a-3, (**B**) M8a-6, (**C**) M8a-31 and (**D**) M8a-34 are blocked in the 'down' RBD conformation, but accessible in an 'up' RBD conformation. (**E**) The epitope of M8a-28 is accessible in both 'down' and 'up' RBD conformations. (**F**) The epitope of HSW-1 is blocked in the 'down' RBD

conformation, but accessible in an ‘up’ RBD conformation. (**G**) The epitope of HSW-2 is blocked in both ‘down’ and ‘up’ RBD conformations.

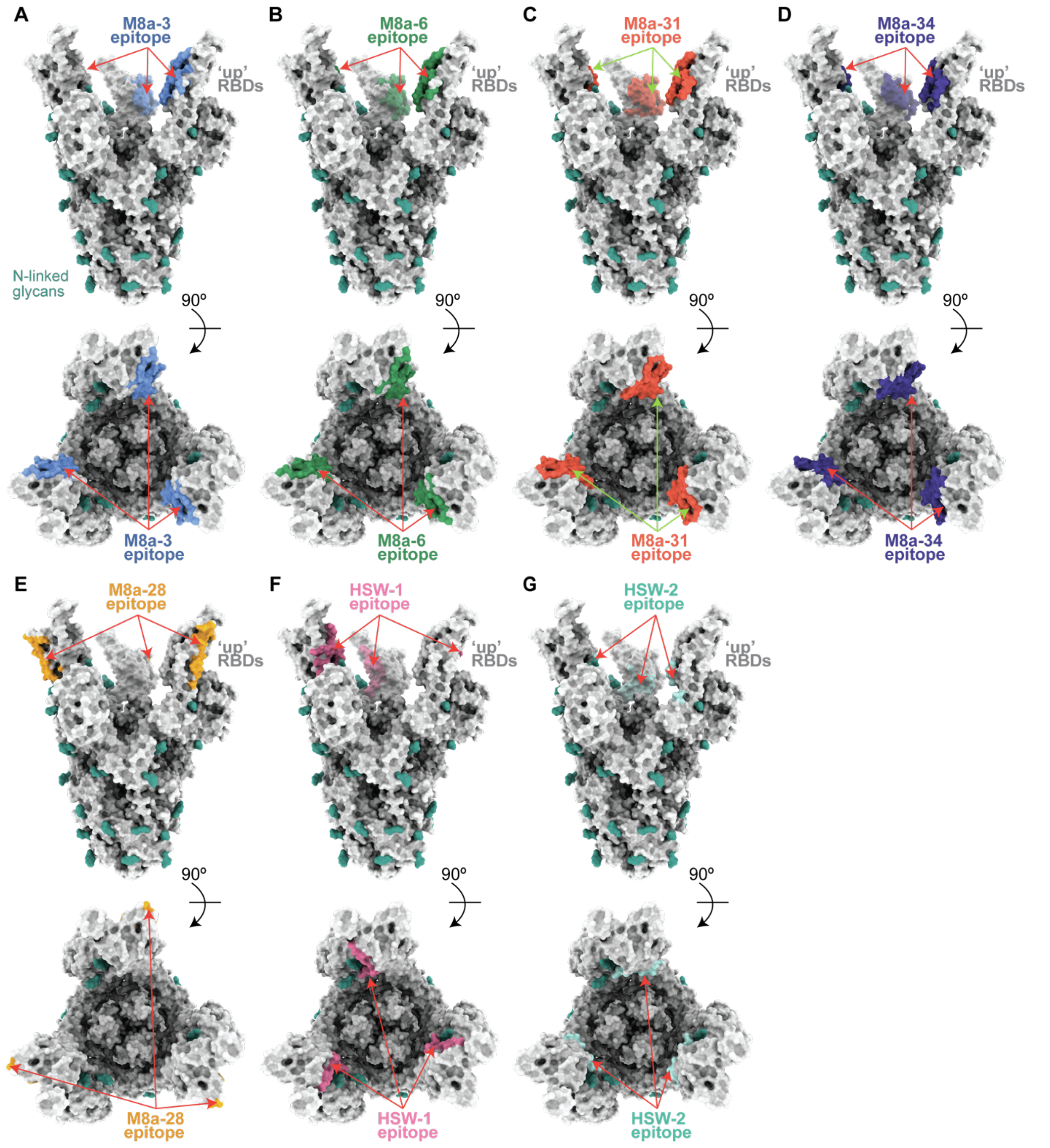

Figure S6. Epitopes of mAbs mapped on a SARS-CoV-2 spike trimer with all 'up' RBDs. Related to Figure 5 and 6.

Binding epitopes for all mAbs were identified by PDBePISA [S2] and then mapped on to a spike trimer with three 'up' RBDs (PDB 7RKV; Fabs not shown). The spike trimer is shown as a surface representation with N-linked glycans shown as teal spheres. The epitopes of (**A**) M8a-3, (**B**) M8a-6, (**C**) M8a-31, and (**D**) M8a-34 are all accessible in an 'up' RBD conformation. (**E**) The epitope

of M8a-28 is accessible in ‘up’ RBD conformation. (**F**) The epitope of HSW-1 is accessible in an ‘up’ RBD conformation. (**G**) The epitope of HSW-2 is sterically hindered in an ‘up’ RBD conformation.

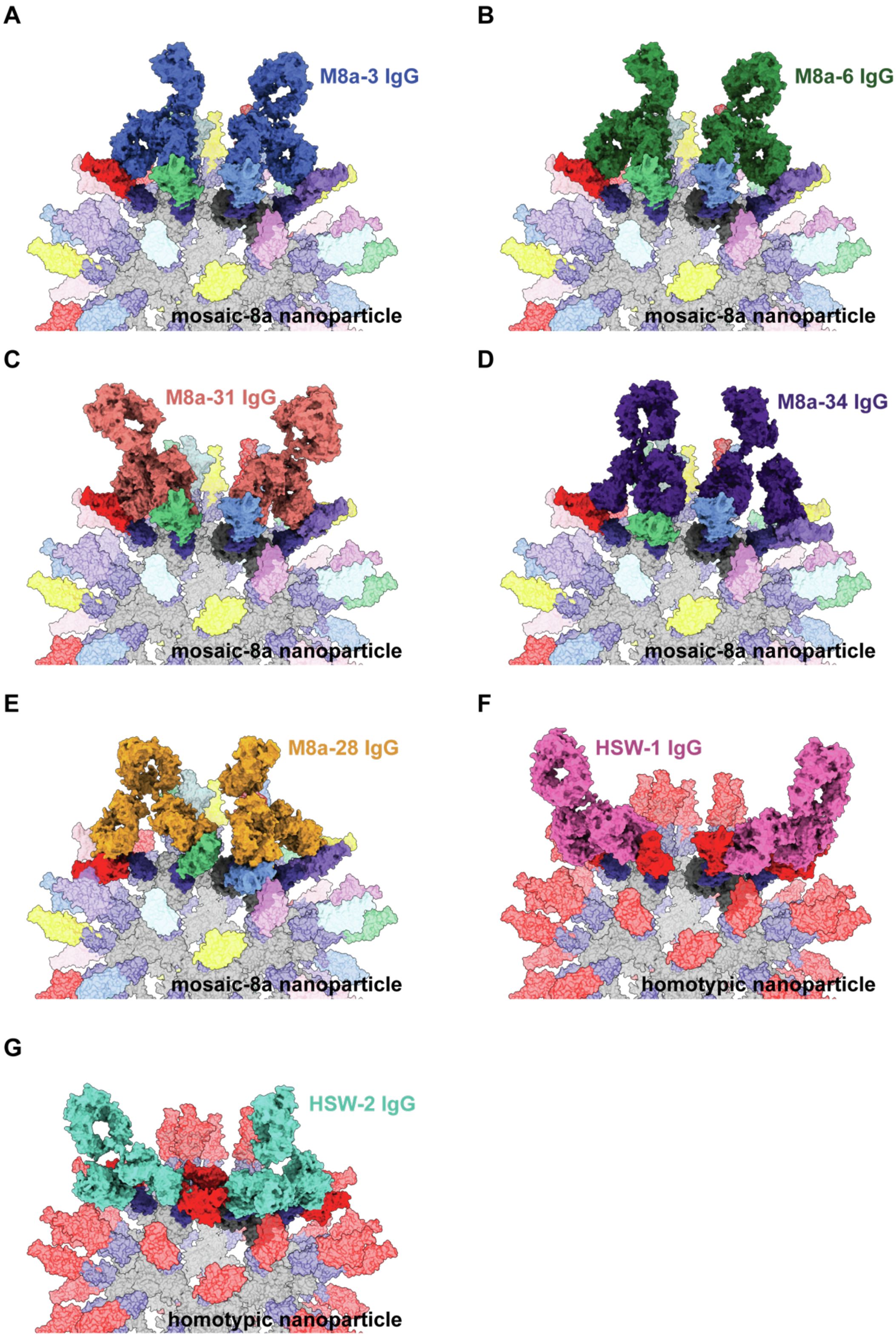

Figure S7. Models of M8a and HSW IgGs binding to adjacent RBDs on SpyCatcher-mi3 nanoparticles. Related to Figure 6.

Structural models of RBD-nanoparticles formed by SpyCatcher-mi3 and SpyTagged RBDs were made using coordinates of an RBD (PDB 7SC1) (represented in different colors for mosaic-8 nanoparticles, with two adjacent RBDs in red and blue, or in red for homotypic nanoparticles), mi3 (PDB 7B3Y) (gray), and SpyCatcher (PDB 4MLI) (dark blue). IgGs (various colors) were modeled using the coordinates of each mAb Fab based on an intact IgG crystal structure (PDB 1HZH), and the RBD binding epitope of each Fab in a modeled IgGs was determined based on the Fab-spike structures reported in this study. The two Fabs of each IgGs were positioned with distances between the C-termini of the Fab C_H1 domains to be less than 65 Å, as described previously [S1]. Both the IgG Fc hinge and the linker region between a SpyTagged RBD and SpyCatcher were assumed to be flexible and adjusted accordingly. Models are shown for (**A**) M8a-3 IgG interacting with adjacent RBDs on a mosaic-8 RBD-nanoparticle. (**B**) M8a-6 IgG interacting with adjacent RBDs on a mosaic-8 RBD-nanoparticle. (**C**) M8a-31 IgG interacting with adjacent RBDs on a mosaic-8 RBD-nanoparticle. (**D**) M8a-34 IgG interacting with adjacent RBDs on a mosaic-8 RBD-nanoparticle. (**E**) M8a-28 IgG interacting with adjacent RBDs on a mosaic-8 RBD-nanoparticle. (**F**) HSW-1 IgG interacting with adjacent RBDs on a homotypic RBD-nanoparticle. (**G**) HSW-2 IgG interacting with adjacent RBDs on a homotypic RBD-nanoparticle.

Supplemental Tables

mAbs	V_H (sequences)	V_L (sequences)	Lineages
**M8a-3**	QVQLQQPGAELVLPGASVKLSCKASGYTFTNYWMHWVKQRPG<br>HGLEWIGEIDPFDTYIKINQKFKGKSTLTVDTSSSTAYMQLS<br>SLTSEDSAVYYCARPDSSGYPVYFDYWGQGTTLTVSS	DIVMTQSHKFMSTSVGDRVSITCKASQDVGTYIAWYQQKPGQ<br>SPKLLIYWASTRHTGVPDRFTGSGSGTNYTLTISSVQAEDLA<br>LYHCQQHYSTPYTFGGGTKLEIK	IgHV1-69<br>IgκV6-25
**M8a-6**	QVQLQQPGTELVMPGASVKLSCKTSGYTFTHYWMHWVKQRPG<br>EGLEWIGEIAPSDNYVKYNQKFKGKSTLSVDRSSSTAYMQLS<br>SLTSEDSAVYFCARPDNSGYPVYFDYWGQGTSLTVSS	DIVMTQSQKFMSTSLGDRVSISCKASQDVGTTVAWYQQKPGQ<br>SPKLLIYWASTRHTGVPDRFTGTGSGTDYTLTISSVAAEDLA<br>LYYCQQHYNTPYTFGGGTKLEIE	IgHV1-69<br>IgκV6-25
**M8a-7**	QAYLQQSGAEMVRPGASVKMSCKASGYTFNNYNMHWVKQTPS<br>QGLEWIGGFYPGNDDTAYSQKFKGKATLTVDKSSSTAFMHLS<br>SLTSEDSAVYFCARSLGRYYAMDYWGQGTSVTVSS	DIVLTQSPASLAVSLGQRATISCRASESVDDFGISYMNWFQQ<br>KPGQTPKLLIYGASNQGSGVPARFSGSGSGTDFSLNIHPMEE<br>DDPAMYFCQQSKEVPYTFGGGTKLEIK	IgHV1-12<br>IgκV3-2
**M8a-9**	QVQLQQPGAELVRPGSSVKLSCKASGYTFTSYWIHWVRQRPI<br>EGPEWIGMIDPSDSGNHFNQNFKDKATWTVDKSSNTAYMQLS<br>SLTTEDSAVYYCARGSGSTYRGYFDYWGHGTTLTVSS	DIQMTQSSSYLSVSLGGRVTITCKASDHINNWLAWYQQKPGN<br>TPRLLISGATNLETGVPSRFSGSGSGKDYTLSITSLQTEDVA<br>TYYCQQYWSSPLTFGAGTKLELK	IgHV1-52<br>IgκV13-85
**M8a-11**	EVQLQQSGPELVKPGASVKIPCKASGYTFTDYNMDWVKQSHE<br>KSLEWIGEIDPNNGDTIYNQKFKGKASLTVDKSSSTAYMELR<br>SLTSEDTAVYYCAKRGYYGSSLWWYFDVWGTGTTVTVSS	DIQMTQSSSSFSVSLGDRVTITCKASEDIYIRLAWYQQRPGN<br>APRLLISNAISLETGVPSRFSGSGSGKDYTLSITSLQTEDVA<br>TYYCQQYWSTPWTFGGGTKLEIK	IgHV1-18<br>IgκV13-84
**M8a-15**	QVQLQQSGPELARPGASVKLSCRASGYTVTSFGLSWMKQRTG<br>QGLEWIGEIYPTSKNTYYNDKFRTKATLTADKSSSTAYMELR<br>SLTSEDSAVYFCVLYDYFDYWGQGTTLTVSS	QIVLTQSPAIMSASPGEKVTISCSASSSVSYMYWYQQKTGSS<br>PKPWIYRTSNLASGVPVRFSGSGSGTSYSLTISSMEAEDAAT<br>YYCQQYQSYPRTFGGGTKLEIK	IgHV1-81<br>IgκV4-61
**M8a-25**	EVQLQQSVAELVRPGASVKLSCTASGFNIKNTYMHWVKQRPE<br>QGLEWIGRIDPSIDHTRYAPKFQGKAVITAFTSSNTAYLQLS<br>SLTSEDTAIYYCAREGGGNYPYYYAIDYWGQGTSVTVSS	DIQMTQSSSSFSVSLGDRVTITCKASEDIYIRLAWYQQRPGN<br>APRLLISNAISLETGVPSRFSGSGFGKDHTLSITSLQTEDVA<br>TYYCQQYWSTPWTFGGGTKLEIK	IgHV14-3<br>IgκV13-84
**M8a-28**	QVQLQQPGAELVKPGASVKMSCKASGYNFNHYWISWVKQRPG<br>QGLEWIGDIYPLSHFTTYNEKFTNRATLTVDTSSTTAYMQLN<br>SLTSDDSAVFYCARWDYFDSRTFDYWGQGTTLTVSS	DILLTQFPAILSVSPGERVSFSCRASQTIGTNIHWYQQRING<br>SPRLLIKYASESISGIPSRFSGSGSGTDFSLSINNVESEDIA<br>DYYCQQINSWPLTFGAGTKLDLK	IgHV1-55<br>IgκV5-48
**M8a-29**	EVQLQQSVAELVRPGASVKLSCTASGFNIKNTYMHWVKQRPE<br>QGLEWIGRIDPSIDHTRYAPKFQGKAVITAFTSSNTAYLQLS<br>SLTSEDTAIYYCAREGGGNYPYYYAIDYWGQGTSVTVSS	DIVMTQAAFSNPVTLGTSASISCRSTKSLLHSNGITYLYWYL<br>QKPGQSPQLLIYQMSNLASGVPDRFSSSGSGTDFTLRISRVE<br>AEDVGVYYCAQNLELPYTFGGGTKLEIK	IgHV14-3<br>IgκV2-109
**M8a-30**	QVHLQQSGPELVKPGASVKISCKASGYGFSSSWMNWVKQRPG<br>KGLEWIGRIYPGDGDTNYNDKFQGKATLTADRSSSTAYMHLT<br>SLTSADSAVYFCARSLLYSFDYWGQGTTLTVSS	DVVVTQTPLSLPVSFGDQVSISCRSSQSLAGSYGHTYLSWYL<br>HKSGQSPQLLIYGISNRFSGVPDRFSGSGSGTDFTLKISTIK<br>PEDLGMYYCLQGTHQPLTFGAGTKLELK	IgHV1-82<br>IgκV1-88
**M8a-31**	EVQLKQSVAELVRPGASVKVSCTASGFNIKNIYMHWVKQRPE<br>QGLDWIGRIDPANGNSRYAPKFQDKATITADTSSNTAYLQLS<br>SLTSEDTAIYYCADEGWGFANWGQGTLVTVSA	DIVMTQSPSSLTVTAGEKVTMSCKSSQSLLNSGNQKNYLTWY<br>QQKVGQPPKLLIYWASTRDPGVPDRFTGSGFGTDFTLTISSV<br>QAEDLAVYYCQNDYSYPLTFGAGTKVELK	IgHV14-3<br>IgκV8-19
**M8a-34**	QVQLQQPGAELVKPGASVKMSCKASGYTFITYWITWVKQRPG<br>QGLEWIGDIYPGGGRTNYNEKFKSKATLTVDTSSSTAYMQLR<br>SLTSEDSAVYYCARYDGNYVGYYYAMDYWGQGTSVTVSS	DIVLTQSPVSLAVSLGQRATISCRASESVDFYGNSFIYWYQQ<br>KPGQAPKLLIYRASNLESGIPARFSGSGSRTDFTLTIHPVEA<br>DDVATYYCQQSIEDPRTFGGGTKLEIK	IgHV1-55<br>IgκV3-5
**M8a-36**	QVQLQQTGAELVKPGASVKMSCKASGYTFTSYWIIWVKKRPG<br>QGLEWIGDIYPGSGSTNYNEKFKSKATLTVDTSSSTAYMQLS<br>SLTSEDSAVYYCTRGGSRFAMDYWGQGTSVTVSS	DIVMTQAAPSVPVTPGESVSISCRSSKSLLHSNGNTYLYWFL<br>QRPGQSPQLLIYRMSNLASGVPDRFSGSGSGTAFTLRISRVE<br>AEDVGVYYCMQHLEYPYTFGGGTKLEIK	IgHV1-55<br>IgκV2-137
**HSW-1**	QVQLQQPGAELVKPGTSMKLSCKASGYTFTSYWMHWVKQRPG<br>QGLEWIGMIHPNSGSTKYNENFKSKATLTVDKSSSTAYMQFS<br>SLTSEDSAVYYCVRSGSYYGTTYDYFDYWGQGTTLTVSS	DIVLTQSPASLAVSLGQRATISCRASESVNIYGNSFMHWYQQ<br>KPGQPPKLLIFRASNLESGIPVRFSGSGSRTDFTLTINPVEA<br>DDVATYYCHQSNEDPFTFGSGTKLEIK	IgHV1-64<br>IgκV3-5
**HSW-2**	QVQLQQSGPELVKPGASVKISCKASGYVFSTSWMSWVKQRPG<br>EGPEWIGRIYPRDGHSSSTGKFKDKATLTADKSSNTAYIHLS<br>SLTSEDSAVYFCARDYGYYYFDYWGQGTTLTVSS	DIQMTQSPASLSASVGEAVTITCRLSENVYSFLAWYQQKQGK<br>SPQLLVYRAKTLAEGVPSRFSGSGSGTQFSLKINSLQPEDFG<br>TYYCQHHYGTPPTFGGGTKLEIK	IgHV1-82<br>IgκV12-44

Table S1. Sequences and V gene segment lineages for 16 mAbs identified as binding at least one RBD during screening. Related to Figure 2. M8a-9 and M8a-36 did not exhibit binding to purified RBDs by ELISA (data not shown). M8a-11 and M8a-26 are identical sequences.

Structures	WA1 spike in complex with							Omicron BA.1 spike in complex with M8a-31 Fab
	M8a-3 Fab	M8a-6 Fab	M8a-28 Fab	M8a-31 Fab	M8a-34 Fab	HSW-1 Fab	HSW-2 Fab	
**Data Collection and processing**								
**Microscope**	Titan Krios	Titan Krios	Titan Krios	Titan Krios	Titan Krios	Titan Krios	Talos Arctica	Titan Krios
**Camera**	Gatan K3	Gatan K3	Gatan K3	Gatan K3	Gatan K3	Gatan K3	Gatan K3	Gatan K3
**Magnification**	105,000	105,000	105,000	105,000	105,000	105,000	45,000	105,000
**Voltage (keV)**	300	300	300	300	300	300	200	300
**Exposure (e/Å^2)**	60	60	60	60	60	60	60	60
**Pixel size (Å)**	0.832	0.832	0.832	0.832	0.832	0.832	0.869	0.832
**Defocus Range (μm)**	-1 to -3	-1 to -3	-1 to -3	-1 to -3	-1 to -3	-1 to -3	-1 to -3	-1 to -3
**Initial Particle Image (no.)**	1,026,209	842,602	964,614	670,746	1,186,807	1,713,001	449,736	1,689,653
**Final Particle Image (no.)**	272,779	159,604	332,106	295,711	421,879	336,288	43,362	142,452
**Symmetry Imposed**	C1	C1	C3	C3	C1	C1	C1	C3
**Map Resolution (Å)**	3.1	3.2	2.8	2.9	3.5	3.1	4.1	3.1
**FSC Threshold**	0.143	0.143	0.143	0.143	0.143	0.143	0.143	0.143
**Map Resolution Range (Å)**	3.0 - 3.5	3.1 - 3.5	2.7 to 3.1	2.9 - 3.2	3.5 - 4.3	3.0 to 3.4	3.9 - 4.3	2.9 - 3.3
**Refinement**								
**Initial Model Used**	PDB 7SC1	PDB 7SC1	PDB 7SC1	PDB 7SC1	PDB 7SC1	PDB 7SC1	PDB 7SC1	PDB 7SC1
**Model Resolution (Å)**	3.1	3.2	2.8	2.9	3.5	3.1	4.1	3.1
**FSC Threshold**	0.143	0.143	0.143	0.143	0.143	0.143	0.143	0.143
**Model composition**								
**non-hydrogen atoms**	30,762	27,051	30,942	30,552	30,669	26,992	8,392	30,075
**protein residues**	3,867	3,397	3,906	3,861	3,873	3,409	1,077	3,801
**ligands**	51	48	39	45	42	38	4	33
**Average B-factors (Å^2)**								
**protein**	135.2	157.5	110.4	130.4	205.7	158.8	197.9	92.4
**ligands**	116.3	139.9	96.8	111.6	167.3	143.6	213.5	82.5
**R.m.s. deviations**								
**Bond length (Å)**	0.005	0.004	0.003	0.004	0.003	0.004	0.003	0.004
**Bond angles (º)**	0.562	0.582	0.536	0.572	0.586	0.593	0.589	0.594
**Validation**								
**MolProbity score**	1.85	1.63	1.74	1.72	1.75	1.83	1.9	1.77
**Clashscore**	10.3	9.4	9.5	9.1	11.1	12.3	14.6	10.8
**Rotamer outliers**	0.06	0.07	0.03	0	0.03	0	0	0.06
**Ramachandran plot**								
**Ramachandran favored (%)**	95.4	97.3	96.4	96.5	96.9	96.5	96.4	96.6
**Ramachandran allowed (%)**	4.6	2.7	3..6	3.5	3.1	3.5	3.6	3.4
**Ramachandran outliers (%)**	0	0	0	0	0	0	0	0
**PDB ID**	7UZ4	7UZ5	7UZ6	7UZ7	7UZ9	7UZA	7UZB	7UZ8

Table S2. Single-particle cryo-EM data collection, processing, and refinement. Related to Figure 3 and 4.

Structures	SARS-CoV-2 RBD + M8a-34 Fab	SARS-CoV-2 RBD + HSW-2 Fab
Wavelength (Å)	0.97946	0.97946
Resolution range (Å)	39.23 - 2.2 (2.279 - 2.2)	39.08 - 3.0 (3.108 - 3.0)
Space group	$P2_1$	$P4_32_12$
Unit cell (Å, °)	54.5 165.2 93.7 90 106.6 90	123.6 123.6 145.6 90 90 90
Total reflections (no.)	553,434 (52,800)	592,837 (59,309)
Unique reflections (no.)	79,107 (7,859)	23,174 (2,178)
Multiplicity	7.0 (6.7)	25.6 (26.2)
Completeness (%)	98.5 (97.9)	99.5 (96.1)
Mean I/sigma(I)	12.2 (1.24)	19.6 (2.6)
Wilson B-factor ($Å^2$)	41.4	81.1
R-merge	0.105 (1.378)	0.147 (1.856)
R-meas	0.113 (1.494)	0.150 (1.893)
R-pim	0.0425 (0.568)	0.030 (0.366)
CC1/2	0.998 (0.642)	0.999 (0.842)
CC*	1 (0.884)	1 (0.956)
Reflections used in refinement (no.)	79,082 (7,845)	23,082 (2,178)
Reflections used for R-free (no.)	1,997 (195)	1,155 (109)
R-work	0.171 (0.270)	0.230 (0.360)
R-free	0.243 (0.329)	0.271 (0.458)
CC (work)	0.971 (0.821)	0.899 (0.834)
CC (free)	0.962 (0.771)	0.604 (0.753)
Number of non-hydrogen atoms	10,482	4,912
macromolecules	9,884	4,884
ligands	111	28
solvent	487	0
Protein residues (no.)	1,278	633
RMS (bonds) (Å)	0.008	0.009
RMS (angles) (°)	0.98	1.15
Ramachandran favored (%)	97.5	96.3
Ramachandran allowed (%)	2.5	3.7
Ramachandran outliers (%)	0	0
Rotamer outliers (%)	0.9	0
Clashscore	2.6	6.1
Average B-factor ($Å^2$)	47	73.0
macromolecules	46.7	72.7
ligands	85.0	118.3
solvent	44.3	-
PDB ID	7UZC	7UZD

Table S3. X-ray crystallography data collection, processing, and refinement. Related to Figure 3 and 4.

References

Barnes, C.O., Jette, C.A., Abernathy, M.E., Dam, K.A., Esswein, S.R., Gristick, H.B., Malyutin, A.G., Sharaf, N.G., Huey-Tubman, K.E., Lee, Y.E., *et al*. (2020). SARS-CoV-2 neutralizing antibody structures inform therapeutic strategies. Nature *588*, 682-687.

Krissinel, E., and Henrick, K. (2007). Inference of macromolecular assemblies from crystalline state. J Mol Biol *372*, 774-797.

Lefranc, M.P., Giudicelli, V., Duroux, P., Jabado-Michaloud, J., Folch, G., Aouinti, S., Carillon, E., Duvergey, H., Houles, A., Paysan-Lafosse, T., *et al*. (2015). IMGT(R), the international ImMunoGeneTics information system(R) 25 years on. Nucleic Acids Res *43*, D413-422.
